# Metal-free, room temperature, acid-K_2_S_2_O_8_ mediated method for the nitration of olefins: an easy approach for the synthesis of nitroolefins†

**DOI:** 10.1039/c9ra06414a

**Published:** 2019-09-25

**Authors:** Srinivas Ambala, Rohit Singh, Maninder Singh, Pankaj Singh Cham, Ria Gupta, Gurunadham Munagala, Kushalava Reddy Yempalla, Ram A. Vishwakarma, Parvinder Pal Singh

**Affiliations:** Medicinal Chemistry Division, CSIR-Indian Institute of Integrative Medicine India ppsingh@iiim.ac.in; Academy of Scientific and Innovative Research India

## Abstract

Here, we have developed a simple, room temperature method for the nitration of olefins by using inexpensive sodium nitrite as a source of nitro groups in the presence of trifluoroacetic acid (TFA) and potassium persulfate (K_2_S_2_O_8_) under an open atmosphere. Styrenes and mono-substituted olefins give stereo-selective corresponding *E*-nitroolefins under optimized conditions, however, 1,1-bisubstituted olefins give a mixture of *E*- and *Z*-nitroolefins. The optimized conditions work well with electron-donating, electron-withdrawing, un-substituted and heterocyclic styrenes and mono-substituted olefins and give corresponding nitroolefins with good to excellent yields.

## Abstract 2

Here, we have developed a simple, room temperature method for the nitration of olefins by using inexpensive sodium nitrite as a source of nitro groups in the presence of TFA and potassium persulphate under an open atmosphere.

## Graphical abstract

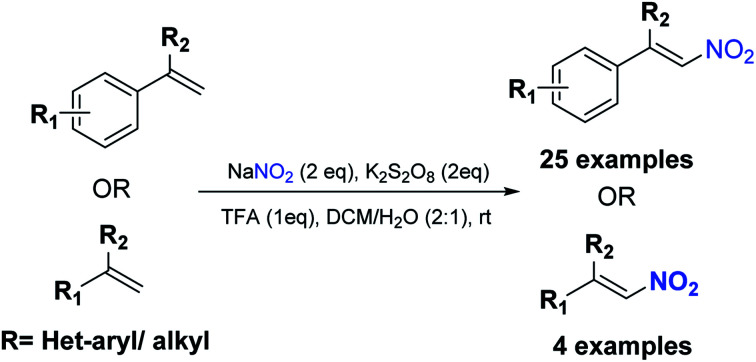

## Introduction

**Fig. 1:**
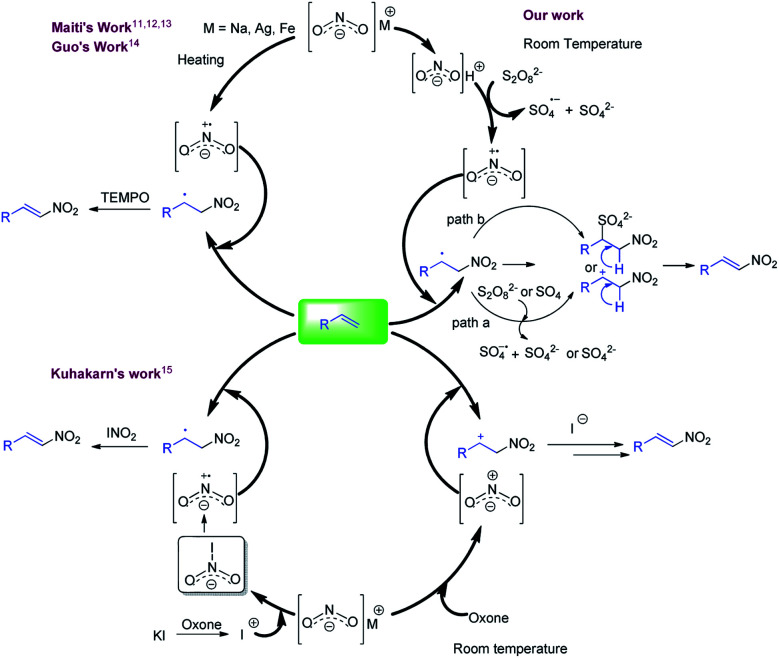
Literature precedent and present method for nitration of olefins.

Nitroolefins are intriguing synthetic units and have been used for the generation of biological, pharmaceutical, materials, and agrochemical relevant molecules.^1*a*–*e*^ Moreover, these are used as starting materials for various C–C bond forming reactions^2–4^ and have been utilized in the synthesis of diverse chemically interesting molecules, *viz*., oximes, hydroxylamines, nitroalkanes, aliphatic amines, and nitroso compounds.^5*a*,*b*^ Accordingly, considerable efforts have been made in the synthesis of nitroolefins including the Henry reaction, which involves the base mediated condensation of an aldehyde or ketone with a nitroalkane.^6^ The other approach used for the synthesis of nitroolefins involved the nitration of olefins using a mixture of HNO_3_ and conc. sulfuric acids, where the reaction efficiency is significantly hampered by the production of a large amount of acid waste as well as nitrogen oxide fumes.^7^ Alternatively, nitro alkenes are also synthesized by the decarboxylative nitration of α,β-unsaturated carboxylic acids.^8*a*,*b*,9^ However, the direct incorporation of a nitro group on an olefin is considered as the preferred approach. In this direction, several synthetic methods have been reported employing transition metals and gaseous nitrating agents. These methods offer significant improvements over the previous methods but the reaction always suffer with the formation of undesired *E*/*Z* isomeric mixture of product.^10*a*–*d*^ To address this, over the decade, many synthetic groups have also reported stereo-selective nitration of olefins. In this context, Maiti and co-workers reported the stereo-selective nitration of aliphatic and aromatic olefins with *in situ* generated NO_2_ centred radical from the nitrites (AgNO_2_, Fe(NO_3_)_3_ or *t*-BuONO) in the presence of TEMPO (Fig. 1, right side, upper half).^11–14^ In the similar pattern, Guo *et al.* also developed another method using combination of sodium nitrite (NaNO_2_), potassium persulfate and TEMPO (Fig. 1, right side, upper half).^14^ In another attempt, Kuhakarn *et al.* reported the stereo-selective nitration of olefin using radical based species generating from the reaction of sodium nitrite (NaNO_2_), oxone and potassium iodide (Fig. 1, lower half).^15^ All these stereo-selective methods are productive, but either employed high temperature or stoichiometric amounts of reagents such as TEMPO.

Considering the application of nitroolefins, development of an efficient, mild and practical method for the nitration of olefin is highly desirable. The present concept is developed considering the stability of nitrite where it is well known that nitrous acid is highly reactive in comparison to their metal salt which may initiate the reaction at room temperature. Upon several attempts, a mild and room temperature method was developed for the nitration of alkenes in the combination of sodium nitrite (NaNO_2_) and potassium persulfate (K_2_S_2_O_8_) and trifluoracetic acid (TFA) which offered high stereo-selectivity for styrenes and mono-substituted olefins (Fig. 1, left side, upper half).

## Results and discussion

Initially, we started the study with styrene 1a in the presence of NaNO_2_ and K_2_S_2_O_8_ as a model substrate for the nitration. Screening of the reaction conditions are shown in Table 1. First reaction was performed by reacting 1a (1.0 mmol) with NaNO_2_, K_2_S_2_O_8_ and TFA in DCM under open atmosphere, the formation of required product, (*E*)-(2-nitrovinyl)benzene 2a was observed with the yield of 34% (entry 1). In a next attempt, we conducted the reaction in a mixture of DCM : water, significant improvement in the yield of 2a from 34 to 71% was observed (entry 2). However, the reaction in water alone didn't seem good which afforded product 2a in a yield of 32% (entry 3). In another attempts with other solvent systems such as acetone : water, acetonitrile (ACN) : water, 1,2-dichloroethane (DCE) : water, the reaction underwent but with comparatively lower yield except DCE : water which gave almost similar results as obtained with DCM : water (entries 4–6). Surprisingly, no reaction was observed in the absence of TFA (entry 7). With increase amount of K_2_S_2_O_8_, the formation of 2a was observed in a yield of 62%, the increased amount might cause degradation of the product (entry 8). Lower yield of the 2a was observed at higher temperature 70 °C (entry 10). Next, the effect of TFA concentrations was also studied (entries 11 & 12), where both increase and decrease in its concentration affect the yield and best result was obtained with one equivalent of TFA. When the concentration of NaNO_2_ was decreased, the formation of 2a also gets affected drastically. When the oxidant K_2_S_2_O_8_ was replaced with other organic oxidant such as *tert*-butylhydroperoxide (TBHP), only traces amount of required product was observed (entry 14).

**Table 1:** Optimization studies^a^

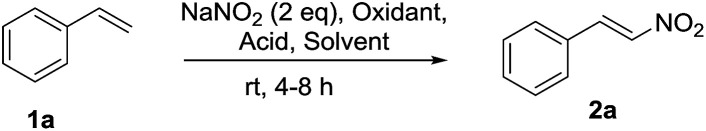					
Entry	Oxidant (equiv.)	Acid (equiv.)	Solvent	Time (h)	Yield^a^ (%)
1	K_2_S_2_O_8_ (2)	TFA (1)	DCM	24	34
2	K_2_S_2_O_8_ (2)	TFA (1)	DCM/H_2_O (2 : 1)	24	67
3	K_2_S_2_O_8_ (2)	TFA (1)	H_2_O	4	32
4	K_2_S_2_O_8_ (2)	TFA (1)	Acetone/H_2_O (2 : 1)	4	38
5	K_2_S_2_O_8_ (2)	TFA (1)	ACN/H_2_O (2 : 1)	4	43
6	K_2_S_2_O_8_ (2)	TFA (1)	DCE/H_2_O (2 : 1)	4	68
7	K_2_S_2_O_8_ (2)	—	DCM/H_2_O (2 : 1)	24	—
8	K_2_S_2_O_8_ (2)	TFA (1)	DCM/H_2_O (2 : 1)	24	62
**9**	**K**_**2**_ **S**_**2**_ **O**_**8**_ **(2)**	**TFA (1)**	**DCM/H**_**2**_ **O (2** **:** **1)**	**4**	**71**
10^b^	K_2_S_2_O_8_ (2)	TFA (1)	DCM/H_2_O (2 : 1)	4	51
11	K_2_S_2_O_8_ (2)	TFA (0.5)	DCM/H_2_O (2 : 1)	4	48
12	K_2_S_2_O_8_ (2)	TFA (1.2)	DCM/H_2_O (2 : 1)	4	69
13^c^	K_2_S_2_O_8_ (2)	TFA (1)	DCM/H_2_O (2 : 1)	4	44
14	TBHP (2)	TFA (1)	DCM/H_2_O (2 : 1)	4	Trace

a Reaction conditions: styrene (1 mmol), ^a^isolated yields, ^b^reaction carried at 70 °C, ^c^with one equivalent of NaNO_2_.

**Scheme 1:**
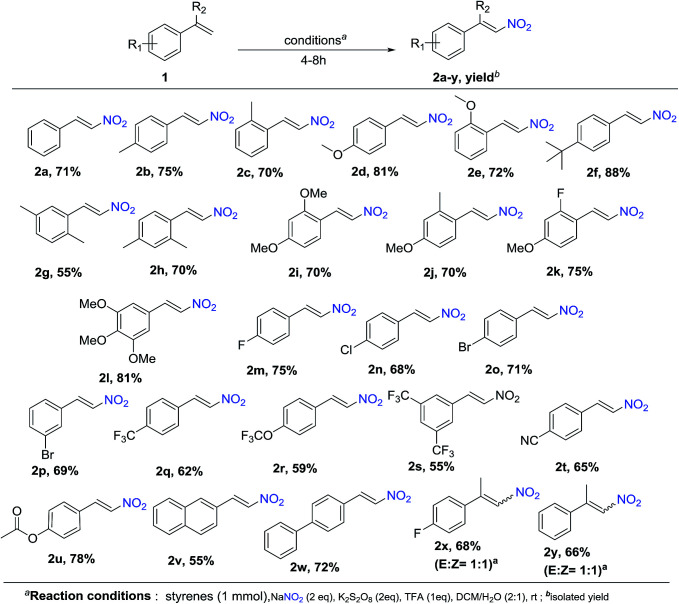
Nitration of styrenes.

**Scheme 2:**
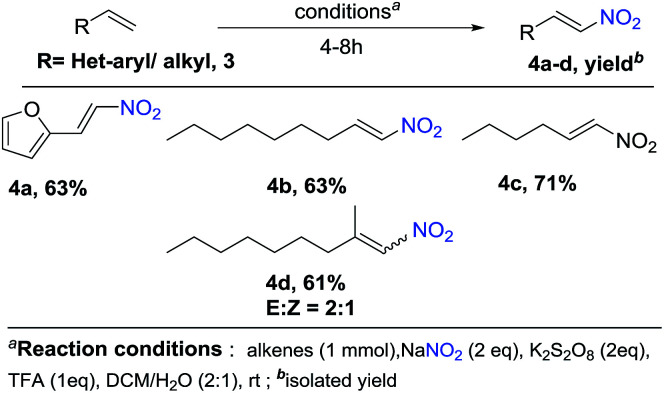
Nitration of vinyl heteroarenes and aliphatic olefins.

To explore the scope of the reaction, as well as its suitability for the preparation of substituted nitroolefins, a variety of styrenes having electron-withdrawing as well as electron-donating groups were tried under optimized condition. As shown in Schemes 1 and 2, reaction conditions were compatible to all the substituted styrenes, and transformed into the corresponding *E*-β-nitro olefin in moderate to excellent yields (55–88%). The nature and position of the substituent had shown some influence on the reaction. The mono-substituted styrenes having electron-donating groups like –Me, –OMe and *t*-butyl at C4 position gave the desired nitro product in better yields compared with C2 position (2b-75%, 2c-70%, 2d-81%, 2e-72%, and 2f-88%). Furthermore, substrates such as di- and tri-substituted styrenes were also found to be the suitable substrates and afford the corresponding products in good yields (2g-55%, 2i-74%, 2j-75%, 2k-75%, and 2l-81%). It is important to note that diminished yields were observed with various styrene derivatives having electron-withdrawing groups (–F, –Cl, –Br, –CF_3_, –CN) 2m-75%, 2n-68%, 2o-71%, 2p-69%, 2q-62%, 2r-59%, 2s-55%, and 2t-65%). Furthermore, styrene substituted with labile functional groups like ester also underwent smooth coupling and converted to the corresponding nitrostyrene 2u with 78% yield. Next, under the optimized conditions, we investigated the nitration of vinylnapthalene and biphenyl styrene, which affords the nitration product 2v and 2w in a yield of 55% and 70%, respectively. Moreover, the reaction with α,α-bisubstituted styrenes gave *E* : *Z* mixture of the corresponding nitrated products 2x and 2y in 68% and 66% yield, respectively.

In addition, we also expanding the present reaction condition to vinyl heterocycles, where 2-vinylfuran on attempt afforded the nitration product 4a in a yield of 63%. Moreover, aliphatic olefins such as nonene and hexene when tried, reacted smoothly and gave corresponding products 4b and 4c in a yield of 63% and 71%, respectively. However, α,α-bisubstituted olefins gave a corresponding product 4d in a yield of 61% with *E*/*Z* ratio of 2 : 1 (Schemes 1 and 2).

**Scheme 3:**
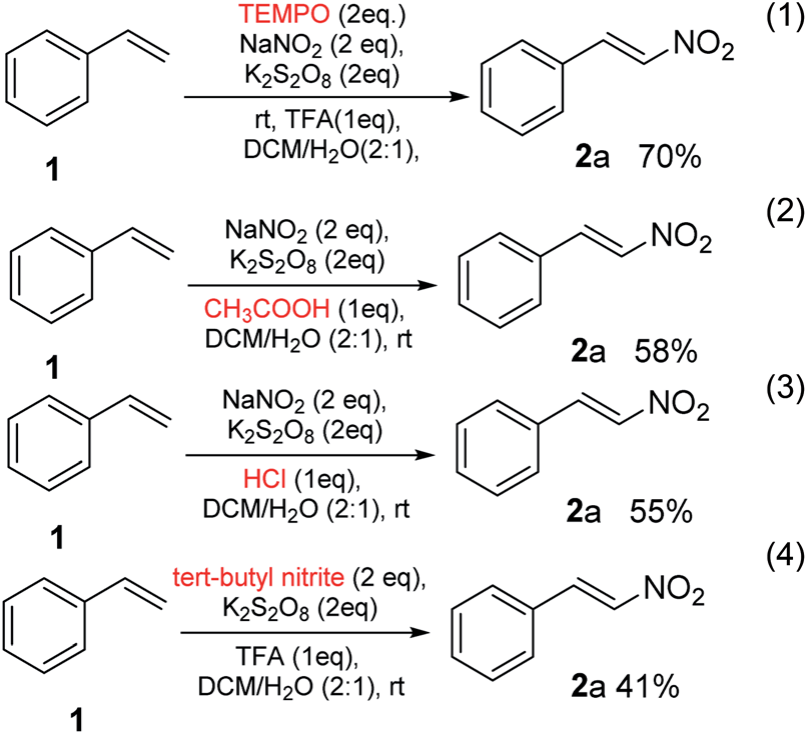
Nitration in different condition.

When the reaction of styrene 1, was performed under the optimized conditions in the presence of free-radical scavenger, TEMPO, nitro-substituted styrene 2a was obtained in a yield of 70% (eqn (1), Scheme 3), no suppression was observed. It might be due to the TEMPO-adduct undergo elimination and produced nitro-containing product in a similar fashion reported by Maity *et al.*^13^ In the next experiment, when the proton source was changed and used acetic acid and HCl, the reactions underwent and the gave the product 2a in a yield of 58% and 55%, respectively (eqn (2) and (3), Scheme 3). In the next part, NO_2_ source was also changed and used *tert*-butyl nitrite in place of sodium nitrite which gave the required product in a yield of 41%. These results further expands the utility of method.

## Conclusion

In summary, we have developed a simple, room temperature method for the nitration of olefins with sodium nitrite, TFA and potassium persulphate under open atmosphere. This method give high stereo-selective *E*-nitroolefins with styrenes and mono-substituted olefins. The use of diverse range of acids and nitro-sources provide additional advantage. The study with other substrates and application is under progress and will be published in due course.

## Conflicts of interest

There are no conflicts to declare.

## Supplementary Material

RA-009-C9RA06414A-s001

## References

[cit1] Reddy M. A., Jain N., Yada D., Kishore C., Reddy V. J., Reddy P. S., Addlagatta A., Kalivendi S. V., Sreedhar B. (2011). J. Med. Chem..

[cit2] Basavaiah D., Reddy B. S., Badsara S. S. (2010). Chem. Rev..

[cit3] Berner O. M., Tedeschi L., Enders D. (2002). Eur. J. Org. Chem. Rev..

[cit4] Denmark S. E., Thorarensen A. (1996). Chem. Rev..

[cit5] Quan X.-J., Ren Z.-H., Wang Y.-Y., Guan Z.-H. (2014). Org. Lett..

[cit6] Ballini R., Bosica G. (1997). J. Org. Chem..

[cit7] Tinsley S. W. (1961). Iminosulphur oxydifluoridrs. J. Org. Chem..

[cit8] Manna S., Jana S., Saboo T., Maji A., Maiti D. (2013). Chem. Commun..

[cit9] Das J. P., Sinha P., Roy S. (2002). Org. Lett..

[cit10] Jovel I., Prateeptongkum S., Jackstell R., Vogl N., Weckbecker C., Beller M. (2008). Adv. Synth. Catal..

[cit11] Maity S., Manna S., Rana S., Naveen T., Mallick A., Maiti D. (2013). J. Am. Chem. Soc..

[cit12] Naveen T., Maity S., Sharma U., Maiti D. (2013). J. Org. Chem..

[cit13] Maity S., Naveen T., Sharma U., Maiti D. (2013). Org. Lett..

[cit14] Zhao A., Jiang Q., Jia J., Xu B., Liu Y., Zhang M., Liu Q., Luo W., Guo C. (2016). Tetrahedron Lett..

[cit15] Hlekhlai S., Samakkanad N., Sawangphon T., Pohmakotr M., Reutrakul V., Soorukram D., Jaipetch T., Kuhakarn C. (2014). Eur. J. Org. Chem..

