# Supplementary material for: Metal-free, room temperature, acid-K2S2O8 mediated method for the nitration of olefins: an easy approach for the synthesis of nitroolefins
Source: RSC Adv. 2019 Sep 25;9(52):30428–31. doi: 10.1039/c9ra06414a (PMC9072150; doi:10.1039/c9ra06414a)
Supplement: RA-009-C9RA06414A-s001 [file RA-009-C9RA06414A-s001.pdf]

Electronic Supplementary Material (ESI) for RSC Advances.
This journal is © The Royal Society of Chemistry 2019

Supporting Information

Metal-Free, Room Temperature, Acid-$K_2S_2O_8$ Mediated Method for the Nitration of Olefins: An Easy Approach for the Synthesis of Nitroolefins

Srinivas Ambala[a,b,†], Rohit Singh[a,b,†], Maninder Singh[a], Pankaj Singh Cham[a], Ria Gupta[a,b], Gurunadham Munagala[a,b], Kushalava Reddy Yempalla[a,b], Ram A. Vishwakarma[a,b] and Parvinder Pal Singh[a,b*]

[a]Medicinal Chemistry Division, CSIR-Indian Institute of Integrative Medicine, Canal Road, Jammu-180001, India; [b]Academy of Scientific and Innovative Research, Canal Road, Jammu-180001, India

Table of Content

1. **Materials and Methods** **S-2**
2. **General Experimental Procedure** **S-2**
3. **Characterization Data of Products** **S-3 to S-13**
4. **Spectras of Synthesized Products** **S-14 to S-83**
5. **References** **S-84**

Materials and Methods

All reactions were performed under air atmosphere. Analytical thin layer chromatography was performed using TLC pre-coated silica gel 60 F_{254} (20 x 20 cm). TLC plates were visualized by exposing UV light or by iodine vapors or immersion in an acidic staining solution of p-anisaldehyde followed by heating on a hot plate. Organic solvents were concentrated by rotary evaporation. Column chromatography was performed on flash silica gel of 230-400 mesh size. Melting points were recorded on BUCHI Melting Point B-545 instrument and were uncorrected. ^{1}H NMR spectra were recorded with 400 and 500 MHz NMR instruments. Chemical data for protons are reported in parts per million (ppm, scale) downfield from tetramethylsilane and are referenced to the residual proton in the NMR solvent ($CDCl_3$: δ 7.26 or other solvents as mentioned). Mass spectra were recorded with LCMS-QTOF instrument. The coupling constant (J) are mentioned in Hz.

General Experimental Procedure for the Synthesis of Compounds in Table 2 and Table 3:

Styrene/alkene (1 mmol), $NaNO_2$ (2 mmol), $K_2S_2O_8$ (2 mmol) and TFA (1 mmol) in 4.5 ml of DCM/water (2:1) were stirred in open atmosphere at room temperature for 6 h. After completion of the reaction (reaction monitored by TLC) compound was extracted with DCM. The combined organic layer was dried over anhydrous Na_2SO_4 and concentrated under reduced pressure. The crude products were purified on a silica gel column using hexane/EtOAc to get the pure product and characterized by NMR and GC-MS.

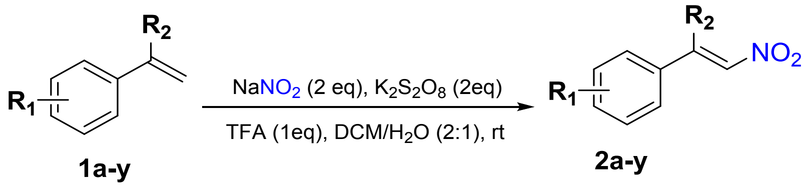

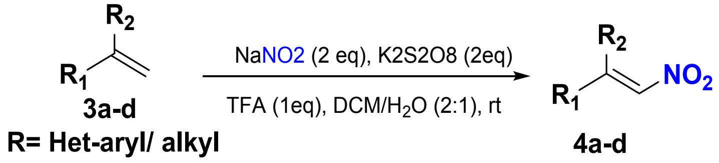

Characterization Data of Products

(*E*)-(2-Nitrovinyl)benzene (2a)

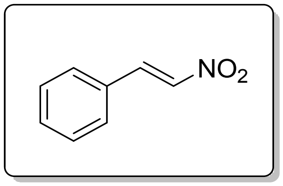

Light yellow solid; TLC (EtOAc:Hexane 2:8): R_f = 0.20; mp 57-58 °C; ^{1}H NMR (400 MHz, $CDCl_3$) δ 8.02-7.98 (d, J = 13.7 Hz, 1H), 7.61-7.57 (d, J = 13.7 Hz, 1H), 7.56–7.53 (m, 2H), 7.50-7.43 (m, 3H); ^{13}C NMR (126 MHz, $CDCl_3$) δ 139.17, 137.11, 132.23, 130.06, 129.44, 129.21; GC-MS (m/z): 149.1 $[M]^+$.

***(E*)-1-Methyl-4-(2-nitrovinyl)benzene (2b)**

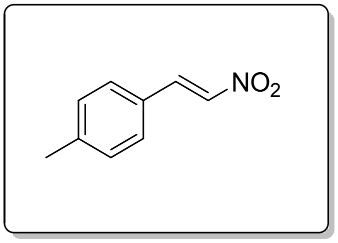

Light yellow solid; TLC (EtOAc:Hexane 2:8): R_f = 0.20; mp 221-223 ^{o}C; ^{1}H NMR (400 MHz, $CDCl_3$) δ 7.98-7.94 (d, J = 13.6 Hz, 1H), 7.57-7.54 (d, J = 13.6 Hz, 1H), 7.44-7.42 (d, J = 8 Hz, 2H), 7.26-7.24 (d, J = 8.0 Hz, 2H), 2.40 (s, 3H). ^{13}C NMR (101 MHz, $CDCl_3$) δ 143.18 (1C), 139.20 (1C), 136.31 (1C), 130.19 (2C), 129.26 (2C), 127.32 (1C), 21.68 (1C) ; GC-MS (m/z): 163.10 $[M]^+$.

(*E*)-1-Methyl-2-(2-nitrovinyl)benzene (2c)

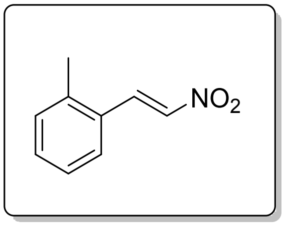

Light yellow liquid; TLC (EtOAc:Hexane 2:8): R_f = 0.20; ^{1}H NMR (400 MHz, $CDCl_3$) δ 8.30-8.27 (d, J = 13.6 Hz, 1H), 7.51–7.46 (m, 2H), 7.38-7.28 (m, 1H), 7.26-7.23 (m, 2H), 2.47

(s, 3H); ^{13}C NMR (126 MHz, $CDCl_3$) δ 139.28, 137.60, 136.78, 132.00, 131.41, 128.92, 127.38, 126.80, 19.94; GC-MS (m/z): 163.10 $[M]^+$.

(*E*)-1-Methoxy-4-(2-nitrovinyl)benzene (2d)

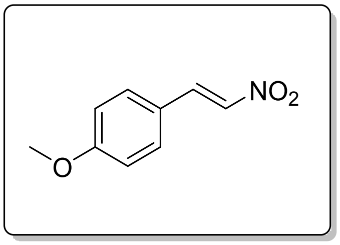

Light yellow solid; TLC (EtOAc:Hexane 2:8): R_f = 0.20; mp 87-90 °C; ^{1}H NMR (400 MHz, $CDCl_3$) δ 7.99-7.96 (d, J = 13.6 Hz, 1H), 7.54-7.50 (m, 3H), 6.97-6.95 (d, J = 8.8 Hz, 2H), 3.87 (s, 3H); ^{13}C NMR (126 MHz, $CDCl_3$) δ 162.97, 139.14, 135.01, 131.24, 122.54, 114.94, 55.58; GC-MS (m/z): 179.1 $[M]^+$.

(*E*)-1-Methoxy-2-(2-nitrovinyl)benzene (2e)

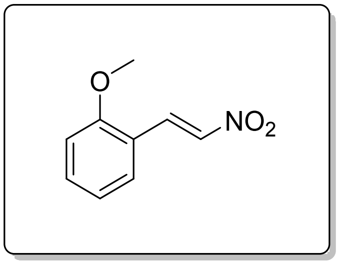

Light yellow solid; TLC (EtOAc:Hexane 2:8): R_f = 0.20; mp 84 - 86 ^{o}C; ^{1}H NMR (400 MHz, $CDCl_3$) δ 8.14–8.11 (d, J = 13.6 Hz, 1H), 7.89–7.87 (d, J = 13.6 Hz, 1H), 7.48–7.43 (m, 2H), 7.04–6.97 (m, 2H), 3.95 (s, 3H); ^{13}C NMR (126 MHz, $CDCl_3$) δ 159.53, 138.25, 135.60, 133.54, 132.56, 121.13, 119.09, 111.38, 55.67; GC-MS (m/z): 179.1 $[M]^+$.

(*E*)-1-(Tert-butyl)-4-(2-nitrovinyl)benzene (2f)

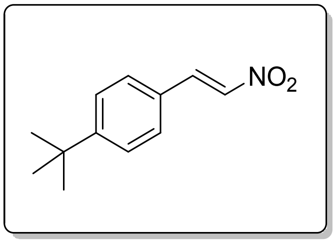

Light yellow liquid; TLC (EtOAc:Hexane 2:8): R_f = 0.20; ^{1}H NMR (500 MHz, $CDCl_3$) δ 8.02–7.99 (d, J = 13.7 Hz, 1H), 7.61-7.58 (d, J = 13.7 Hz, 1H), 7.51–7.49 (d, J = 8.9 Hz, 2 H),

7.49-7.47 (d, J = 8.9 Hz, 2H), 1.35 (s, 9H); ^{13}C NMR (126 MHz, $CDCl_3$) δ 156.21, 139.15, 136.42, 129.16, 127.26, 126.48, 35.19, 31.07; GC-MS (m/z): 205.1 $[M]^+$.

(*E*)-1,4-Dimethyl-2-(2-nitrovinyl)benzene (2g)

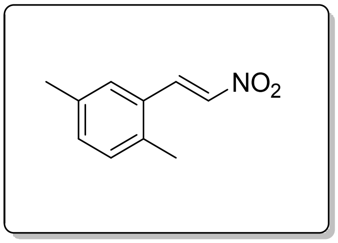

Light yellow liquid; TLC (EtOAc:Hexane 2:8): R_f = 0.20; ^{1}H NMR (400 MHz, $CDCl_3$) δ 8.29-8.26 (d, J = 13.6 Hz, 1H), 7.52-7.49 (d, J = 13.6 Hz, 1H), 7.32 (s, 1H), 7.21-7.15 (m, 2H), 2.43 (s, 3H), 2.34 (s, 3H); ^{13}C NMR (101 MHz, $CDCl_3$) δ 137.39, 136.93, 136.31 (d, J = 5.2 Hz), 132.88, 131.29, 128.71, 127.82, 20.85, 19.42; GC-MS (m/z): 177.07 $[M]^+$.

(*E*)-2,4-dimethyl-1-(2-nitrovinyl)benzene (2h)

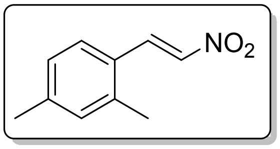

Light yellow solid; TLC (EtOAc:Hexane 1:9): R_f = 0.30; pale yellow liquid; ^{1}H NMR (400 MHz, $CDCl_3$) δ 8.23-8.19 (d, J = 13.6 Hz, 1H), 7.45-7.41 (d, J = 13.6 Hz, 1H), 7.36-7.34(d, J = 8 Hz, 1H), 7.03-6.98 (t, J = 8.4 Hz, 2H), 2.38 (s, 3H), 2.29 (s, 3H). ^{13}C NMR (126 MHz, $CDCl_3$) δ 142.84 (1C), 139.36 (1C), 136.85 (1C), 136.74 (1C), 132.22 (1C), 127.64 (1C), 127.41 (1C), 126.06 (1C), 21.55 (1C), 19.90 (1C); GC-MS (m/z): 177.1$[M]^+$.

(*E*)-2,4-Dimethoxy-1-(2-nitrovinyl)benzene (2i)

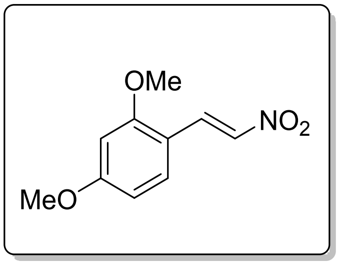

Light yellow solid; TLC (EtOAc:Hexane 2:8): R_f = 0.20; mp 106-108 ^oC; ^1H NMR (400 MHz, $CDCl_3$) δ 8.10-8.07 (d, J = 13.5 Hz, 1H), 7.84–7.81 (d, J = 13.5 Hz, 1H), 7.39–7.37 (d, J = 8.6 Hz, 1H), 6.57–6.54 (dd, J = 8.6, 2.3 Hz, 1H), 6.49–6.48 (d, J = 2.1 Hz, 1H), 3.93 (s, 3H), 3.87 (s, 3H); ^{13}C NMR (101 MHz, $CDCl_3$) δ 164.4, 161.2, 136.0, 135.7, 134.3, 112.4, 105.9, 98.6, 55.6; GC-MS (m/z): 209.1 $[M]^+$.

(*E*)-4-Methoxy-2-methyl-1-(2-nitrovinyl)benzene (2j)

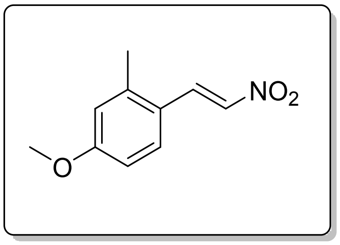

Light yellow solid; TLC (EtOAc:Hexane 2:8): R_f = 0.20; mp 221-223 ^oC; ^1H NMR (400 MHz, $CDCl_3$) δ 8.28–8.24 (d, J = 13.5 Hz, 1H), 7.51-7.46 (m, 2H), 6.79 (m, 2H), 3.85 (s, 3H), 2.47 (s, 3H); ^{13}C NMR (126 MHz, $CDCl_3$) δ 162.66, 141.89, 136.64, 135.40, 129.39, 121.38, 116.48, 112.75, 55.46, 20.32; GCMS (m/z): 193.2 $[M]^+$.

(*E*)-2-Fluoro-4-methoxy-1-(2-nitrovinyl)benzene (2k)

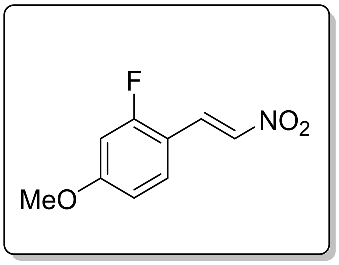

Light yellow solid; TLC (EtOAc:Hexane 2:8): R_f = 0.20; mp 221-223 ^oC; ^1H NMR (400 MHz, $CDCl_3$) δ 8.03–8.00 (d, J = 13.7 Hz, 1H), 7.68–7.64 (d, J = 13.7 Hz, 1H), 7.45–7.41 (t, J = 8.5 Hz, 1H), 6.80–6.77 (dd, J = 8.7, 2.4 Hz, 1H), 6.73–6.69 (dd, J = 12.6, 2.4 Hz, 1H), 3.87 (s, 3H); ^{13}C NMR (126 MHz, $CDCl_3$) δ 164.30-164.25 (d, J = 6.3 Hz), 164.20-162.21 (d, J = 250.74 Hz), 137.10-137.01 (d, J = 11.34 Hz), 132.84, 132.53-132.50 (d, J = 3.78 Hz), 111.41, 110.93-110.83 (d, J = 12.6 Hz), 102.57-102.37 (d, J = 25.2 Hz), 55.99; GC-MS (m/z): 197.1 $[M]^+$.

(*E*)-1,2,3-Trimethoxy-5-(2-nitrovinyl)benzene (2l)

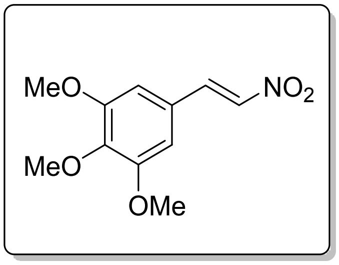

Light yellow solid; TLC (EtOAc:Hexane 2:8): R_f = 0.20; mp 120-123 °C; ^{1}H NMR (400 MHz, $CDCl_3$) δ 7.95–7.92 (d, *J* = 13.6 Hz, 1H), 7.57–7.54 (d, *J* = 13.6 Hz, 1H), 6.77 (s, 2H), 3.91 (d, *J* = 4.6 Hz, 9H); ^{13}C NMR (101 MHz, $CDCl_3$) δ 153.70, 141.82, 139.30, 136.38, 125.30, 106.51, 61.04, 56.29; GCMS (m/z): 239.1 $[M]^+$.

(*E*)-1-Fluoro-4-(2-nitrovinyl)benzene (2m)

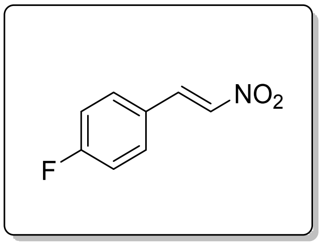

Light yellow solid; TLC (EtOAc:Hexane 2:8): R_f = 0.20; mp 98-100 °C; ^{1}H NMR (400 MHz, $CDCl_3$) *δ* 8.09-7.97 (d, *J* = 13.7 Hz, 1H), 7.59–7.54 (m, 3H), 7.18-7.14 (d, *J* = 8.5 Hz, 2H); ^{19}F NMR (376 MHz, $CDCl_3$) *δ* -105.76 (qd, *J* = 8.3, 5.3 Hz); ^{13}C NMR (126 MHz, $CDCl_3$) *δ* 165.96-163.94 (d, *J*= 254.52), 137.95, 136.85, 131.41–131.34 (d, *J* = 8.82 Hz), 126.33, 116.89; GC-MS (m/z): 167.1 $[M]^+$.

(*E*)-1-Chloro-4-(2-nitrovinyl)benzene (2n)

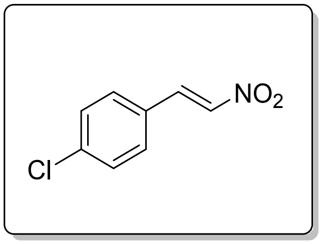

Light yellow solid; TLC (EtOAc:Hexane 2:8): R_f = 0.20; mp 112-114 °C; ^{1}H NMR (400 MHz, $CDCl_3$) *δ* 7.99-7.95 (d, *J* = 13.7 Hz, 1H), 7.58-7.55 (d, *J* = 13.7 Hz, 1H), 7.51-7.49 (d, *J* = 8.5 Hz, 2H), 7.45-7.43 (d, *J* = 8.5 Hz, 2H); ^{13}C NMR (101 MHz, $CDCl_3$) *δ* 128.65, 129.87, 130.42, 137.53, 137.85, 138.43, 77.51, 77.20, 76.88; GC-MS (m/z): 183.1 $[M]^+$.

(*E*)-1-Bromo-4-(2-nitrovinyl)benzene (2o)

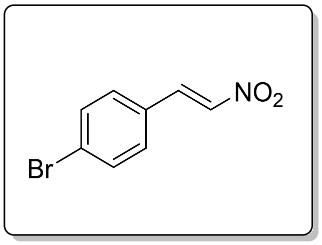

Light yellow solid; TLC (EtOAc:Hexane 2:8): R_f = 0.20; mp 141-144 °C; ^{1}H NMR (400 MHz, $CDCl_3$) δ 7.89–7.86 (d, J = 13.7 Hz, 1H), 7.54–7.49 (m, 3H), 7.36–7.33 (d, J = 8.4 Hz, 2H); ^{13}C NMR (126 MHz, $CDCl_3$) δ 137.86, 137.47, 132.78, 130.44, 128.94, 126.84; GC-MS (m/z): 226.9 $[M]^+$.

(*E*)-1-Bromo-3-(2-nitrovinyl)benzene (2p)

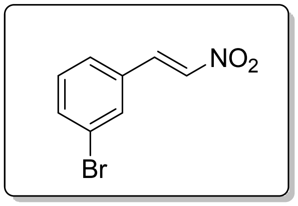

Light yellow solid; TLC (EtOAc:Hexane 2:8): R_f = 0.20; mp 59-61 °C; ^{1}H NMR (400 MHz, $CDCl_3$) δ 7.93 (d, J = 13.7 Hz, 1H), 7.70 (s, 1H), 7.63 (d, J = 8.0 Hz, 1H), 7.56 (d, J = 13.7 Hz, 1H), 7.48 (d, J = 7.8 Hz, 1H), 7.34 (t, J = 7.9 Hz, 1H); ^{13}C NMR (126 MHz, $CDCl_3$) δ 138.07, 137.43, 134.92, 132.07, 131.71, 130.91, 127.72, 123.45; GC-MS (m/z): 228.9 $[M]^+$.

(*E*)-1-(2-Nitrovinyl)-4-(trifluoromethyl)benzene (2q)

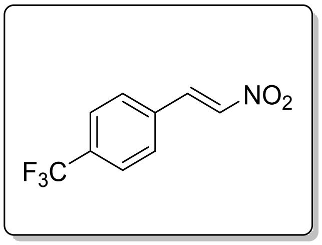

Light yellow solid; TLC (EtOAc:Hexane 2:8): R_f = 0.20; mp 90-92 ^{o}C; ^{1}H NMR (400 MHz, $CDCl_3$) δ 8.05-8.01 (d, J = 13.7 Hz, 1H), 7.74-7.72 (d, J = 8.4 Hz, 2H), 7.70-7.68 (d, J = 8.4 Hz, 2H), 7.66-7.62 (d, J = 13.7 Hz, 1H); ^{13}C NMR (126 MHz, $CDCl_3$) δ 138.86, 137.20, 133.78-132.99 (q, J = 32.76, 30.24 Hz), 129.32, 126.31- 126.31 (q, J = 16.38, 3.78 Hz), 124.59, 122.43; GC-MS (m/z): 217.1 $[M]^+$.

(*E*)-1-(2-Nitrovinyl)-4-(trifluoromethoxy)benzene (2r)

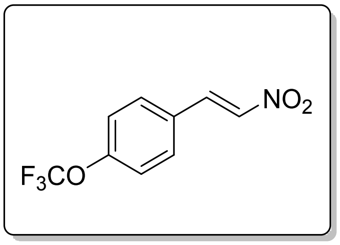

Light yellow solid; TLC (EtOAc:Hexane 2:8): R_f = 0.20; mp 221-223 °C; ^{1}H NMR (400 MHz, $CDCl_3$) δ 8.02-7.98 (d, J = 13.7 Hz, 1H), 7.62-7.60 (d, J = 8.7 Hz, 2H), 7.59-7.56 (d, J = 13.7 Hz, 1H), 7.32-7.30 (d, J = 8.7 Hz, 2H); ^{19}F NMR (376 MHz, $CDCl_3$) δ -57.72; ^{13}C NMR (101 MHz, $CDCl_3$) δ 151.71-151.70(d, J = 1.01 Hz), 137.68, 137.33, 130.75, 128.56, 121.45, 119.00; GC-MS (m/z): 233.1 $[M]^+$.

(*E*)-1-(2-nitrovinyl)-3,5-bis(trifluoromethyl)benzene (2s)

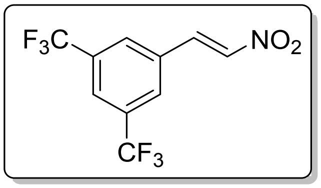

Pale yellow solid; TLC (EtOAc:Hexane 1:9): R_f = 0.30; mp 92-93°C; ^{1}H NMR (400 MHz, $CDCl_3$) δ 8.08-8.05 (d, J = 14 Hz, 1H), 7.99 (s, 3H), 7.70-7.66 (d, J = 13.6 Hz, 1H). ^{13}C NMR (126 MHz, $CDCl_3$) δ 139.81 (s, 1C), 135.46 (s, 1C), 133.49-132.72(q, J= 30.24, 64.26Hz. 1C), 132.25 (s, 1C), 128.64-128.61 (d, J= 3.78Hz 1C), 125.17-125.06 (m, 1C), 123.75 (s, 1C), 121.58 (s, 1C); GC-MS (m/z): 285.0$[M]^+$.

(*E*)-4-(2-Nitrovinyl)benzonitrile (2t)

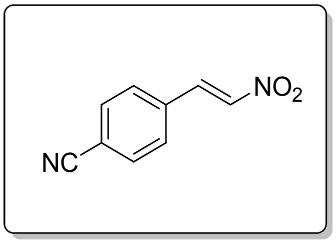

Light yellow solid; TLC (EtOAc:Hexane 2:8): R_f = 0.20; mp 183-186 °C; ^{1}H NMR (400 MHz, $CDCl_3$) δ 8.00 (d, J = 13.8 Hz, 1H), 7.76 (d, J = 8.2 Hz, 2H), 7.67 (d, J = 8.2 Hz, 2H), 7.62 (d, J = 13.8 Hz, 1H); ^{13}C NMR (126 MHz, $CDCl_3$) δ 139.48, 136.67, 134.38, 133.06, 129.47, 117.89, 115.18; GC-MS (m/z): 174.1 $[M]^+$.

(*E*)-4-(2-nitrovinyl)phenyl acetate (2u)

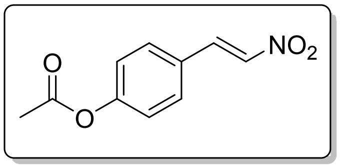

White solid; TLC (EtOAc:Hexane 1:9): R_f = 0.30; mp 176-178°C; ^{1}H NMR (400 MHz, $CDCl_3$) δ 8.01 (d, J = 8 Hz, 1H), 7.59 (dd, J = 12, 12 Hz, 3H), 7.22 (d, J = 8 Hz, 2H), 2.33 (s, 3H). ^{13}C NMR (101 MHz, $CDCl_3$) δ 168.87 (1C), 153.54 (1C), 138.00 (1C), 137.17 (1C), 130.42 (2C), 127.69(1C), 122.78 (2C), 21.14(1C); GC-MS (m/z): 207.0$[M]^+$.

(*E*)-2-(2-Nitrovinyl)naphthalene (2v)

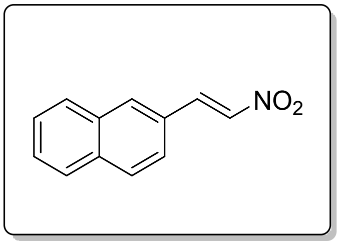

Light yellow solid; TLC (EtOAc:Hexane 2:8): R_f = 0.20; mp 128-130 °C; ^{1}H NMR (400 MHz, $CDCl_3$) δ 8.16-8.13 (d, J = 13.6 Hz, 1H), 8.00 (s, 1H), 7.90 –7.85 (m, 3H), 7.70-7.67 (d, J = 13.6 Hz, 1H), 7.61–7.54 (m, 3H); ^{13}C NMR (126 MHz, $CDCl_3$) δ 139.33, 137.13, 134.93, 133.15, 132.41, 129.40, 128.88, 128.45, 127.99, 127.54, 127.33, 123.32; GC-MS (m/z): 199.1 $[M]^+$.

(*E*)-4-(2-nitrovinyl)-1,1'-biphenyl (2w)

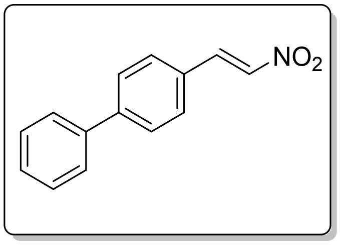

Light yellow solid; TLC (EtOAc:Hexane 2:8): R_f = 0.40; mp 190-192 °C; ^{1}H NMR (400 MHz, $CDCl_3$) δ 7.47-7.44 (m, 5H), 7.41-7.38 (m, 2H), 7.30 –7.28 (m, 2H), 7.24-7.23 (m, 2H); ^{13}C NMR (126 MHz, $CDCl_3$) δ 150.59, 137.10, 135.56, 134.41, 130.97, 129.37, 128.97, 128.93, 128.84, 128.55;.

(*E/Z*)-1-fluoro-4-(1-nitroprop-1-en-2-yl)benzene (2x)

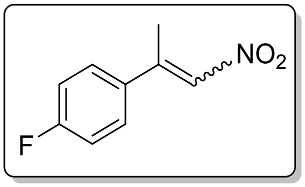

Yellow oil; TLC (EtOAc:Hexane 1:9): R_f = 0.30; ^{1}H NMR (400 MHz, $CDCl_3$) δ 7.41 – 7.36 (m, 2H), 7.22-7.21 (d, *J* = 1.2 Hz, 1H), 7.10 – 7.04 (m, 2H), 2.57-2.56 (d, *J* = 1.4 Hz, 3H); ^{13}C NMR (126 MHz, CDCl3) δ 165.23 (1C), 163.16 (1C), 148.88 (1C), 136.29 (1C), 134.28 (1C), 128.91-128.84 (d, *J* = 8.75 Hz, 1C), 116.33 (1C), 116.15 (1C), 18.68 (1C).

(*E/Z*)-(1-nitroprop-1-en-2-yl)benzene (2y)

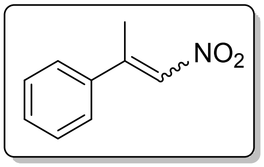

Light yellow liquid; TLC (EtOAc:Hexane 1:9): R_f = 0.30; ^{1}H NMR (400 MHz, $CDCl_3$) δ 7.38 (s, 5H), 7.24-7.23 (dd, *J* = 2.7,1.3 Hz, 1H), 2.579-2.575 (d, *J* = 1.6 Hz, 3H). ^{13}C NMR (101

M(1C)Hz, $CDCl_3$) δ 149.94 (1C), 138.35, 136.38 (1C), 130.38 (1C), 129.06 (2C), 126.86 (2C), 18.60(1C); GC-MS (m/z): 163.1$[M]^+$

(*E*)-2-(2-Nitrovinyl)furan (4a)

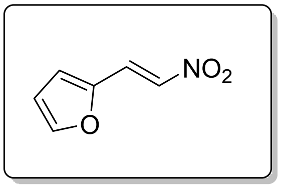

Light yellow solid; TLC (EtOAc:Hexane 2:8): R_f = 0.20; mp 77-79 °C; ^{1}H NMR (500 MHz, $CDCl_3$) *δ* 7.80-7.77 (d, *J* = 13.2 Hz, 1H), 7.61 (s, 1H), 7.54-7.52 (d, *J* = 13.2 Hz, 1H), 6.92-6.91 (d, *J* = 3.5 Hz, 1H), 6.60-6.59 (dd, *J* = 3.5, 1.8 Hz, 1H); ^{13}C NMR (126 MHz, $CDCl_3$) *δ* 146.93, 146.62, 134.84, 125.53, 120.19, 113.43; GC-MS (m/z): 139.1 $[M]^+$.

(*E*)-1-nitronon-1-ene (4b)

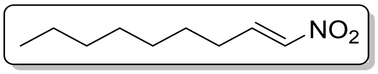

Yellow liquid; TLC (EtOAc:Hexane 1:9): R_f = 0.40; ^{1}H NMR (400 MHz, $CDCl_3$) δ 7.31 – 7.24 (m, 1H), 6.70-6.96 (dt, *J* = 13.4, 1.5 Hz, 1H), 2.29 – 2.23 (m, 2H), 1.55 – 1.47 (m, 2H), 1.34 – 1.28 (m, 9H), 0.90-0.86 (t, *J* = 6.8 Hz, 3H). ^{13}C NMR (101 MHz, $CDCl_3$) δ 142.83 (1C), 139.58 (1C), 31.65 (1C), 29.05 (1C), 28.92 (1C), 28.45 (1C), 27.74 (1C), 22.59 (1C), 14.03 (1C);

(*E*)-1-nitrohex-1-ene (4c)

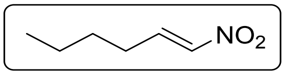

Yellow liquid; TLC (EtOAc:Hexane 1:9): R_f = 0.40; ^{1}H NMR (400 MHz, $CDCl_3$) δ 7.31 – 7.24 (m, 1H), 6.99-6.96 (d, *J* = 13.2 Hz, 1H), 2.30 – 2.24 (m, 2H), 1.54 – 1.46 (m, 2H), 1.42 – 1.35 (m, 2H), 0.95-0.91 (t, *J* = 7.6 Hz, 3H). ^{13}C NMR (101 MHz, $CDCl_3$) δ 142.75 (1C), 139.58 (1C), 29.77 (1C), 28.10 (1C), 22.15 (1C), 13.63 (1C); ESI-MS (m/z) : 130.12$(M+H)^+$

(*E/Z*)-2-methyl-1-nitronon-1-ene (4d)

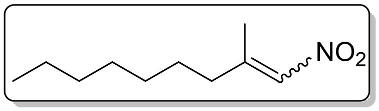

Yellow liquid; TLC (EtOAc:Hexane 1:15): R_f = 0.50; ^{1}H NMR (400 MHz, $CDCl_3$) δ 6.89 – 6.88 (m, 1H), 2.58-2.54 (t, *J* = 8 Hz, 1H), 2.17 – 2.16 (d, *J* = 1.2 Hz, 3H), 2.13-2.09 (m, 2H), 1.86 – 1.85 (d, *J* = 1.6 Hz, 1H), 1.48-1.41(m, 3H), 1.23-1.14(m, 12H), 0.83-0.80 (t, *J* = 6.4 Hz, 3H). ^{13}C NMR (101 MHz, $CDCl_3$) δ 153.62 (1C), 135.16 (1C), 38.06 (1C), 31.66 (1C), 29.07 (1C), 28.97 (1C), 27.11 (1C), 22.59 (1C), 18.57 (1C), 14.06 (1C); HRMS (TOF MS ES-) : m/z 184.1332 calculated for $C_{10}H_{19}NO_2$ - H^+ (184.1338).

Spectras (^{1}H NMR, ^{13}C NMR, DEPT and GC-MS) of synthesized products

^{1}H NMR of (*E*)-(2-Nitrovinyl)benzene (2a)[1]

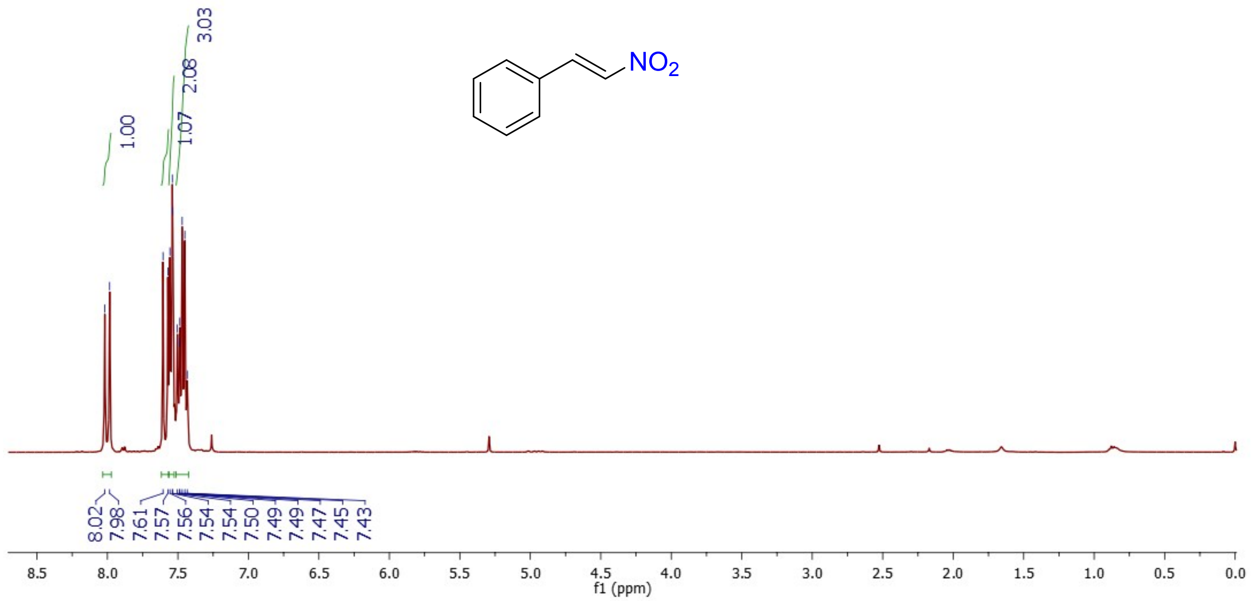

^{13}C NMR of (*E*)-(2-Nitrovinyl)benzene (2a)

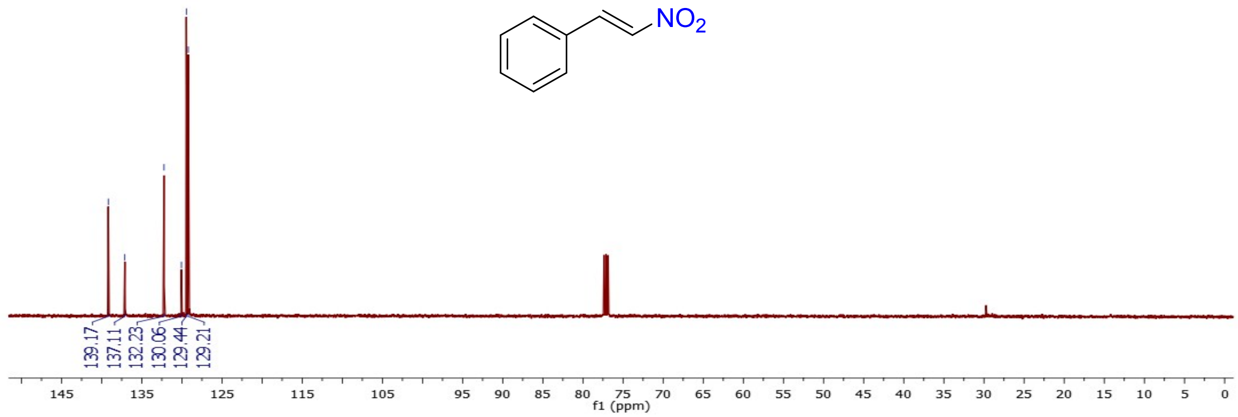

DEPT of (*E*)-(2-Nitrovinyl)benzene (2a)

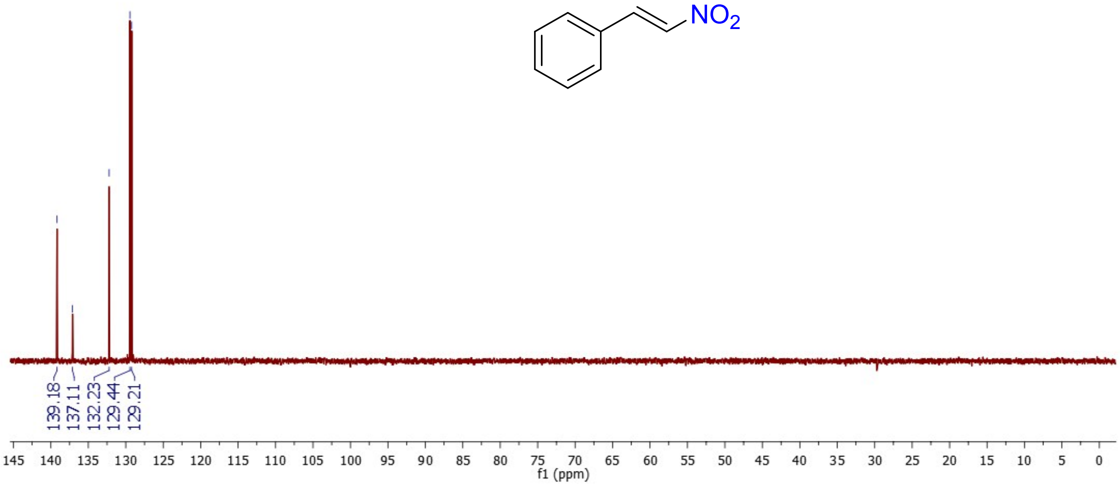

GC-MS of (*E*)-(2-Nitrovinyl)benzene (2a)

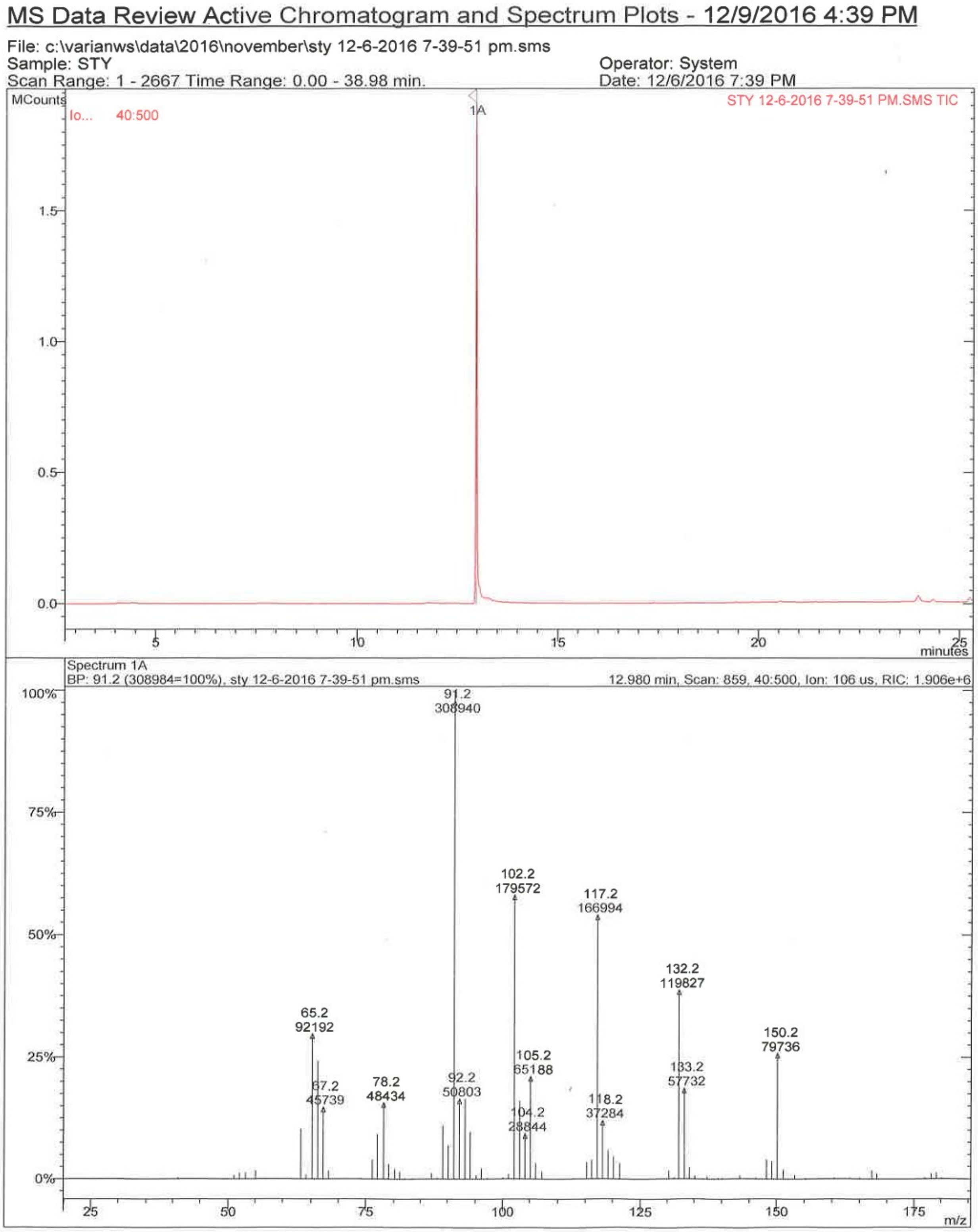

^{1}H NMR of (*E*)-4-Methyl-2-(2-nitrovinyl)benzene (2b)[1]

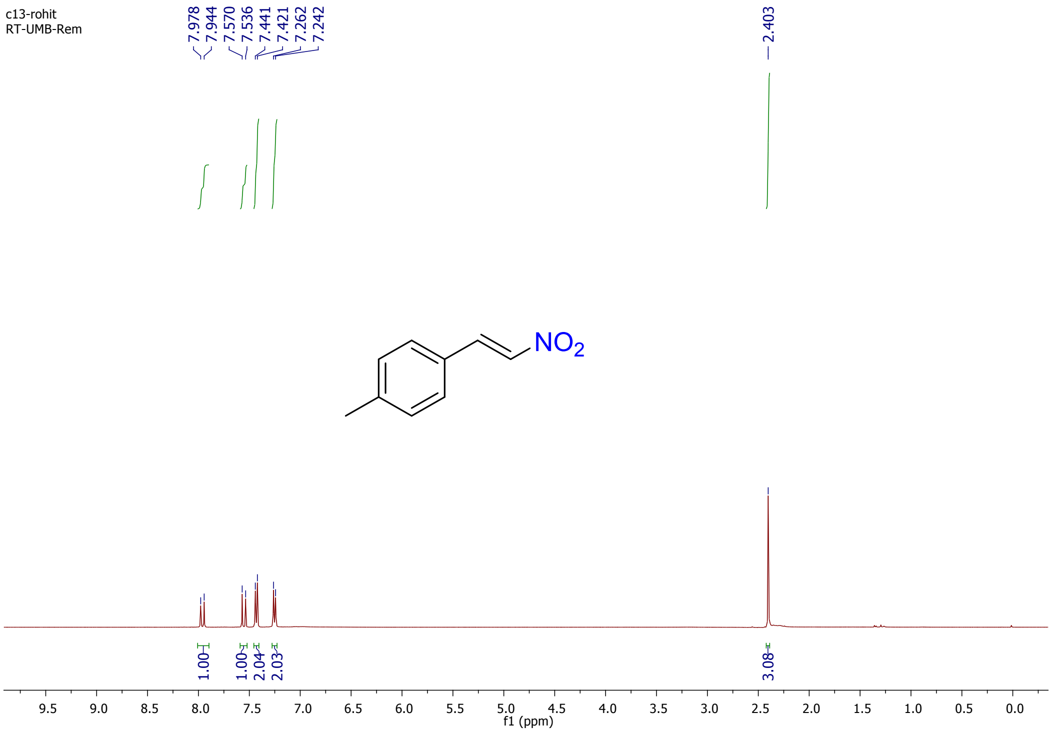

^{13}C NMR of (*E*)-4-Methyl-2-(2-nitrovinyl)benzene (2b)

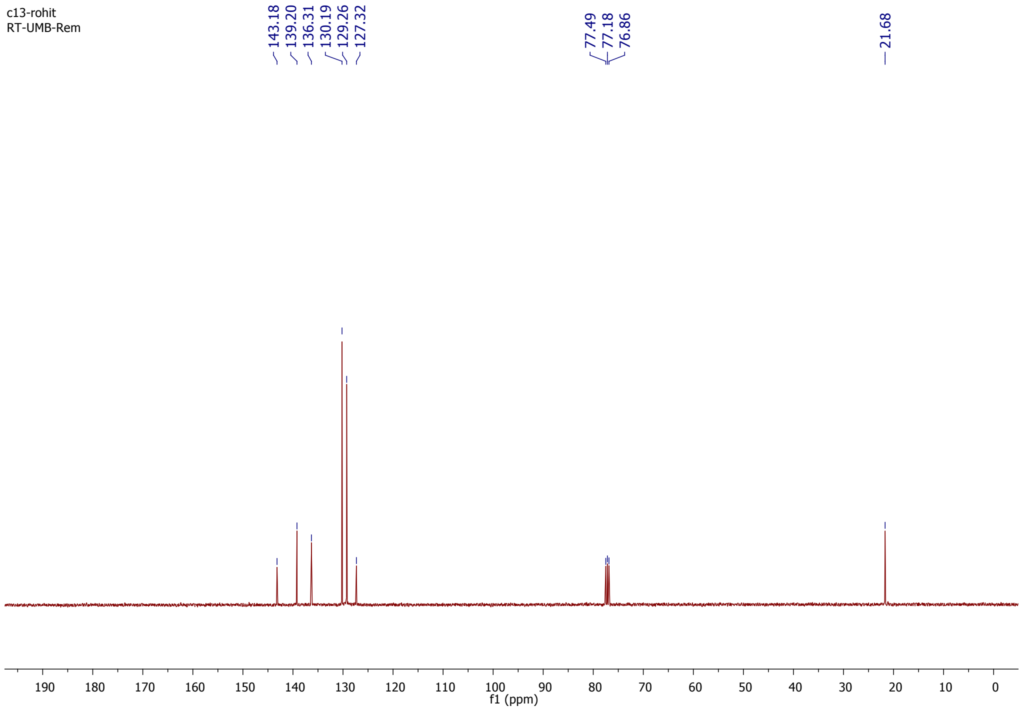

DEPT NMR of (*E*)-4-Methyl-2-(2-nitrovinyl)benzene (2b)

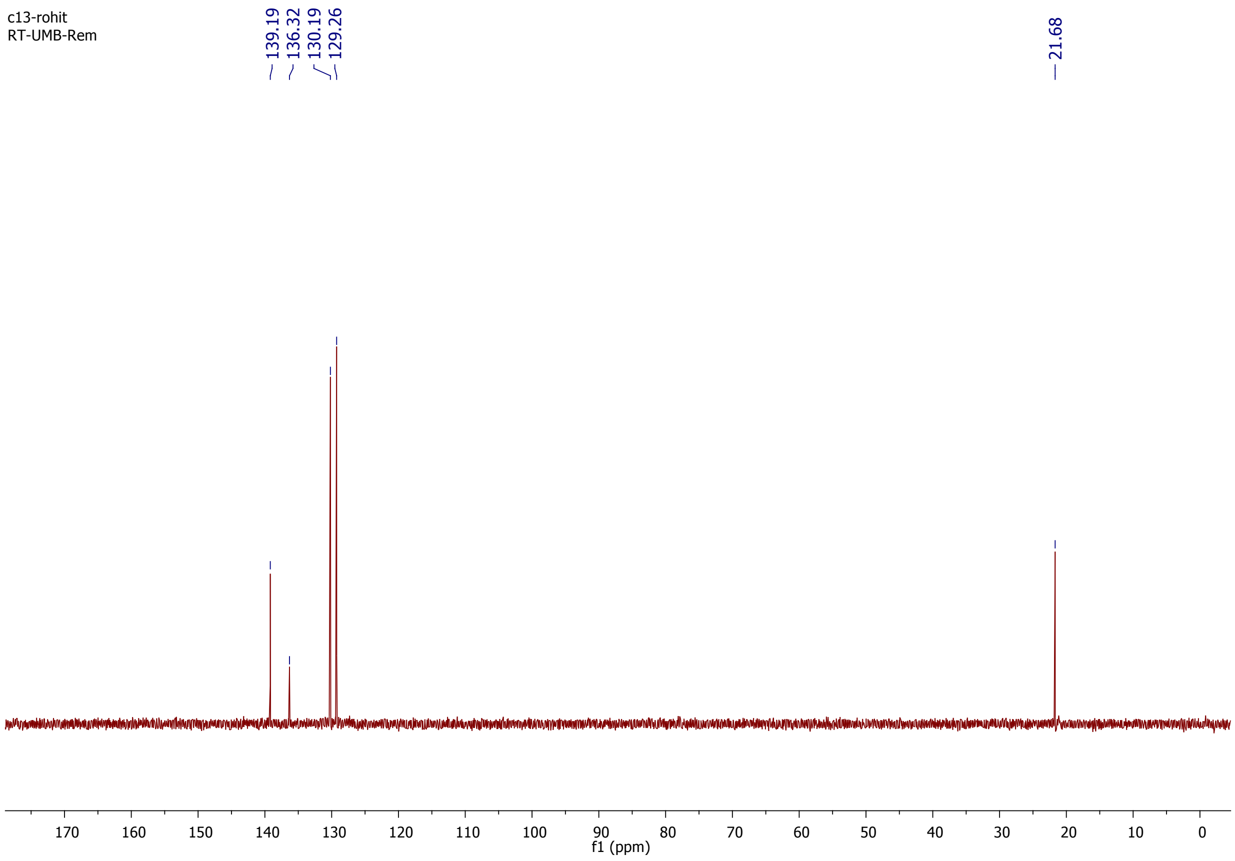

GC-MS of (*E*)-4-Methyl-2-(2-nitrovinyl)benzene (2b)

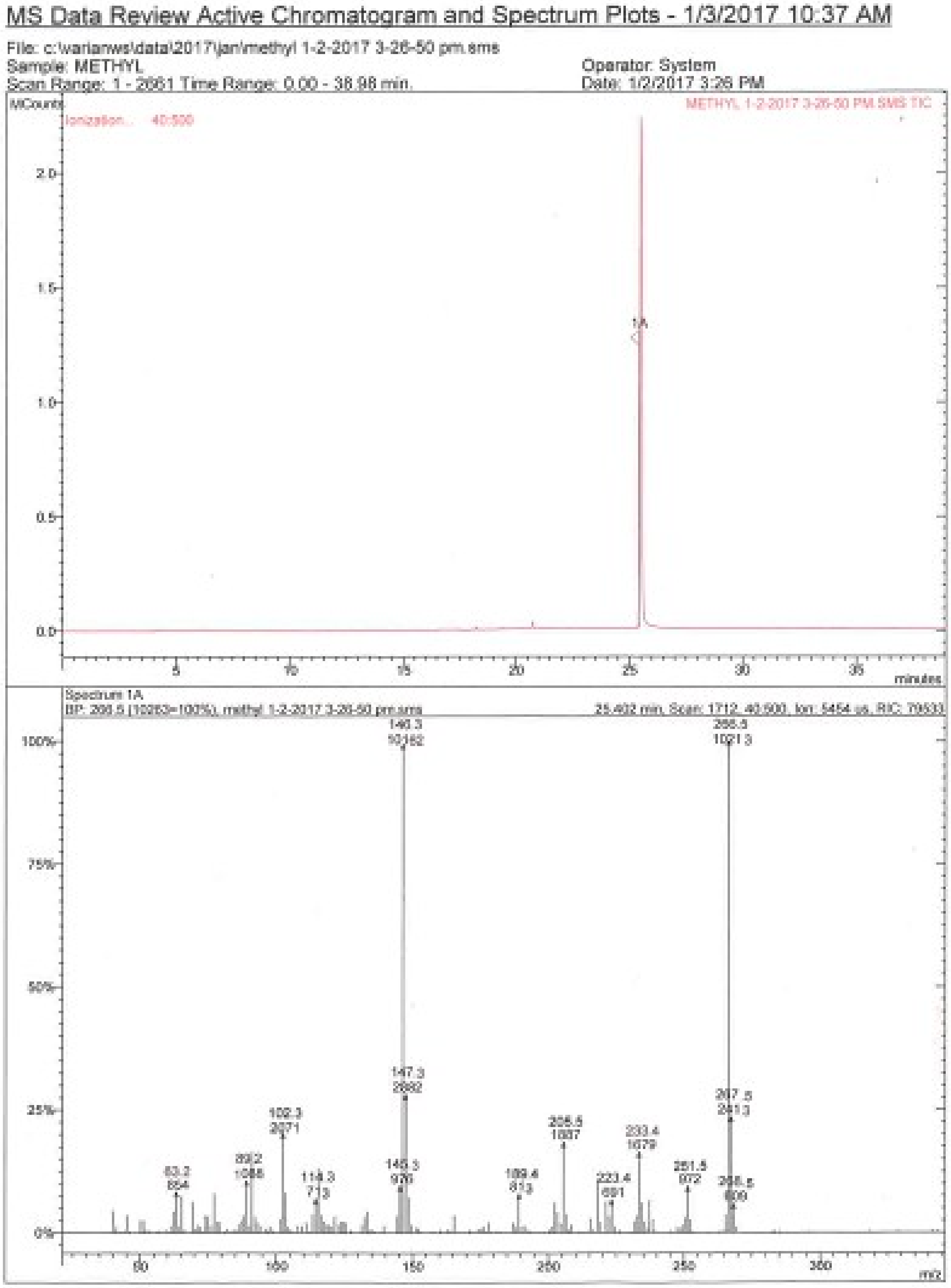

^{1}H NMR of (*E*)-1-Methyl-2-(2-nitrovinyl)benzene (2c)[2]

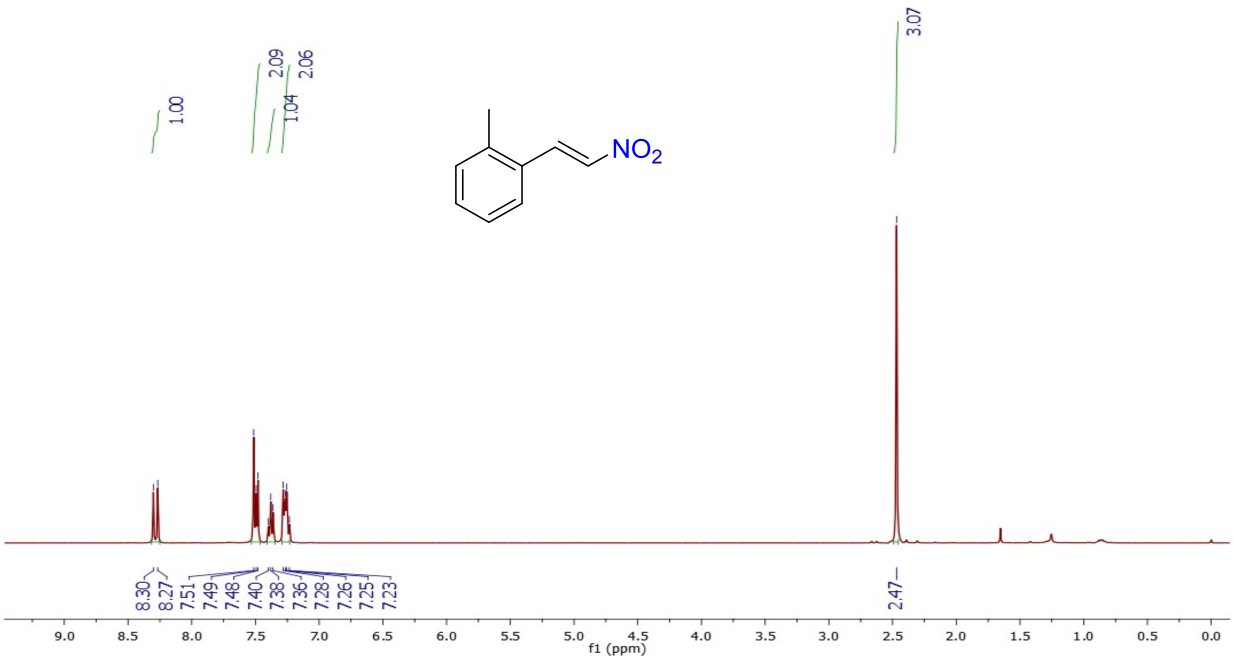

^{13}C NMR of (*E*)-1-Methyl-2-(2-nitrovinyl)benzene (2c)

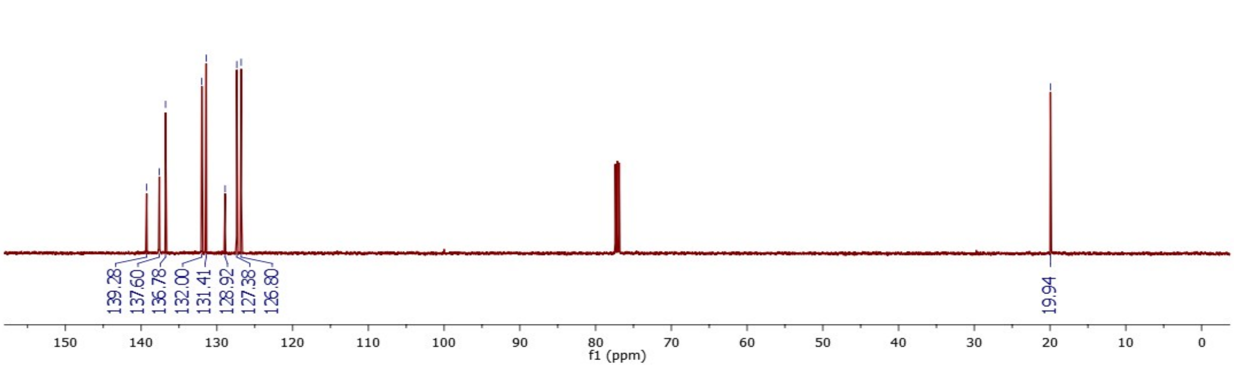

DEPT of (*E*)-1-Methyl-2-(2-nitrovinyl)benzene (2c)

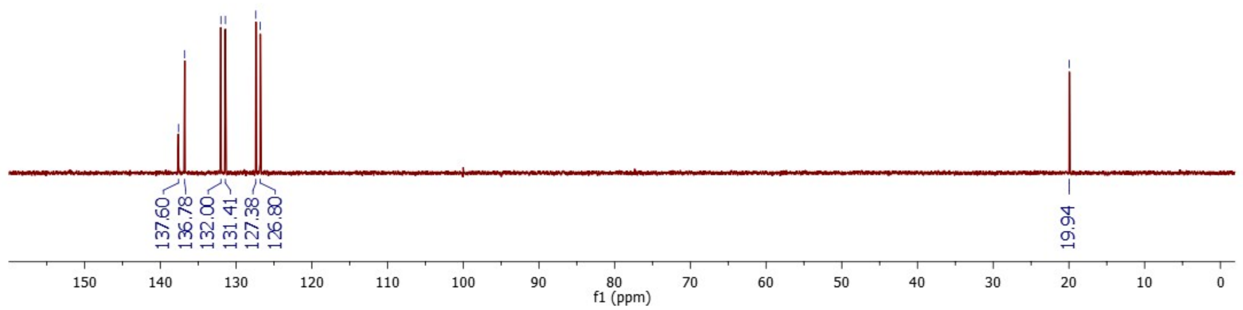

GC-MS of (*E*)-1-Methyl-2-(2-nitrovinyl)benzene (2c)

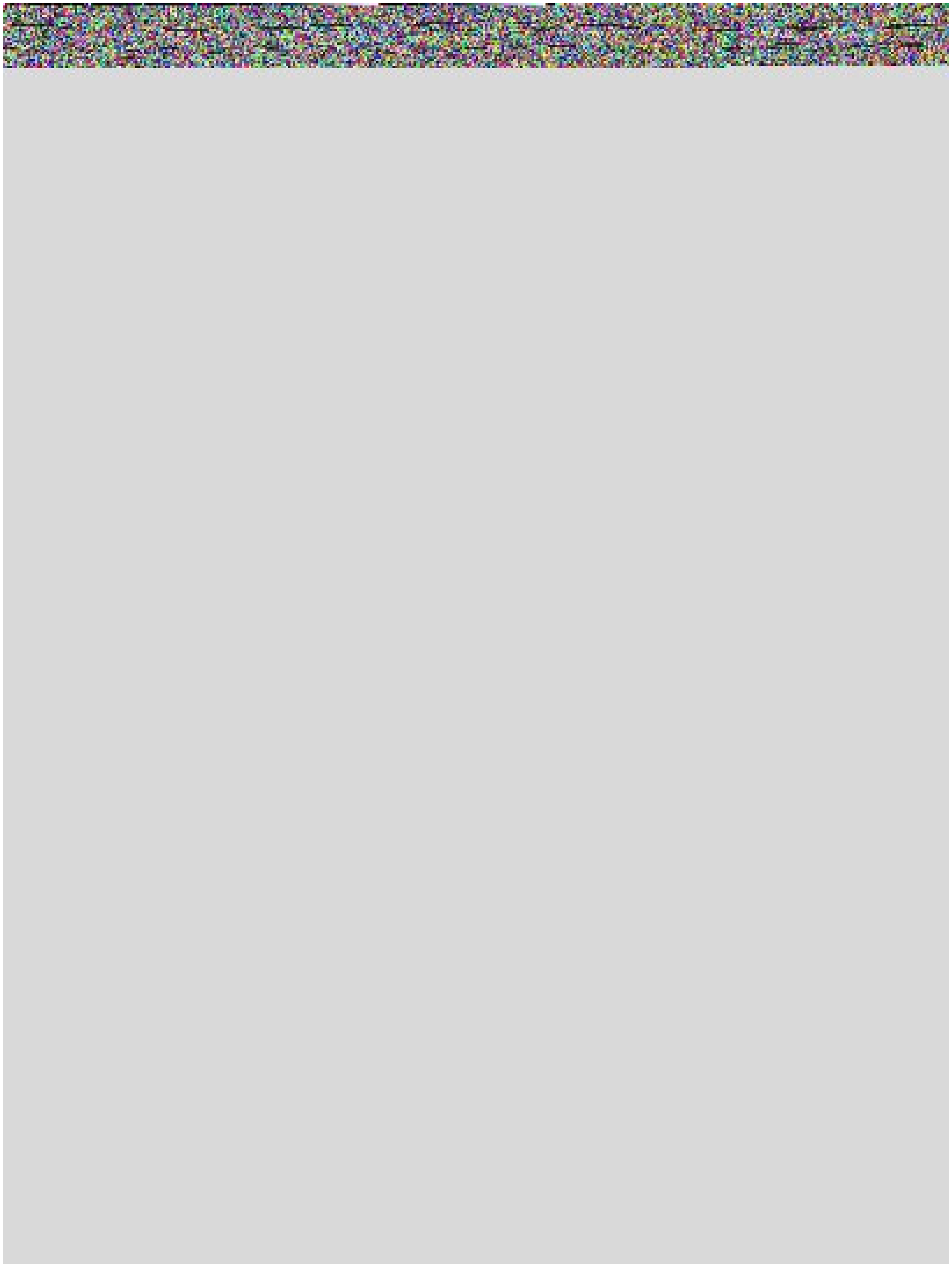

^{1}H NMR of (E)-1-methoxy-4-(2-nitrovinyl)benzene (2d)[1]

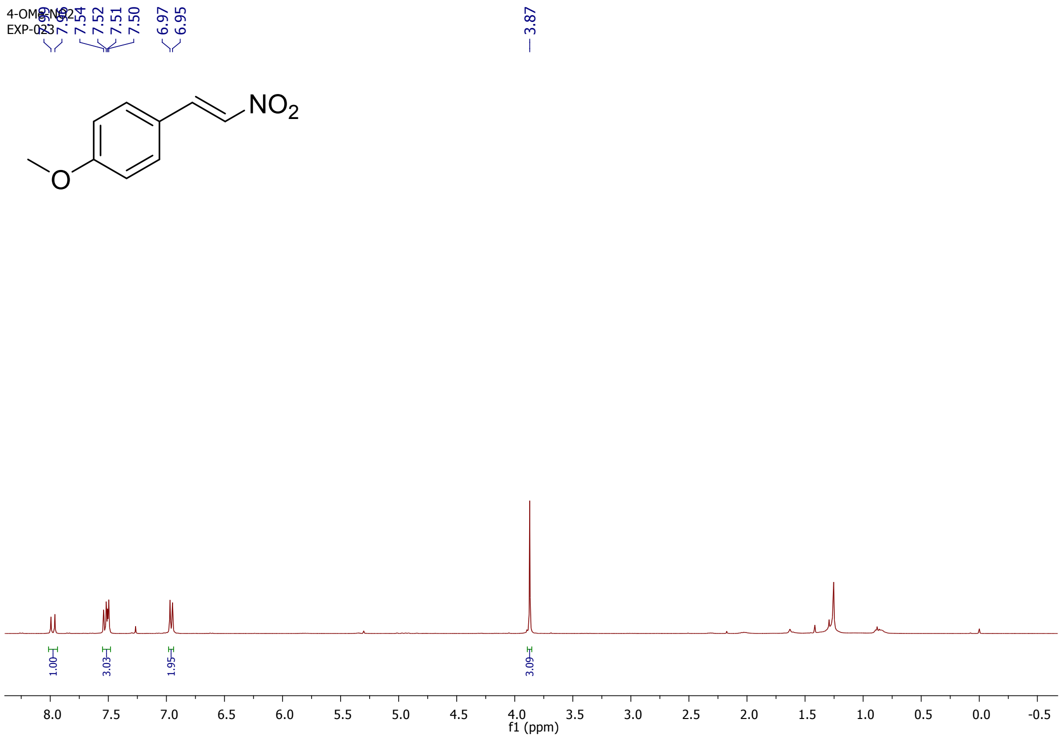

^{13}C NMR of (E)-1-methoxy-4-(2-nitrovinyl)benzene (2d)

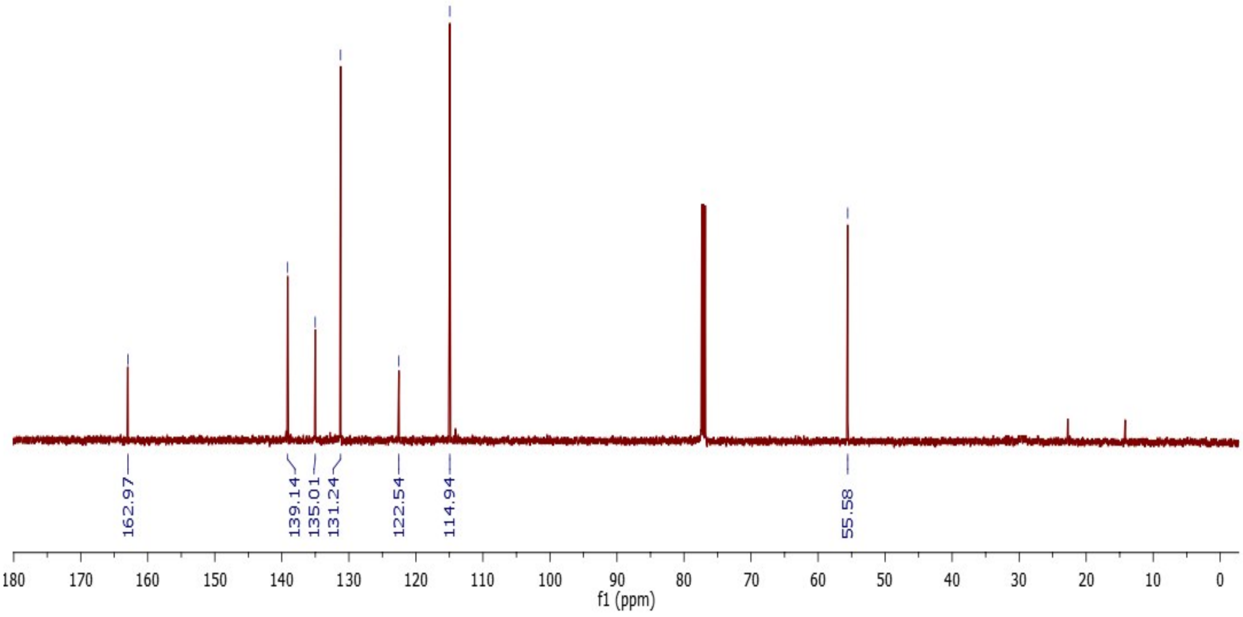

GC-MS of (E)-1-methoxy-4-(2-nitrovinyl)benzene (2d)

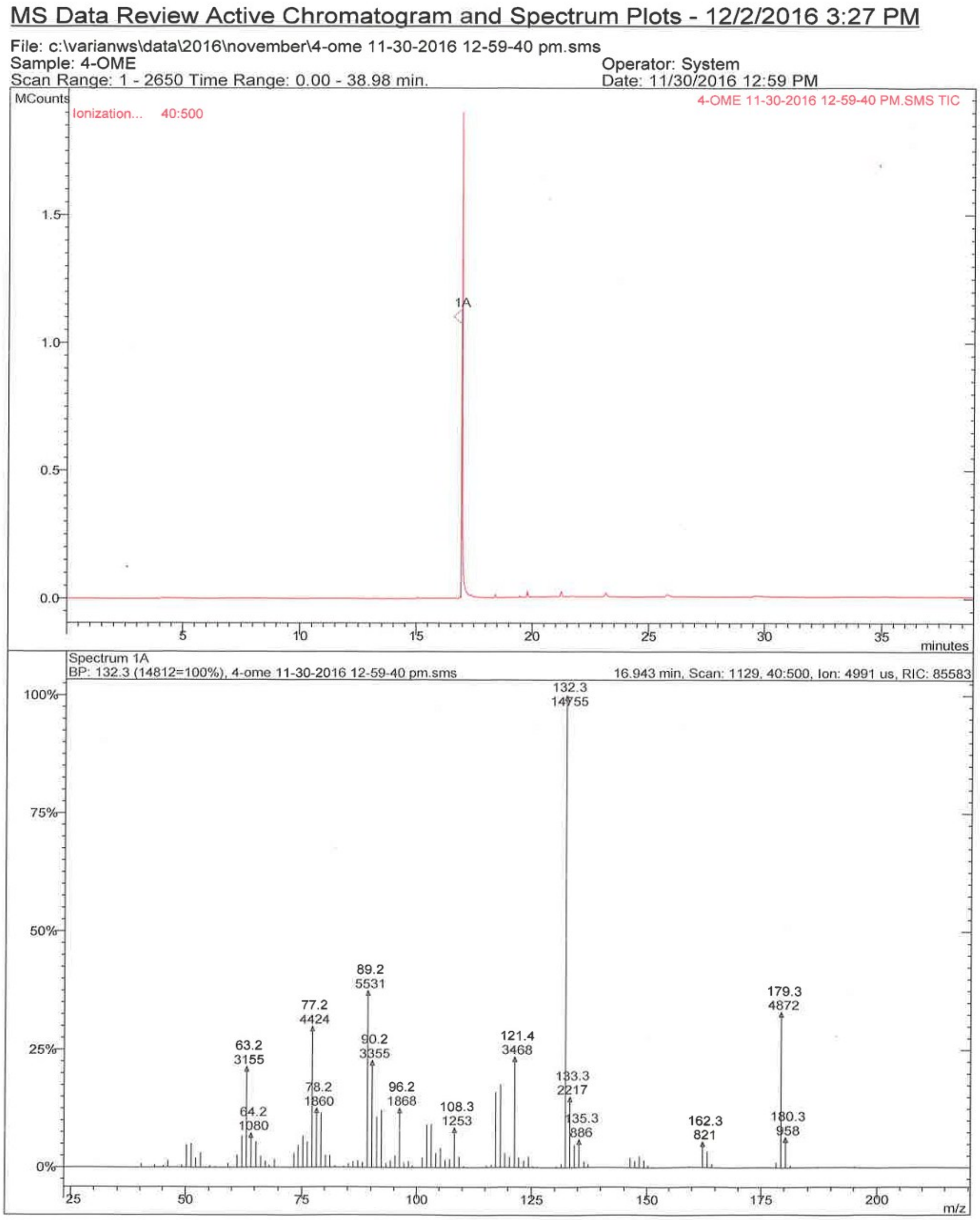

^{1}H NMR of (E)-1-methoxy-2-(2-nitrovinyl)benzene (2e)[3]

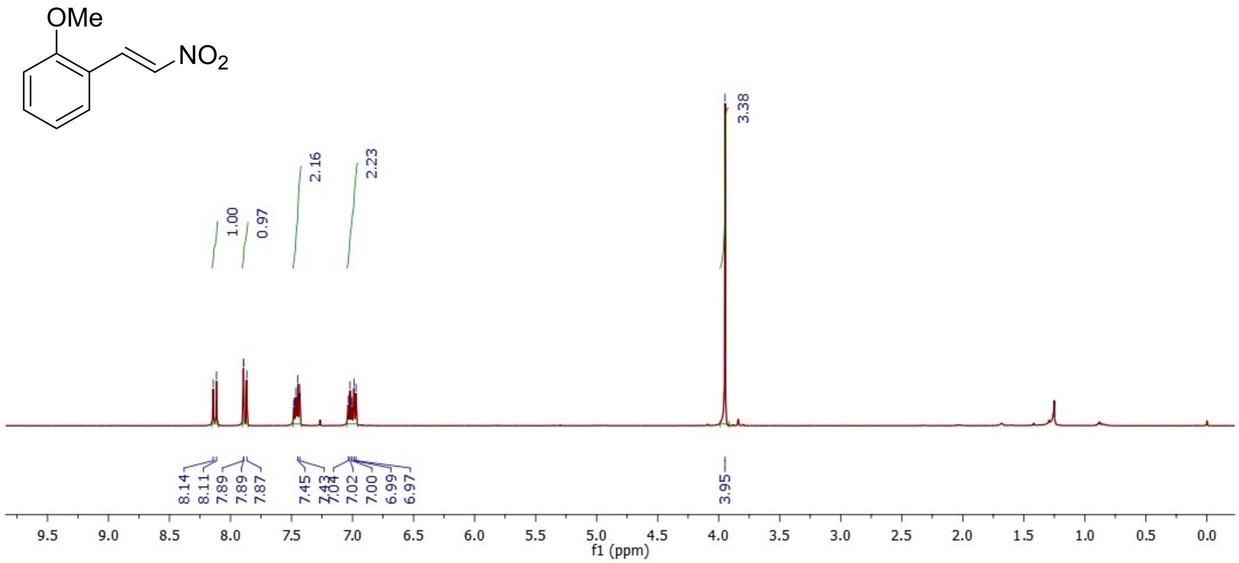

^{13}C NMR of (E)-1-methoxy-2-(2-nitrovinyl)benzene (2e)

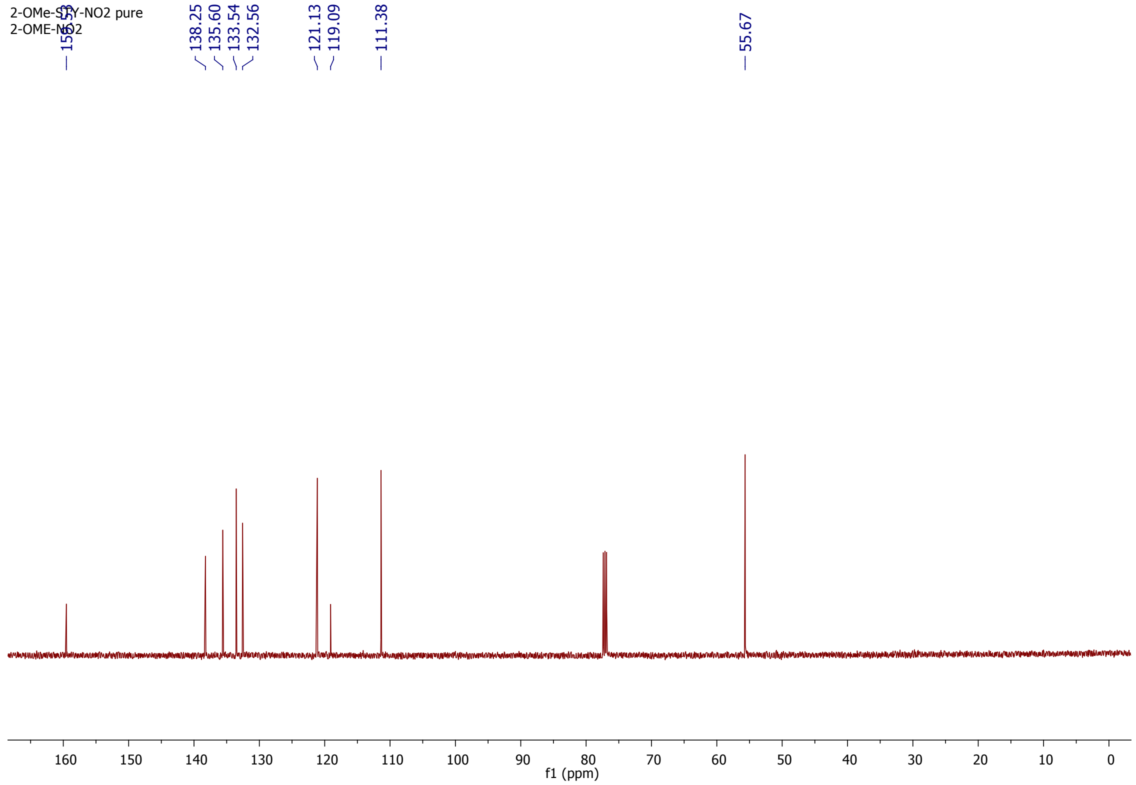

DEPT NMR of (E)-1-methoxy-2-(2-nitrovinyl)benzene (2e)

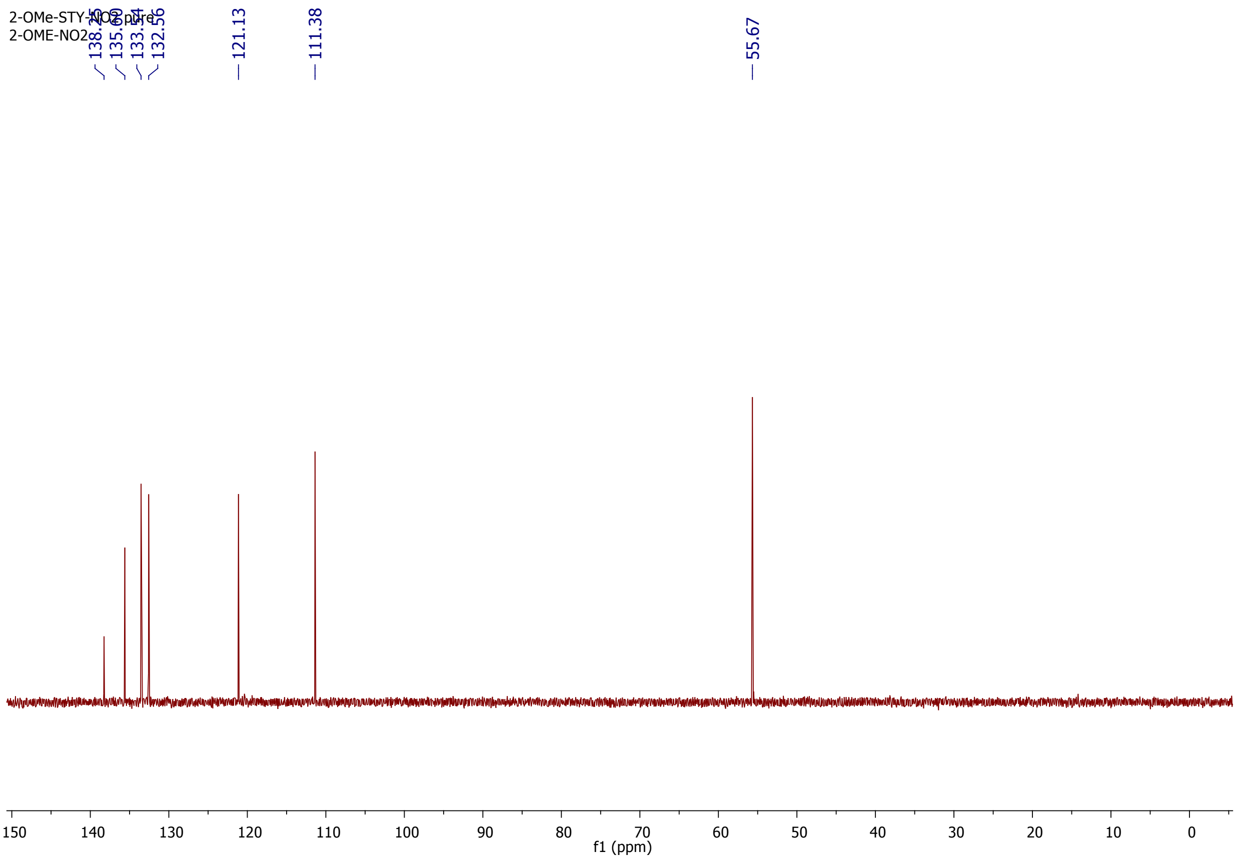

GC-MS of (E)-1-methoxy-2-(2-nitrovinyl)benzene (2e)

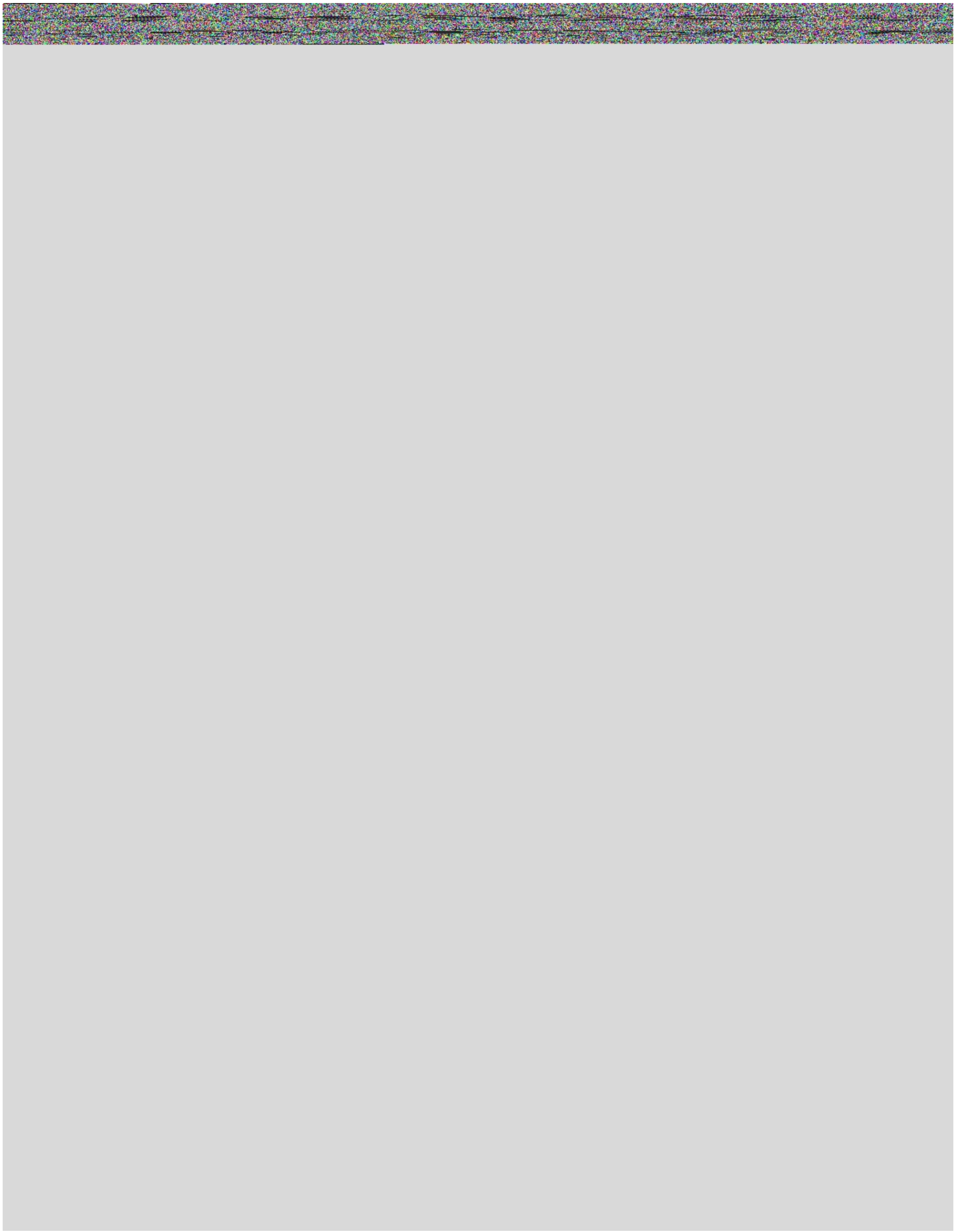

^{1}H NMR of (E)-1-(tert-butyl)-4-(2-nitrovinyl)benzene (2f)[4]

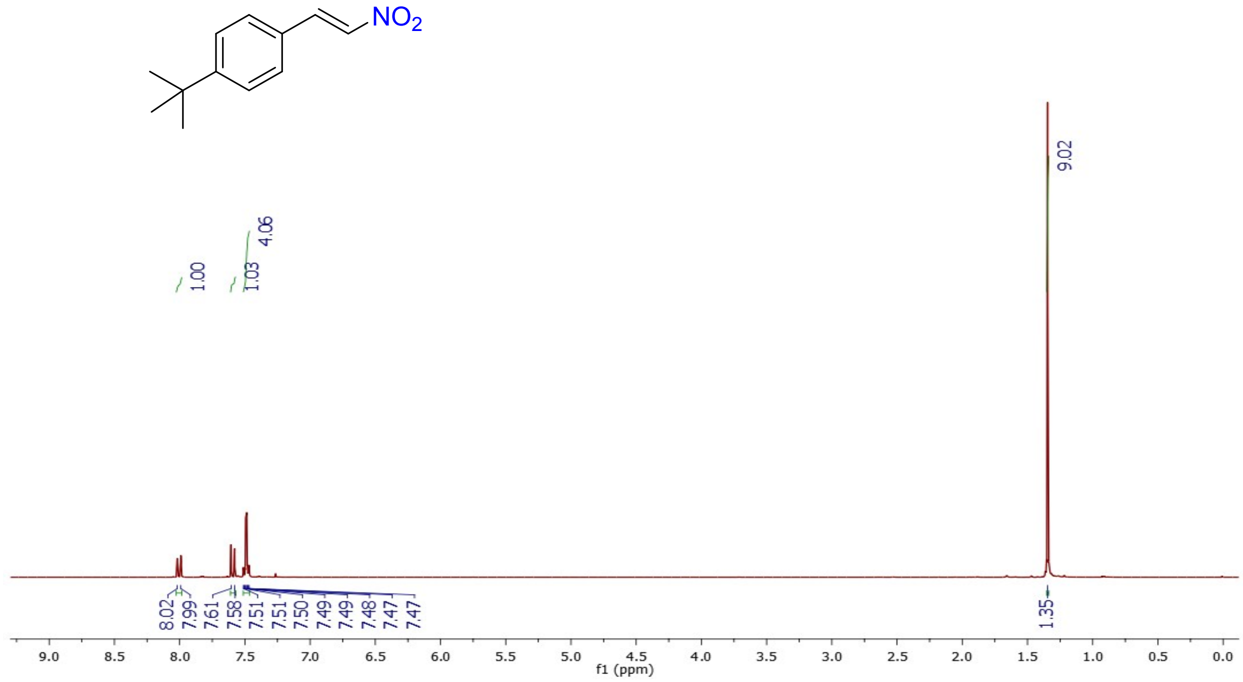

^{13}C NMR of (E)-1-(tert-butyl)-4-(2-nitrovinyl)benzene (2f)

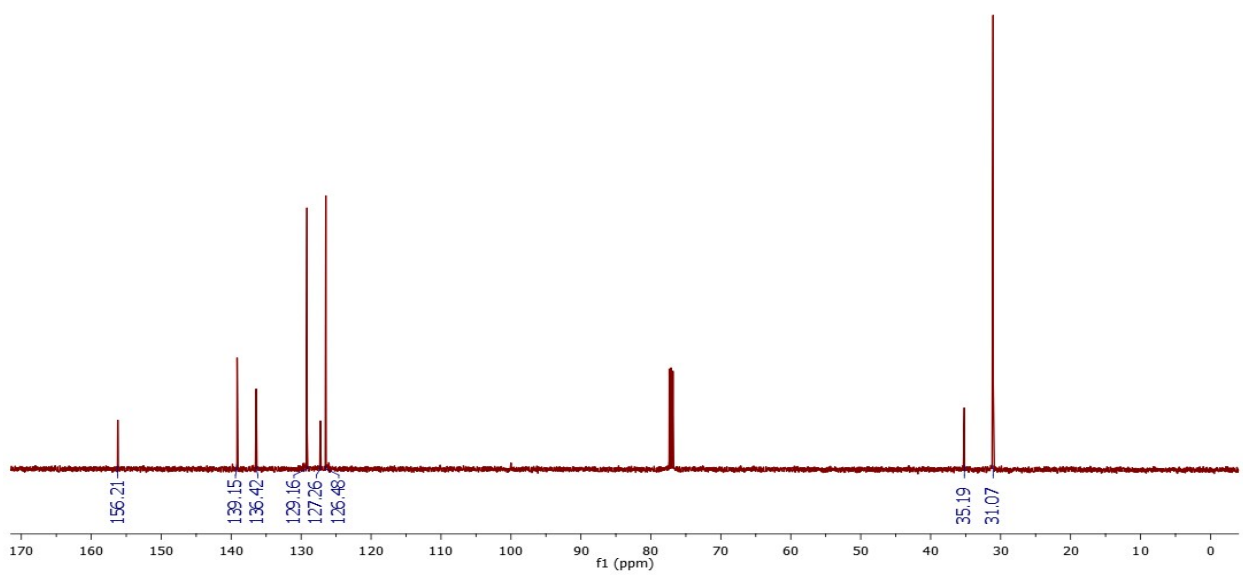

DEPT NMR of (E)-1-(tert-butyl)-4-(2-nitrovinyl)benzene (2f)

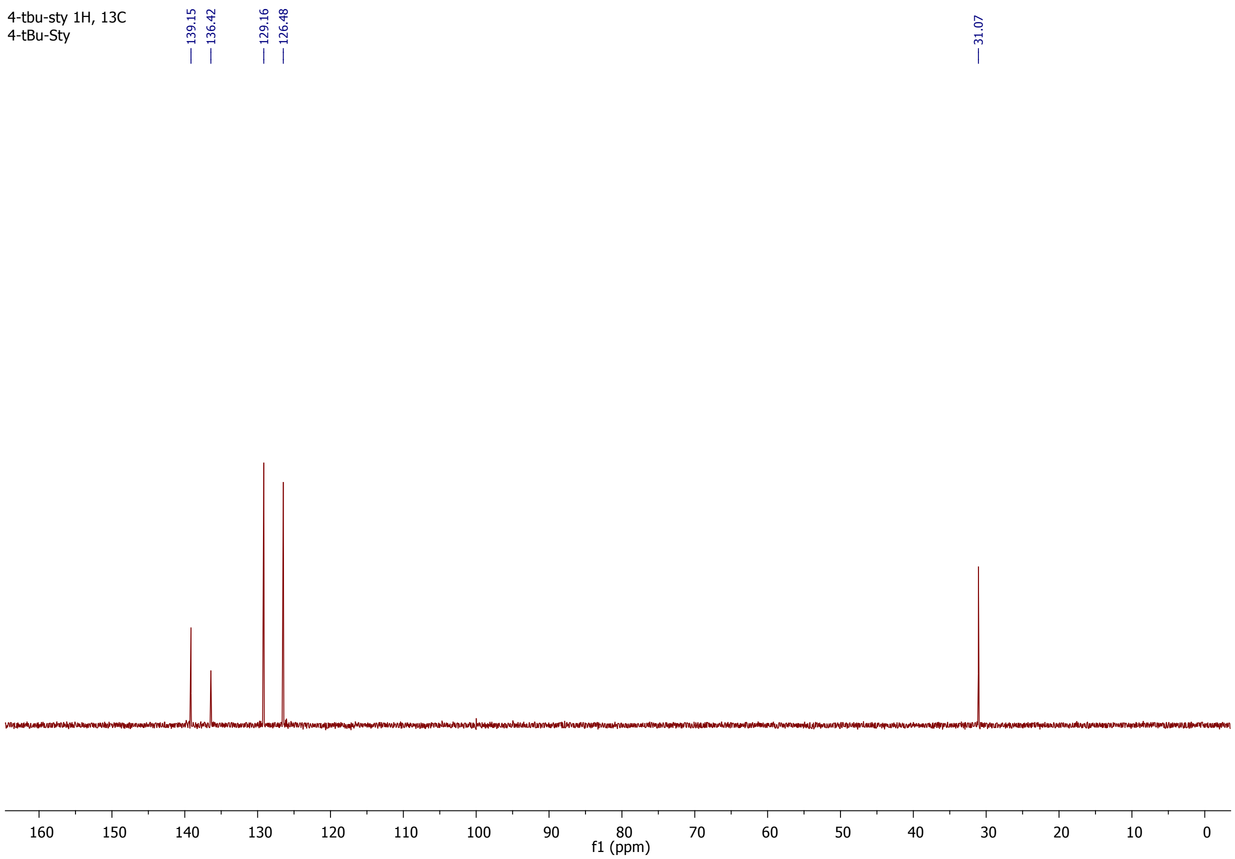

GC-MS of (E)-1-(tert-butyl)-4-(2-nitrovinyl)benzene (2f)

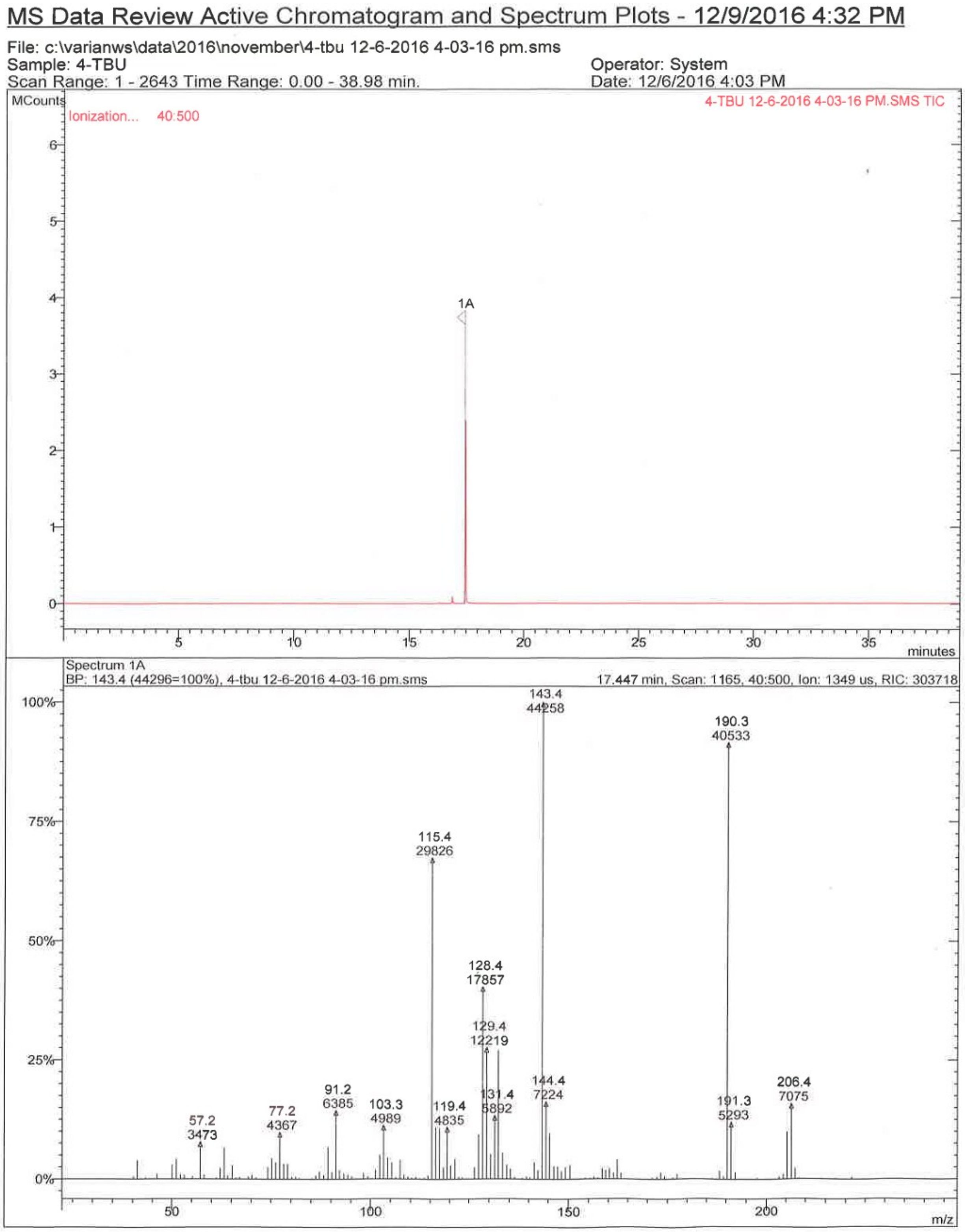

^{1}H NMR of (E)-1,4-dimethyl-2-(2-nitrovinyl)benzene (2g)[5]

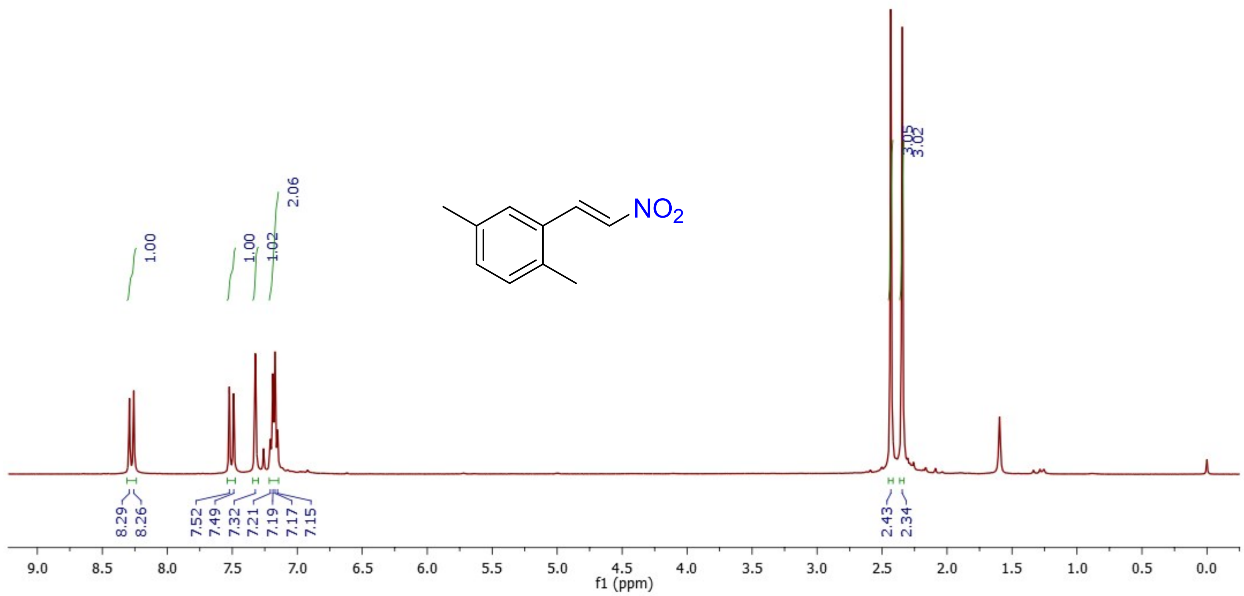

^{13}C NMR of (E)-1,4-dimethyl-2-(2-nitrovinyl)benzene (2g)

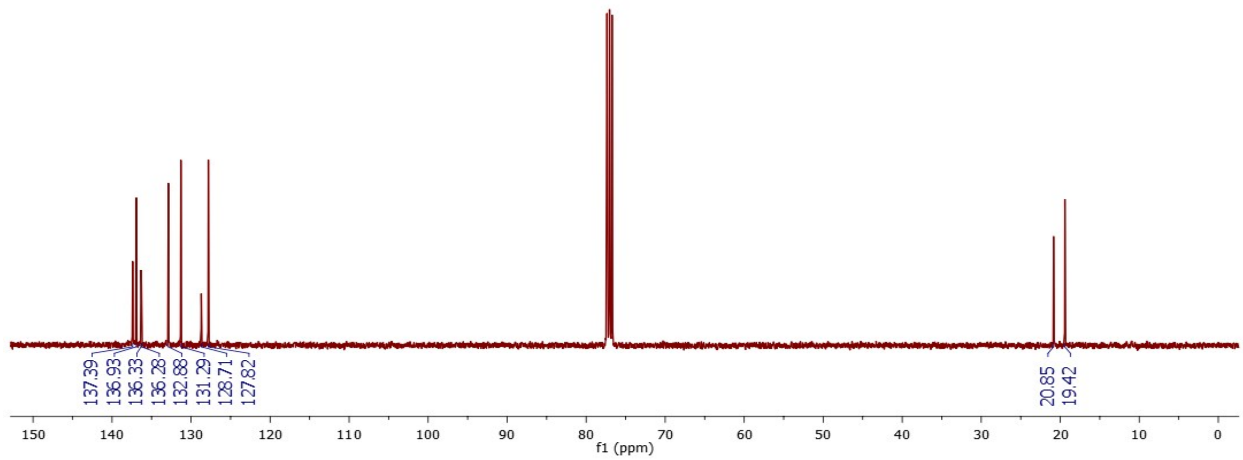

DEPT NMR of (E)-1,4-dimethyl-2-(2-nitrovinyl)benzene (2g)

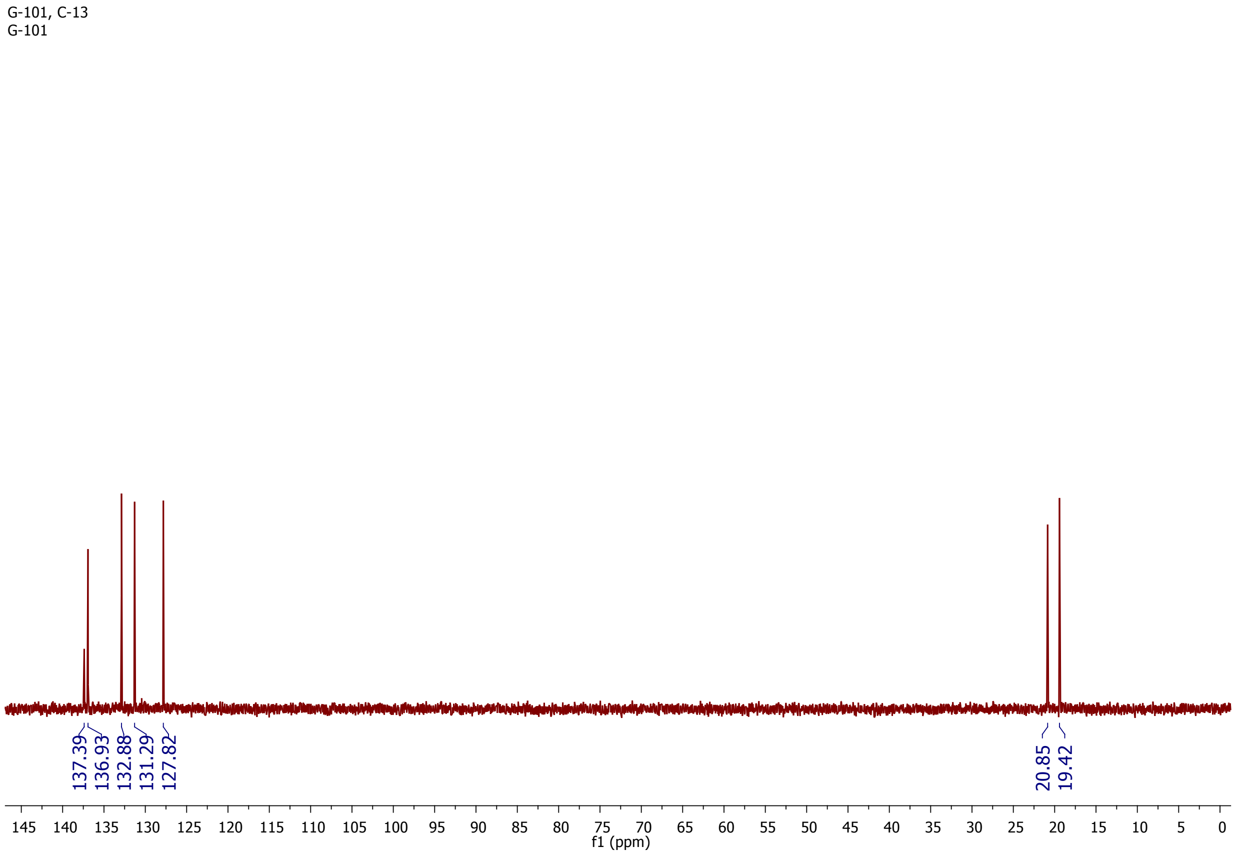

GC-MS of (E)-1,4-dimethyl-2-(2-nitrovinyl)benzene (2g)

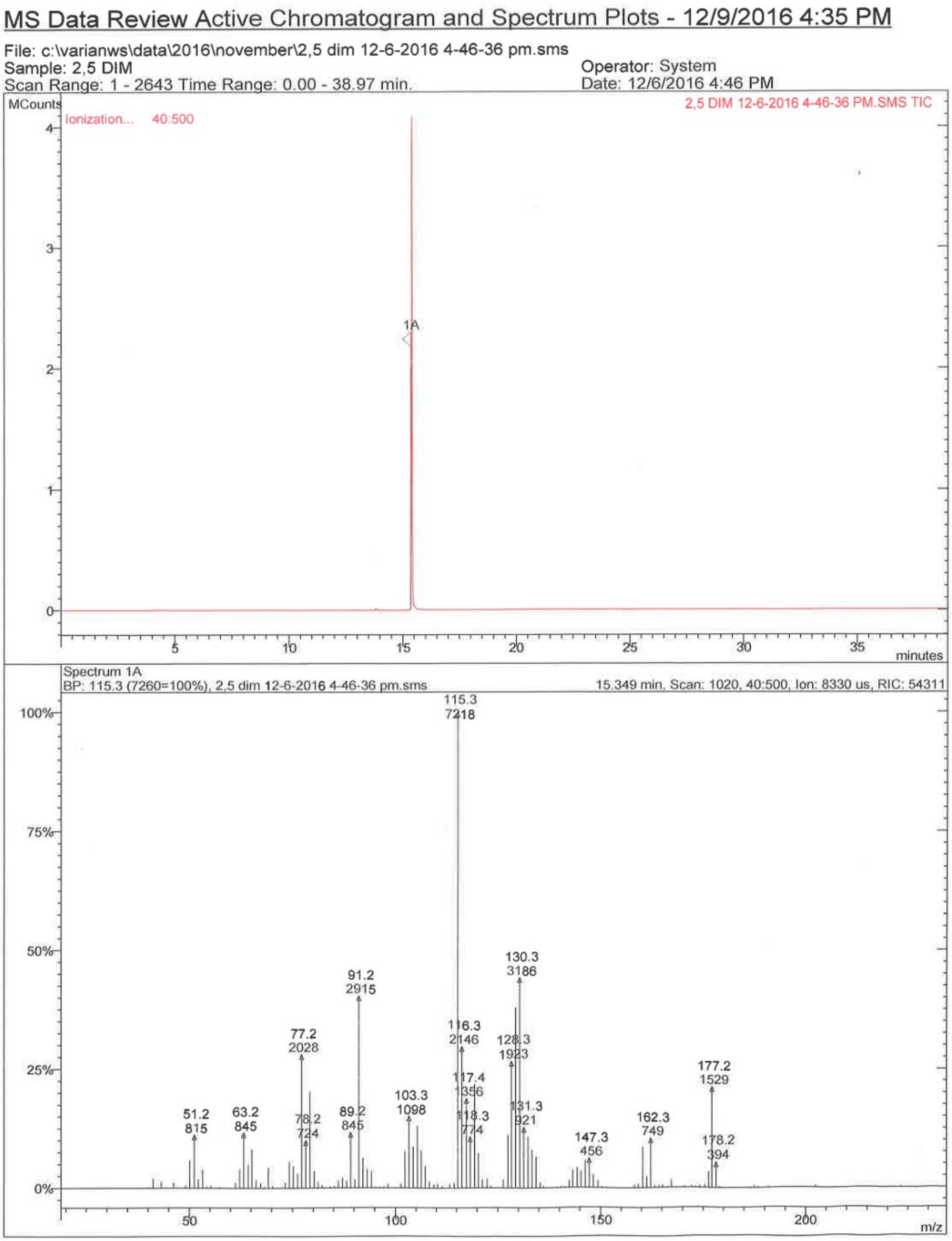

^{1}H NMR spectra of (E)-2,4-dimethyl-1-(2-nitrovinyl)benzene (2h)[6]

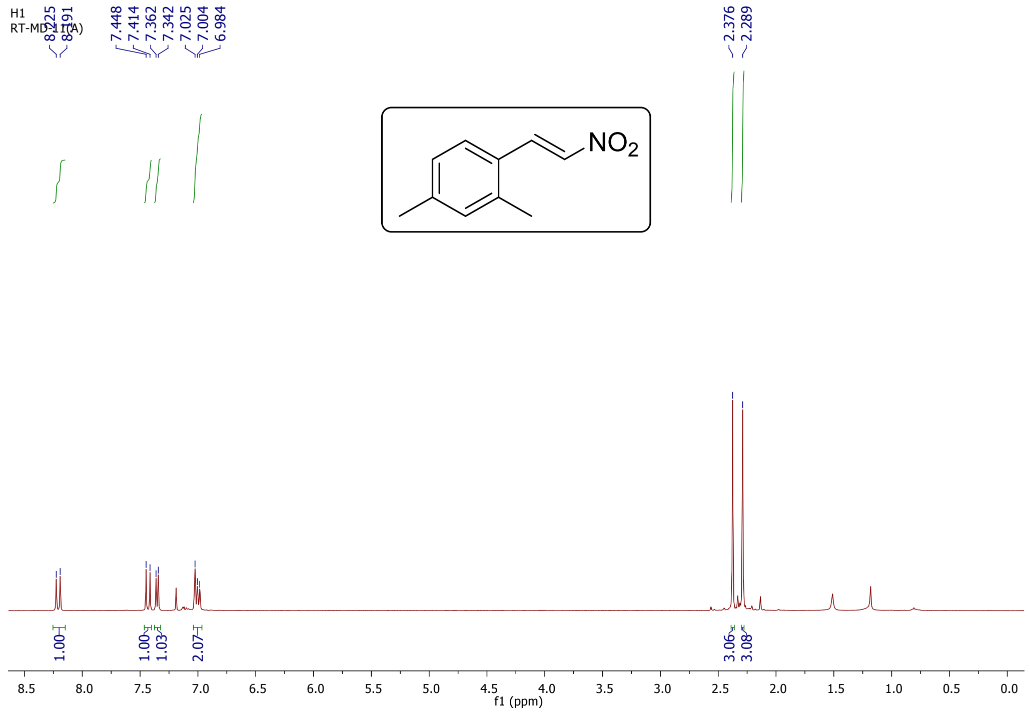

^{13}C NMR Spectra of (E)-2,4-dimethyl-1-(2-nitrovinyl)benzene (2h)

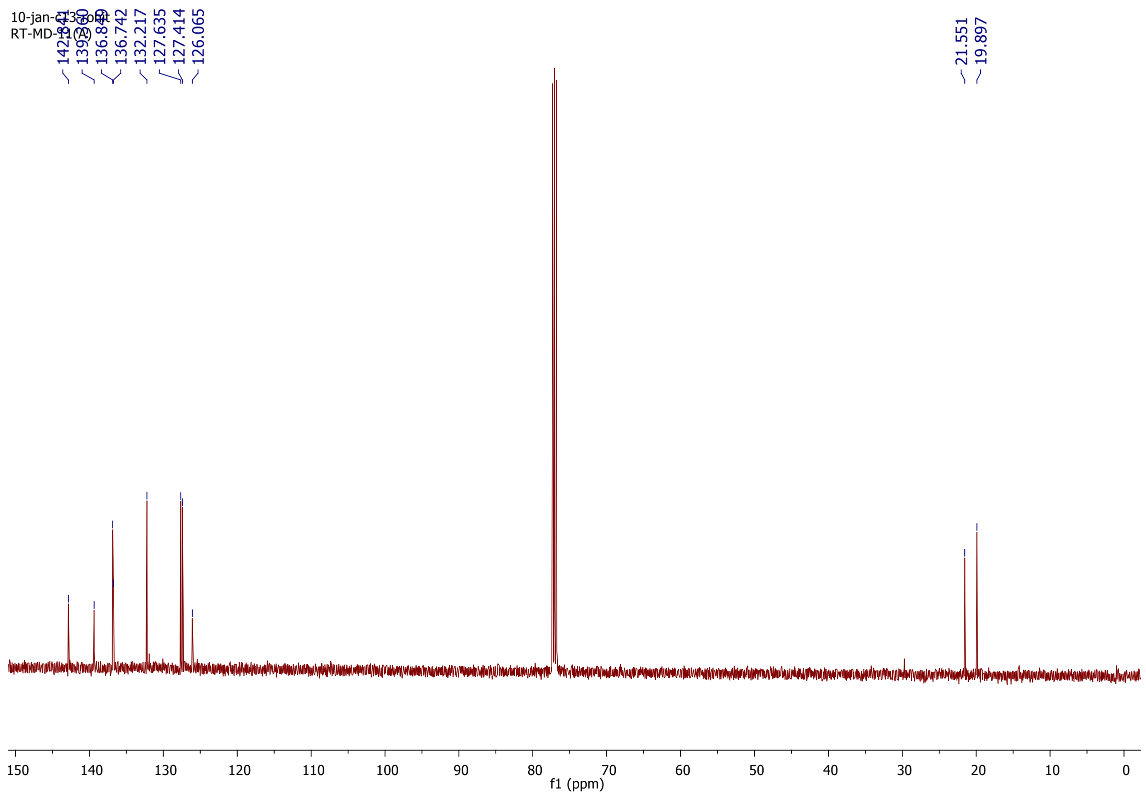

GC-MS of compound(E)-2,4-dimethyl-1-(2-nitrovinyl)benzene(2h)

**Data Filename**	04-06-18AMIT08.D	**Sample Name**	MD-11 A
**Sample Type**	Sample	**Position**	8
**Instrument Name**	GCMS	**User Name**	manager
**Acq Method**	AMIT.M	**Acquired Time**	6/5/2018 7:45:39 PM
**IRM Calibration Status**	Not Applicable	**DA Method**	Default.m
**Comment**			

**Expected Barcode**		**Sample Amount**	
**Vial**	8	**SeqPath**	C:\msdchem\1\sequence\june 4.s
**TuneName**	12 atune.u	**TunePath**	C:\msdchem\1\5975\
**TuneDateStamp**	43251.42431	**OperatorName**	manager
**RunCompletedFlag**	TRUE		

User Chromatograms

Fragmentor Voltage 0 **Collision Energy** 0 **Ionization Mode** Unspecified

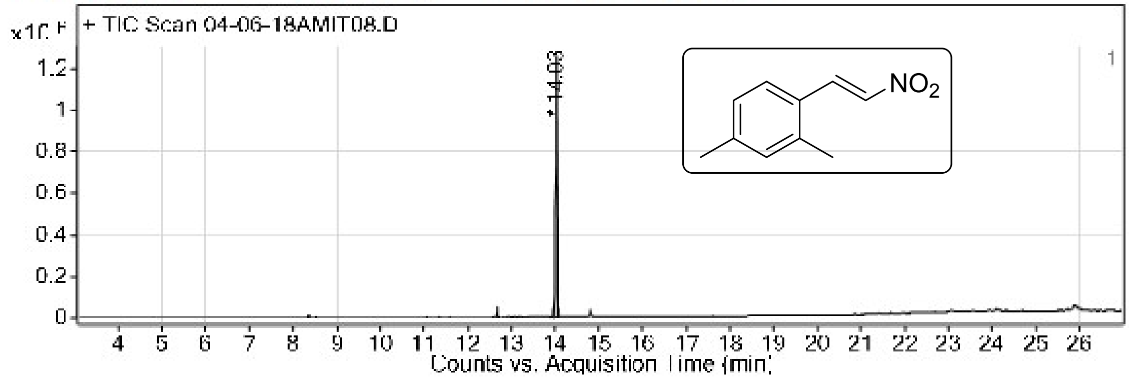

User Spectra

**Spectrum Source**	**Fragmentor Voltage**	**Collision Energy**	**Ionization Mode**
Peak (1) in "+ TIC Scan - 04-06-18AMIT08.D"	0	0	Unspecified

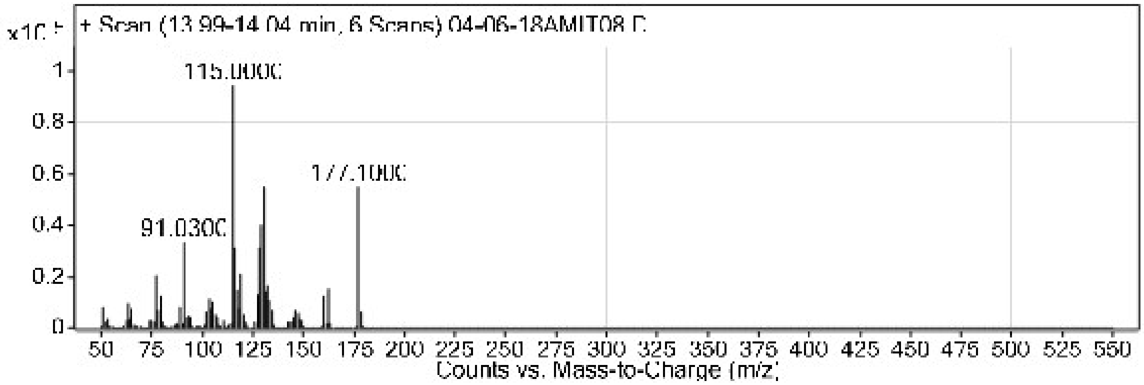

^{1}H NMR of (E)-2,4-dimethoxy-1-(2-nitrovinyl)benzene (2i)[7]

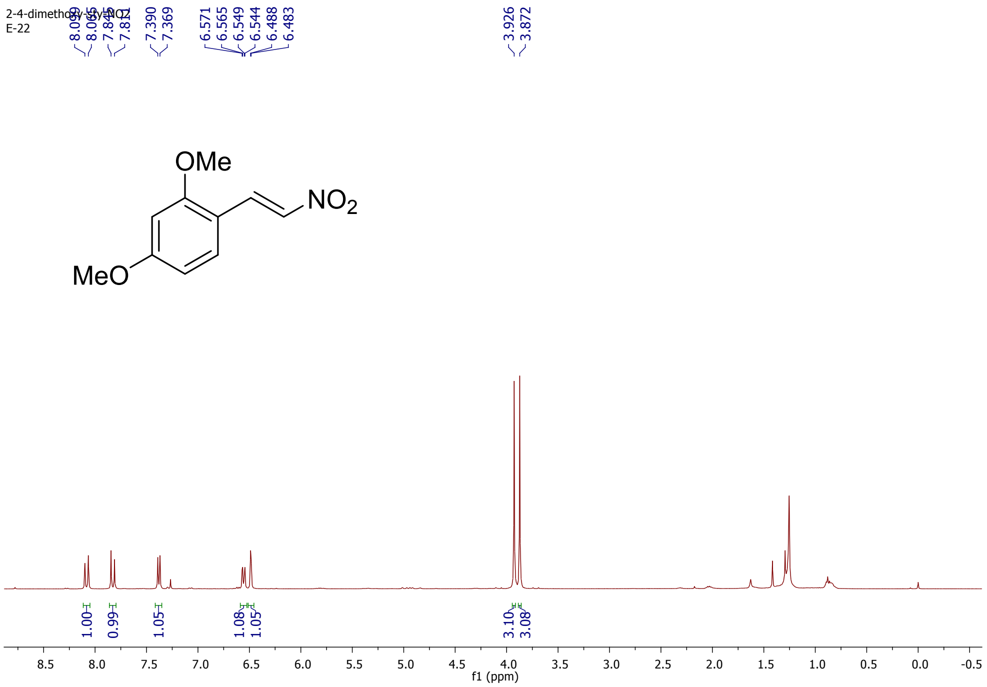

^{13}C NMR (E)-2,4-dimethoxy-1-(2-nitrovinyl)benzene (2i)

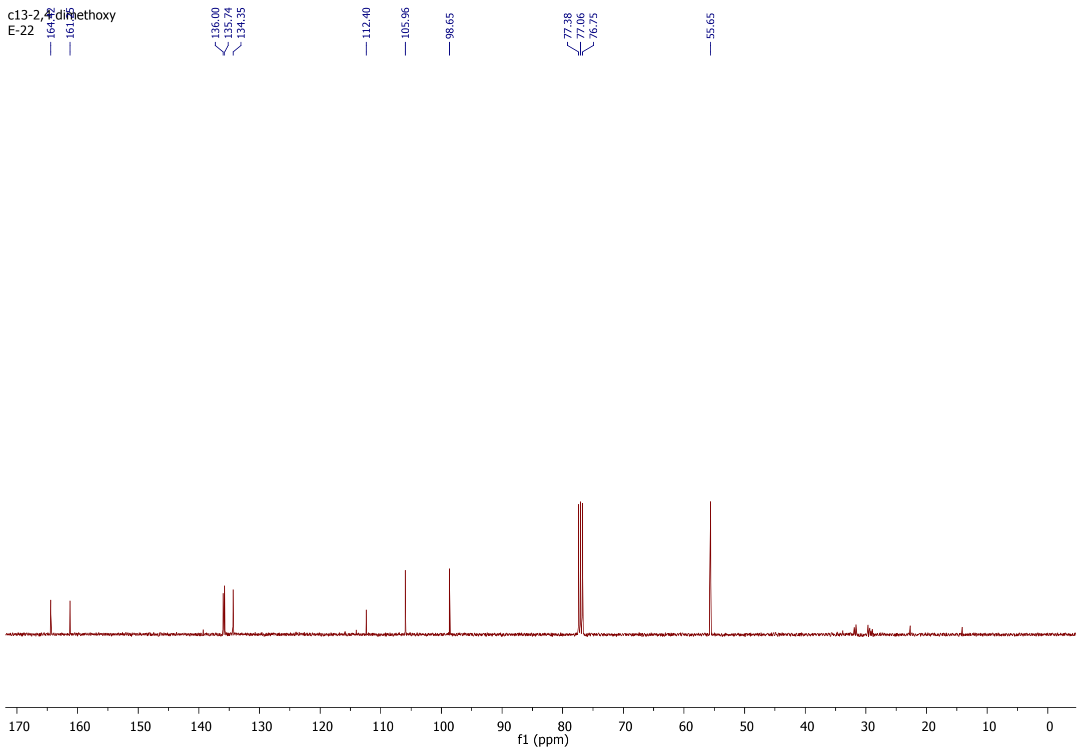

DEPT of (E)-2,4-dimethoxy-1-(2-nitrovinyl)benzene (2i)

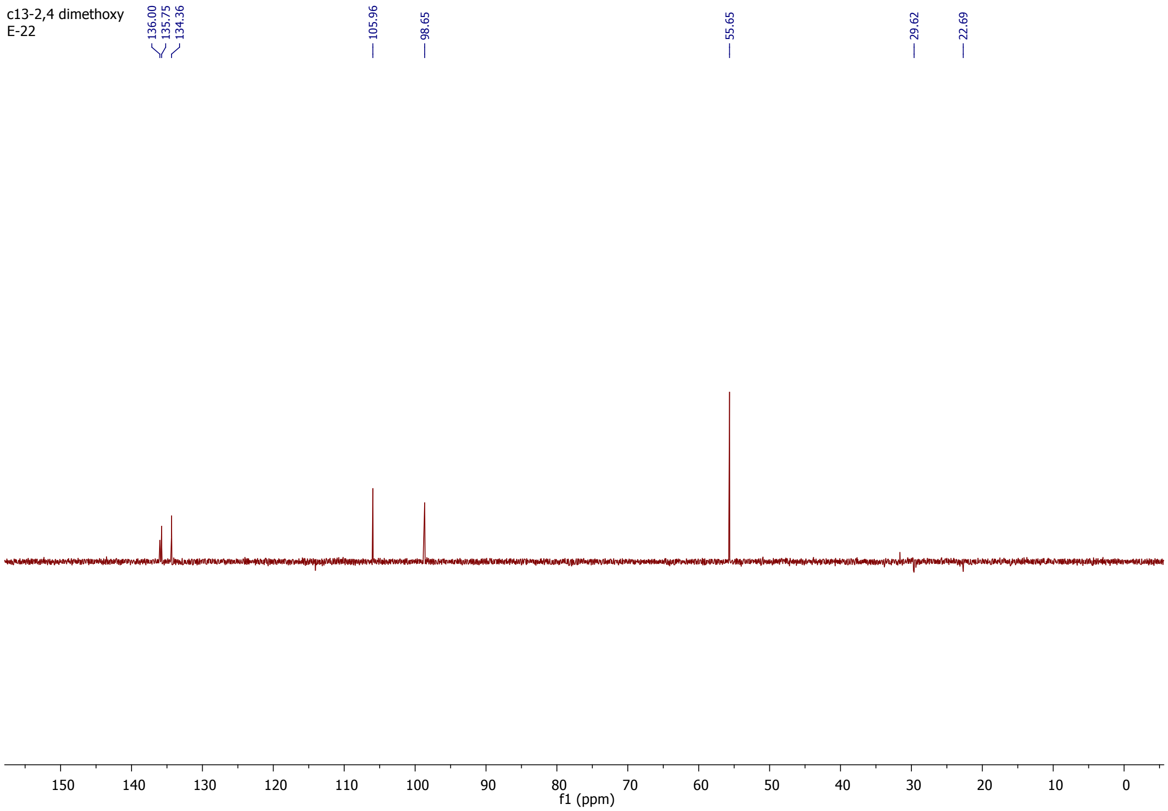

GC-MS of (E)-2,4-dimethoxy-1-(2-nitrovinyl)benzene (2i)

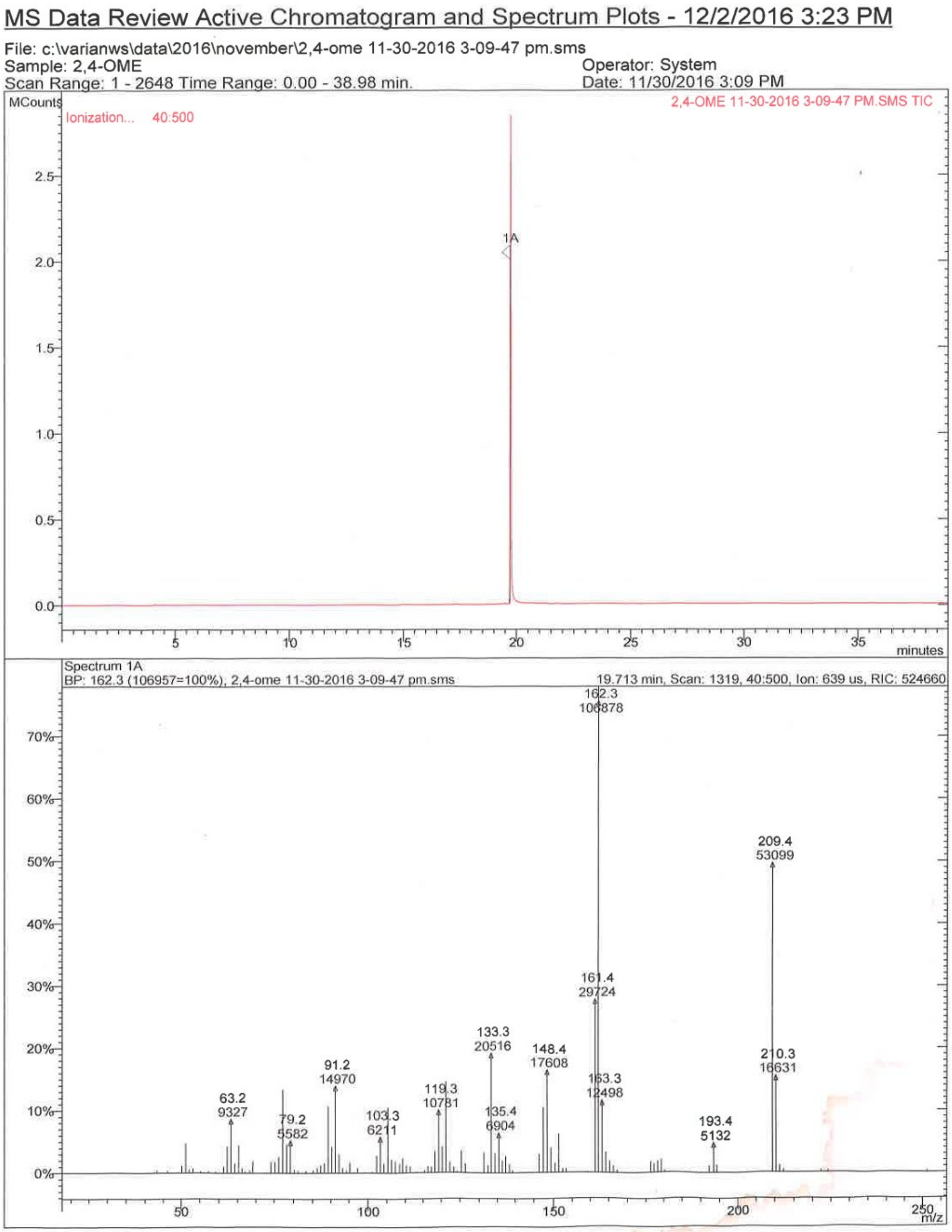

^{1}H NMR of (E)-4-methoxy-2-methyl-1-(2-nitrovinyl)benzene (2j)[8]

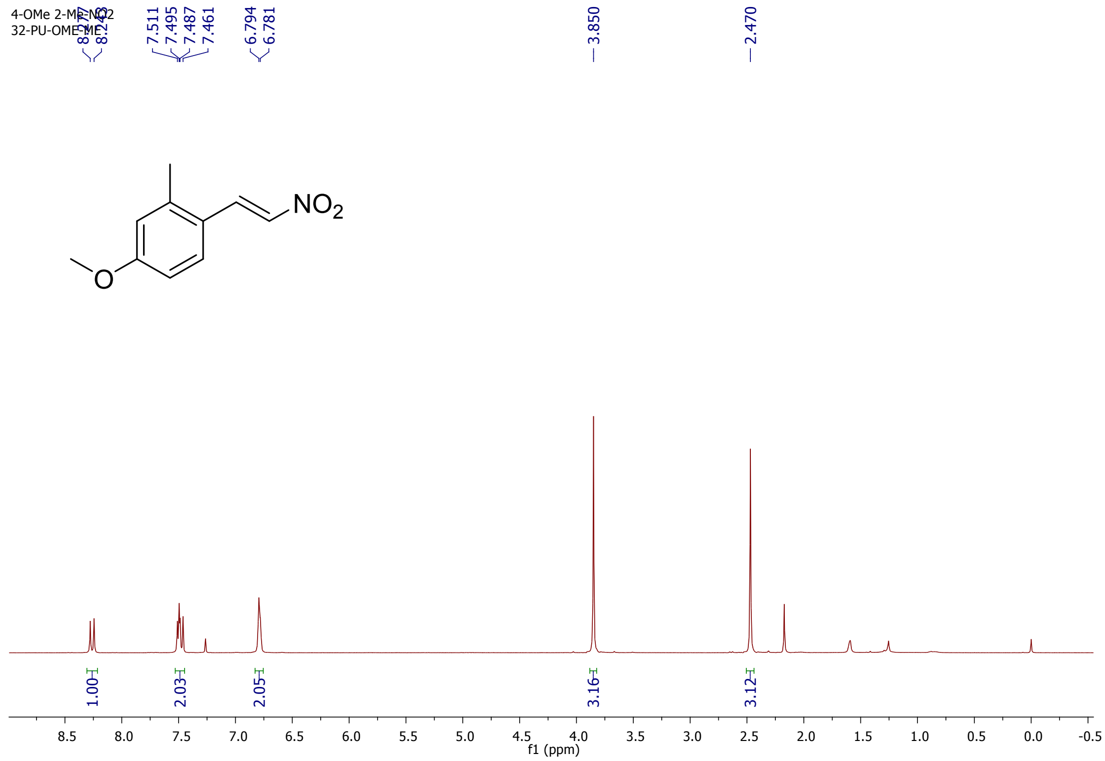

^{13}C NMR of (E)-4-methoxy-2-methyl-1-(2-nitrovinyl)benzene (2j)

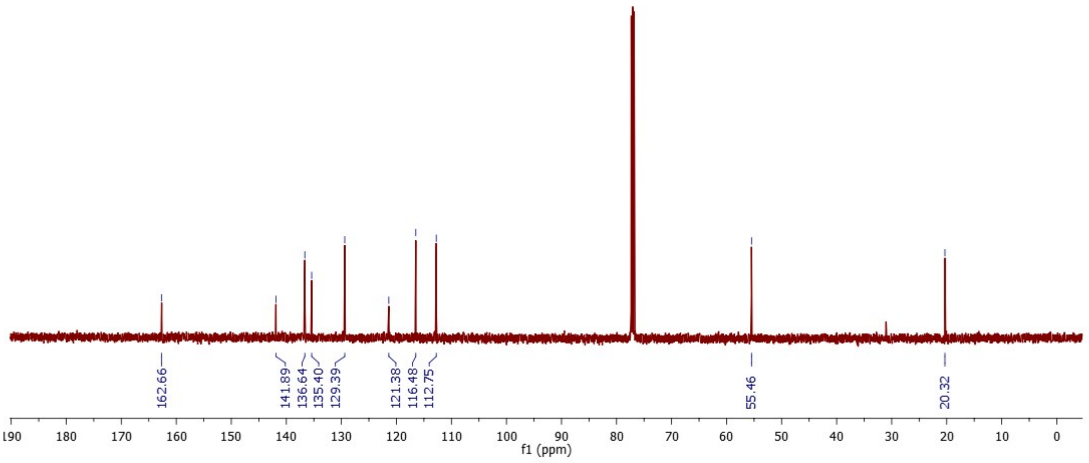

DEPT NMR of (E)-4-methoxy-2-methyl-1-(2-nitrovinyl)benzene (2j)

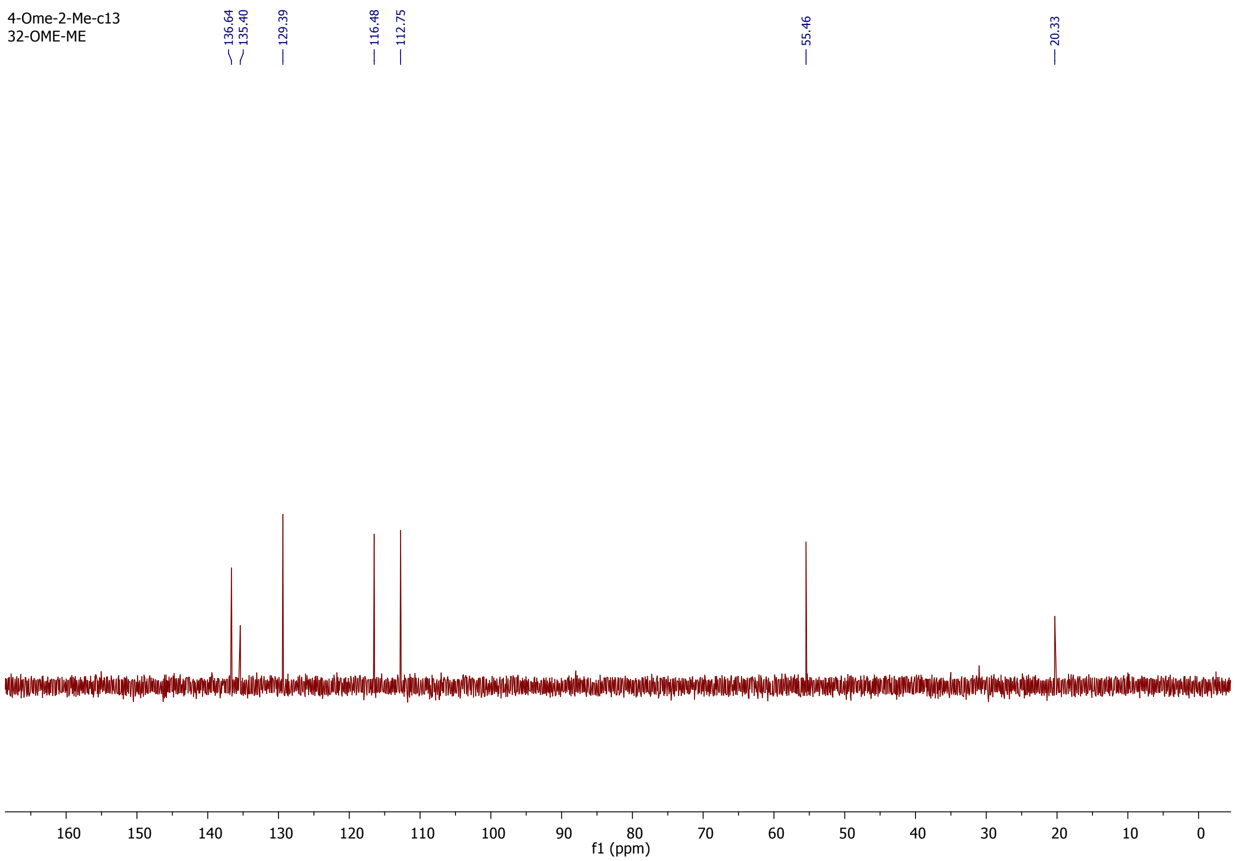

GC-MS of (E)-4-methoxy-2-methyl-1-(2-nitrovinyl)benzene (2j)

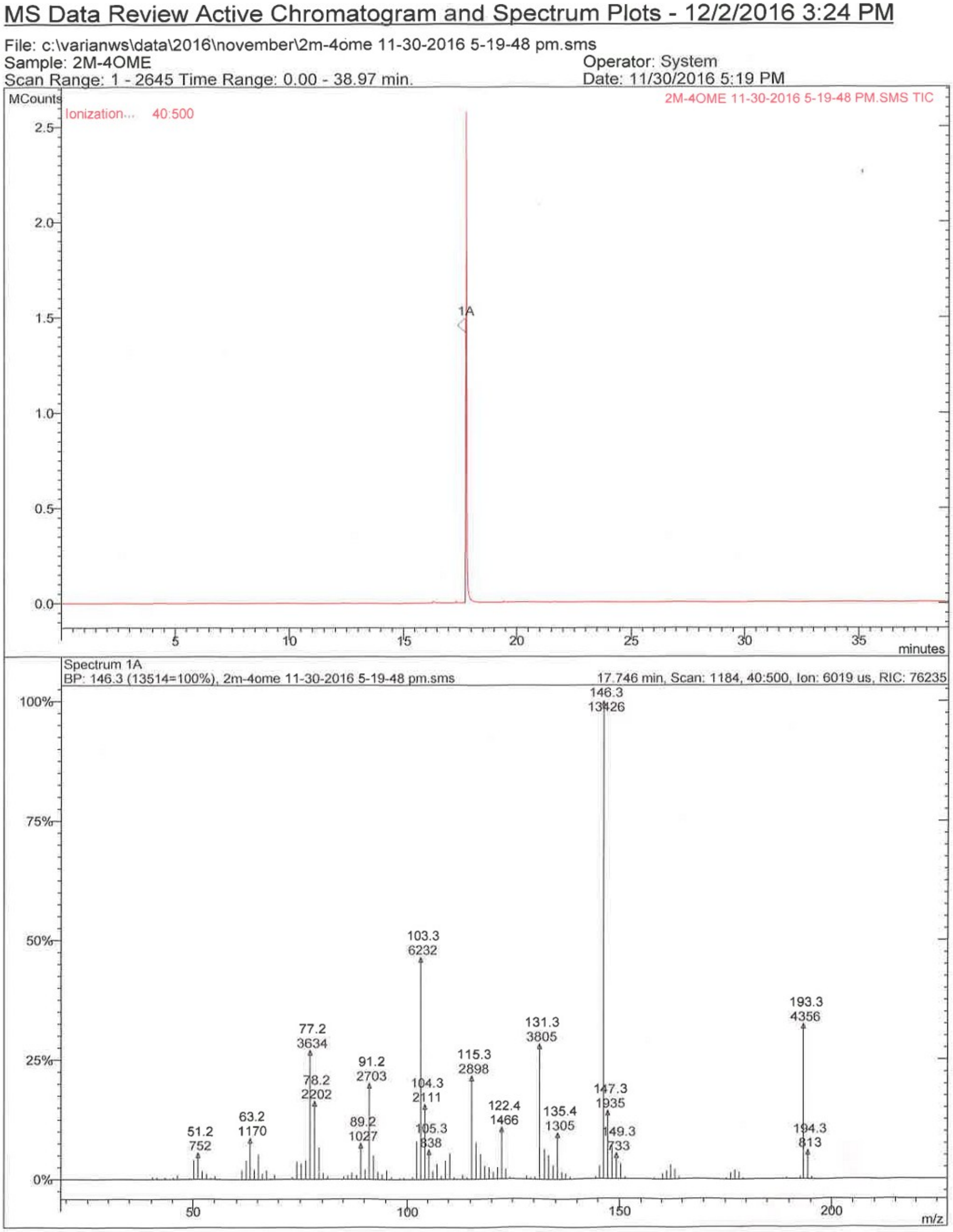

^{1}H NMR of (E)-2-fluoro-4-methoxy-1-(2-nitrovinyl)benzene (2k)[9]

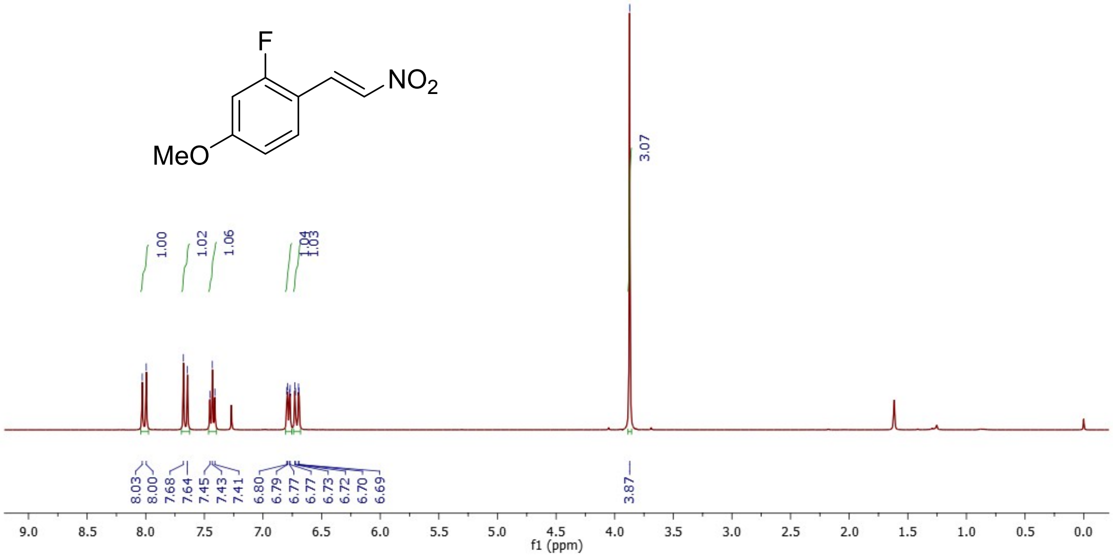

^{13}C NMR of (E)-2-fluoro-4-methoxy-1-(2-nitrovinyl)benzene (2k)

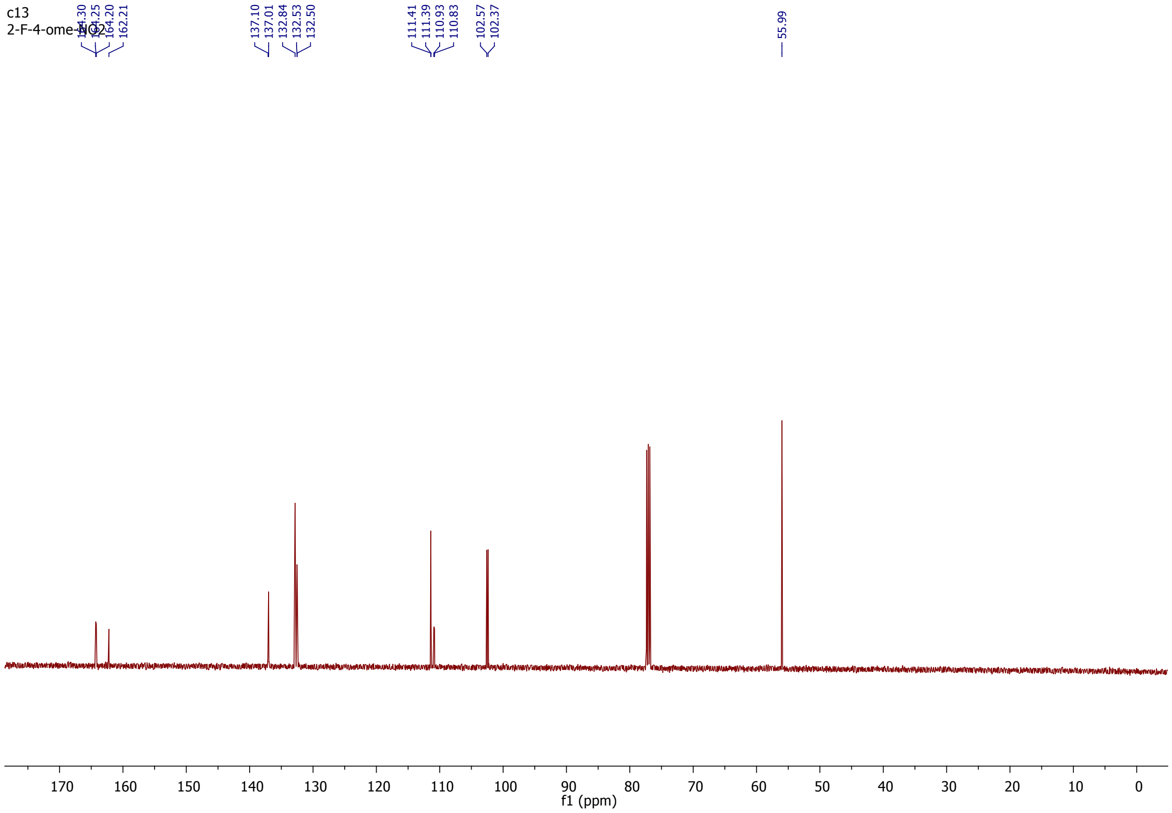

DEPT NMR of (E)-2-fluoro-4-methoxy-1-(2-nitrovinyl)benzene (2k)

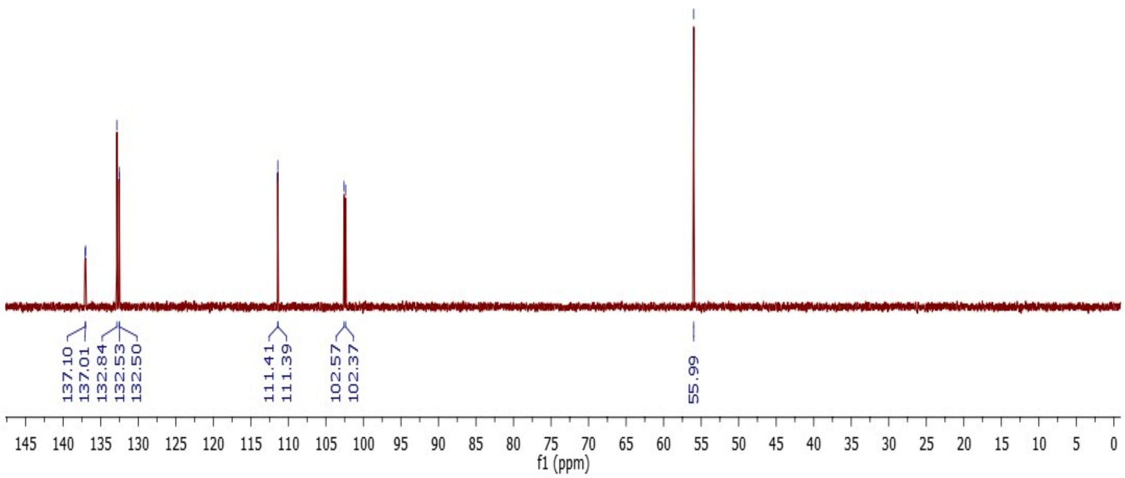

GC-MS of (E)-2-fluoro-4-methoxy-1-(2-nitrovinyl)benzene (2k)

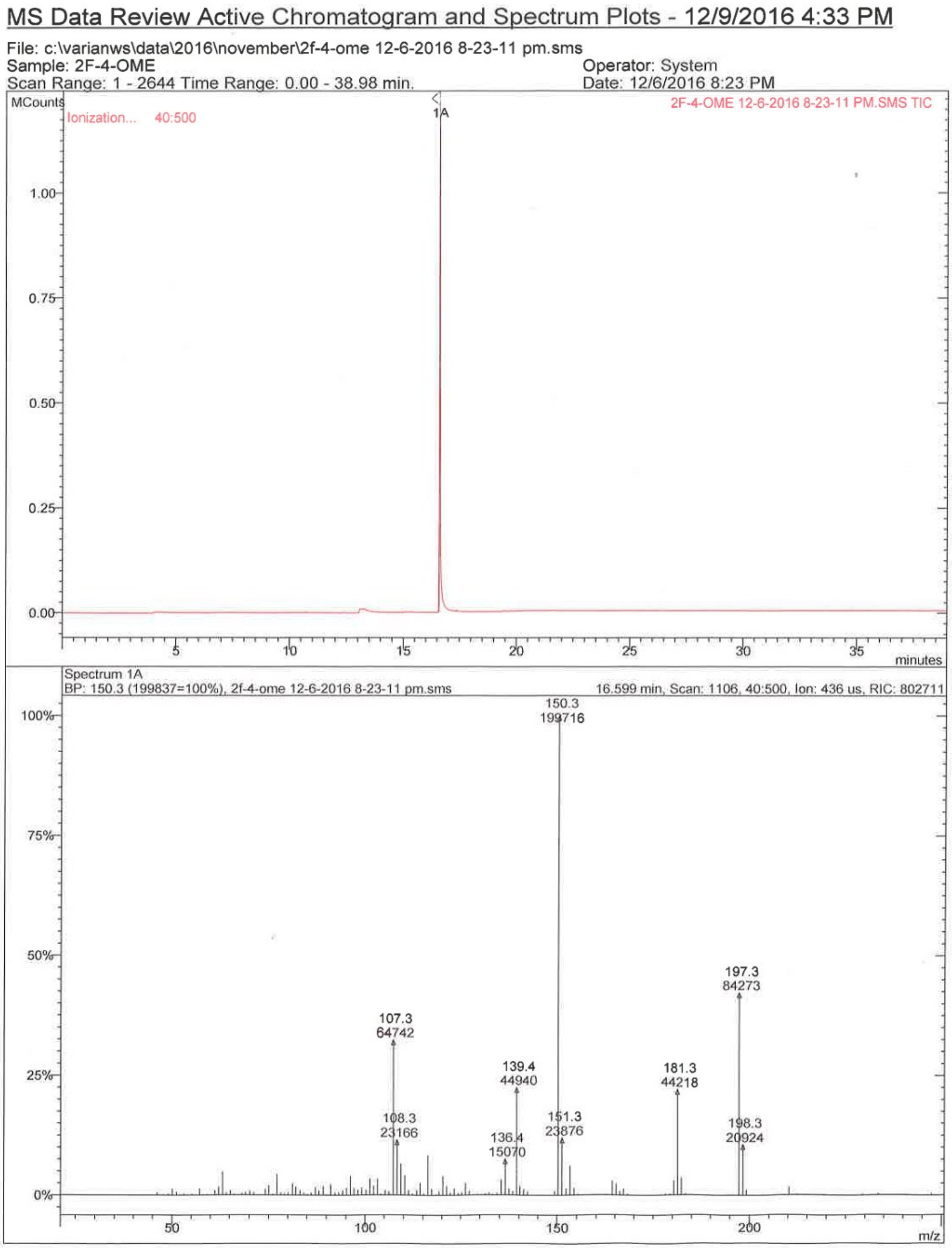

^{1}H NMR of (E)-1,2,3-trimethoxy-5-(2-nitrovinyl)benzene (2l)[10]

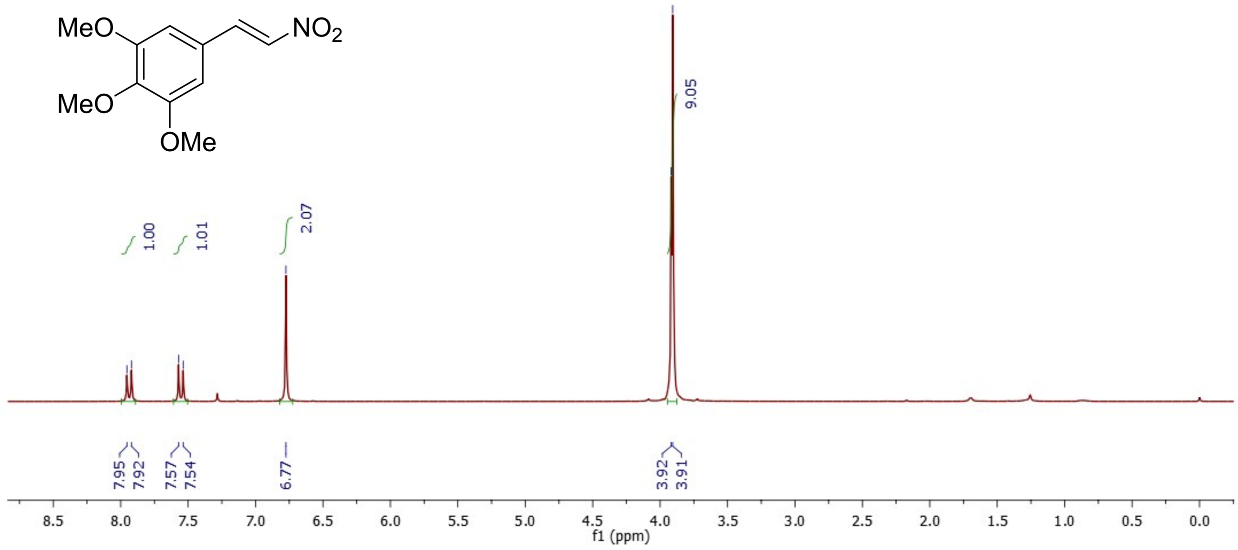

^{13}C NMR of (E)-1,2,3-trimethoxy-5-(2-nitrovinyl)benzene (2l)

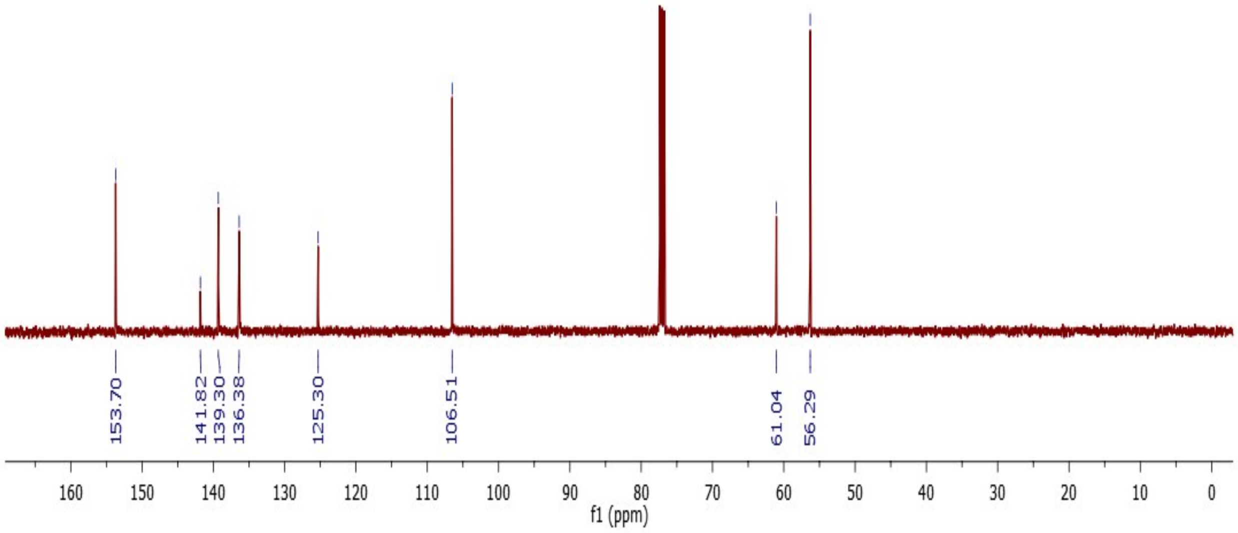

DEPT NMR of (E)-1,2,3-trimethoxy-5-(2-nitrovinyl)benzene (2l)

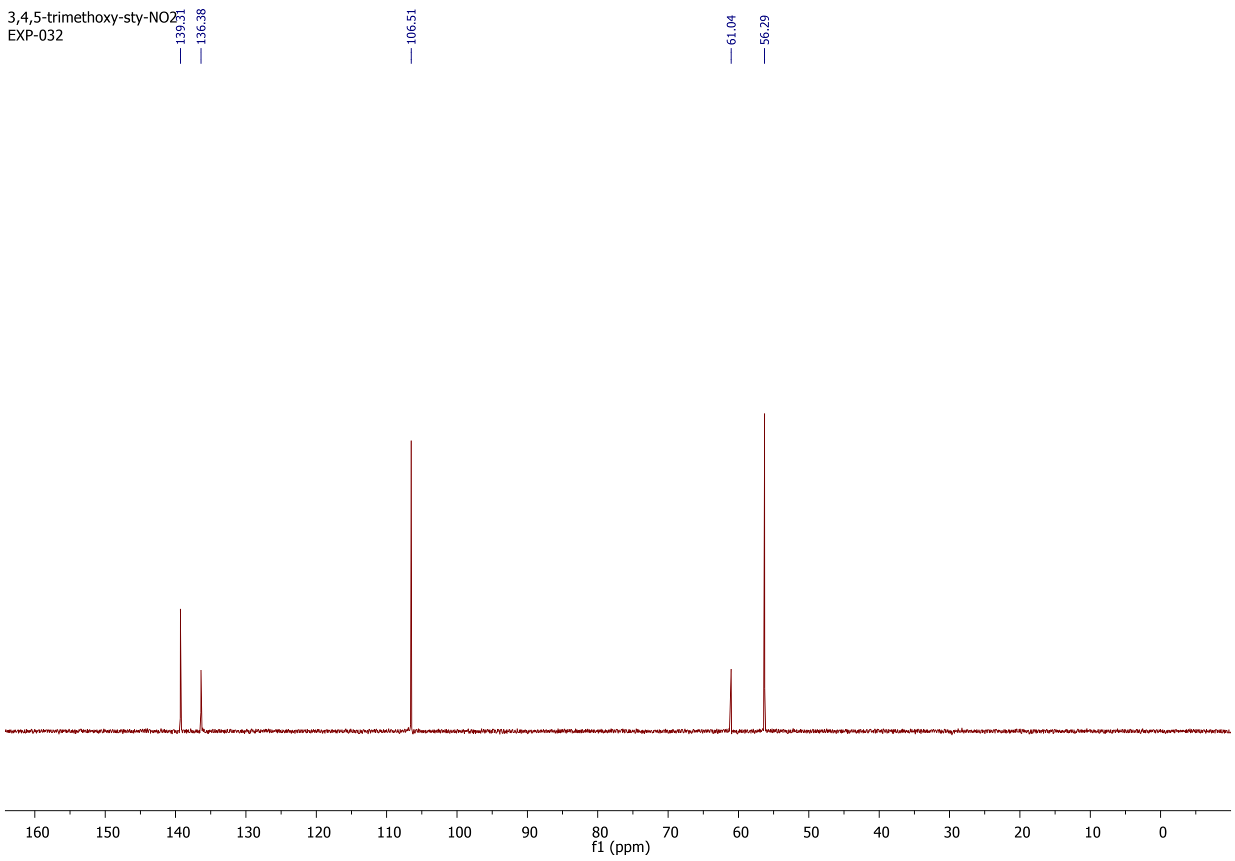

GC-MS of (E)-1,2,3-trimethoxy-5-(2-nitrovinyl)benzene (2l)

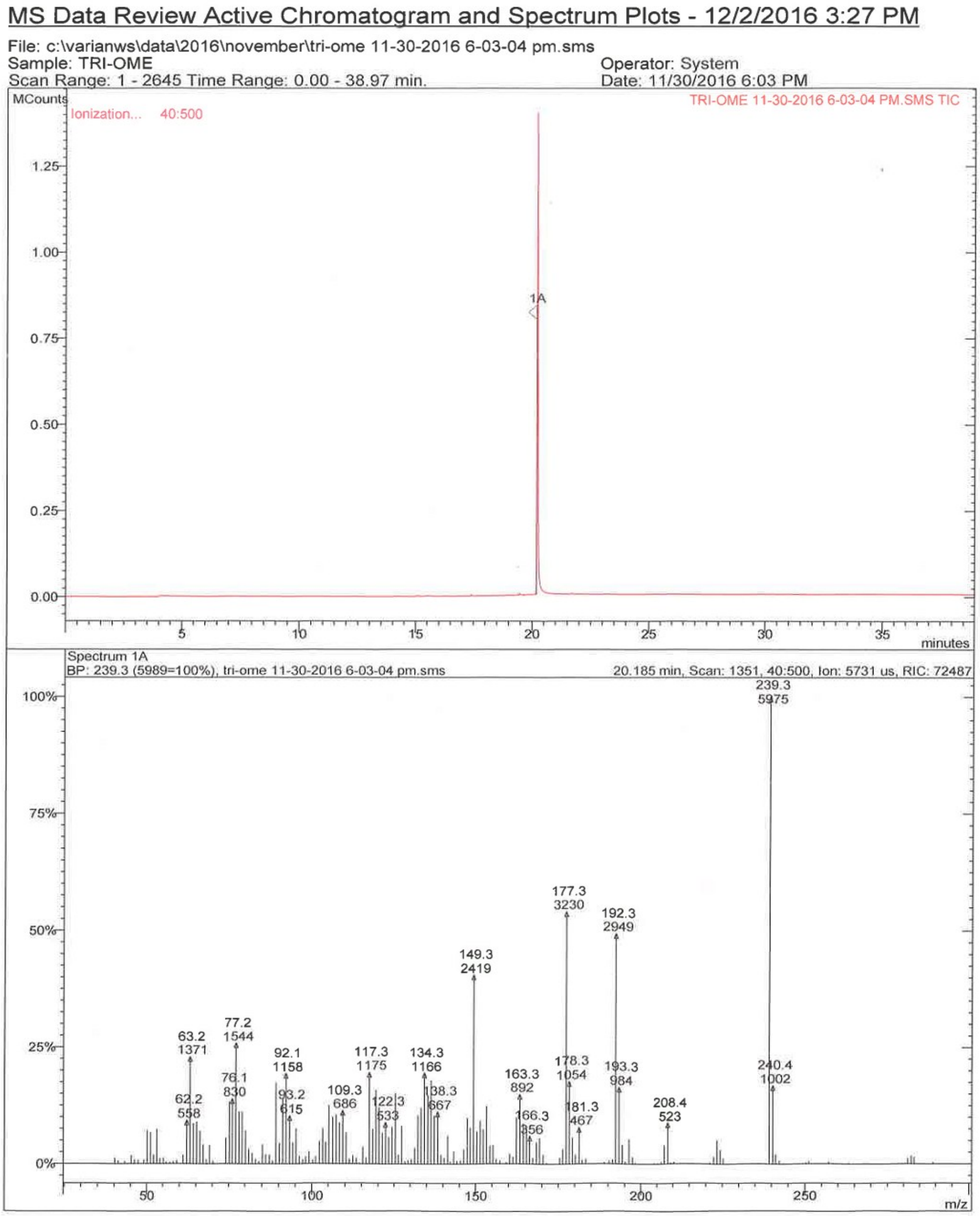

^{1}H NMR of (E)-1-fluoro-4-(2-nitrovinyl)benzene (2m)[11]

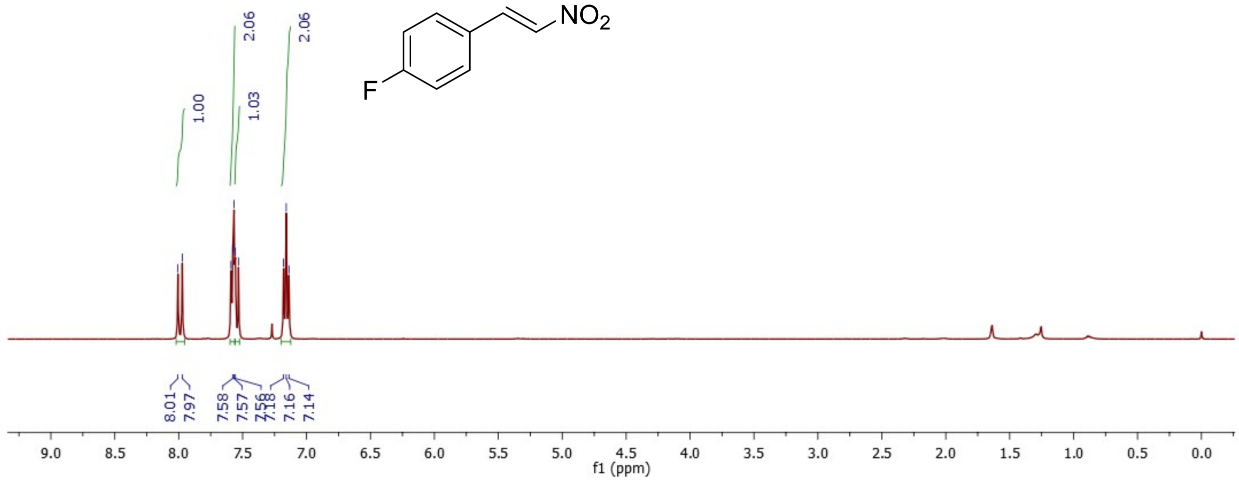

^{13}C NMR of (E)-1-fluoro-4-(2-nitrovinyl)benzene (2m)

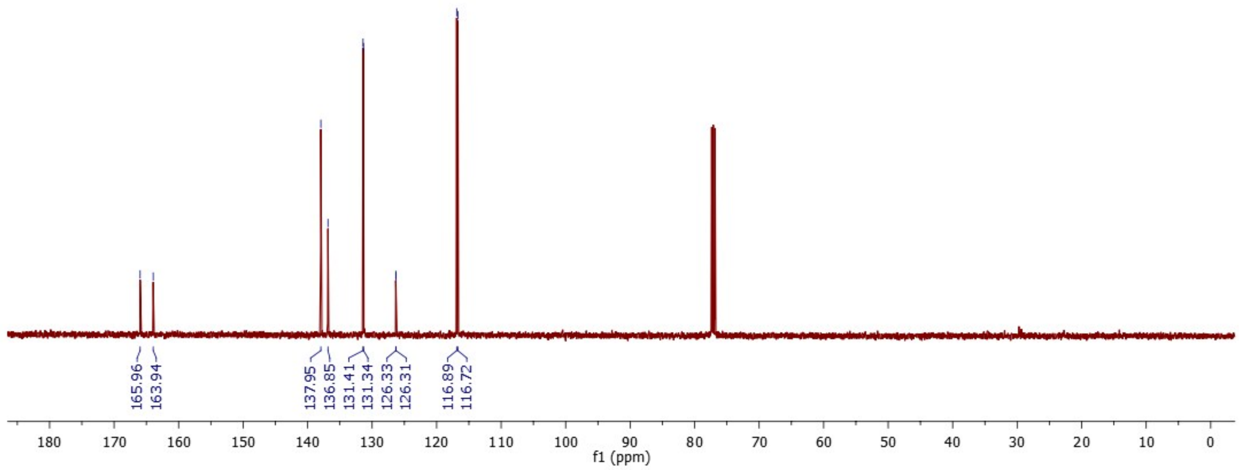

DEPT NMR of (E)-1-fluoro-4-(2-nitrovinyl)benzene (2m)

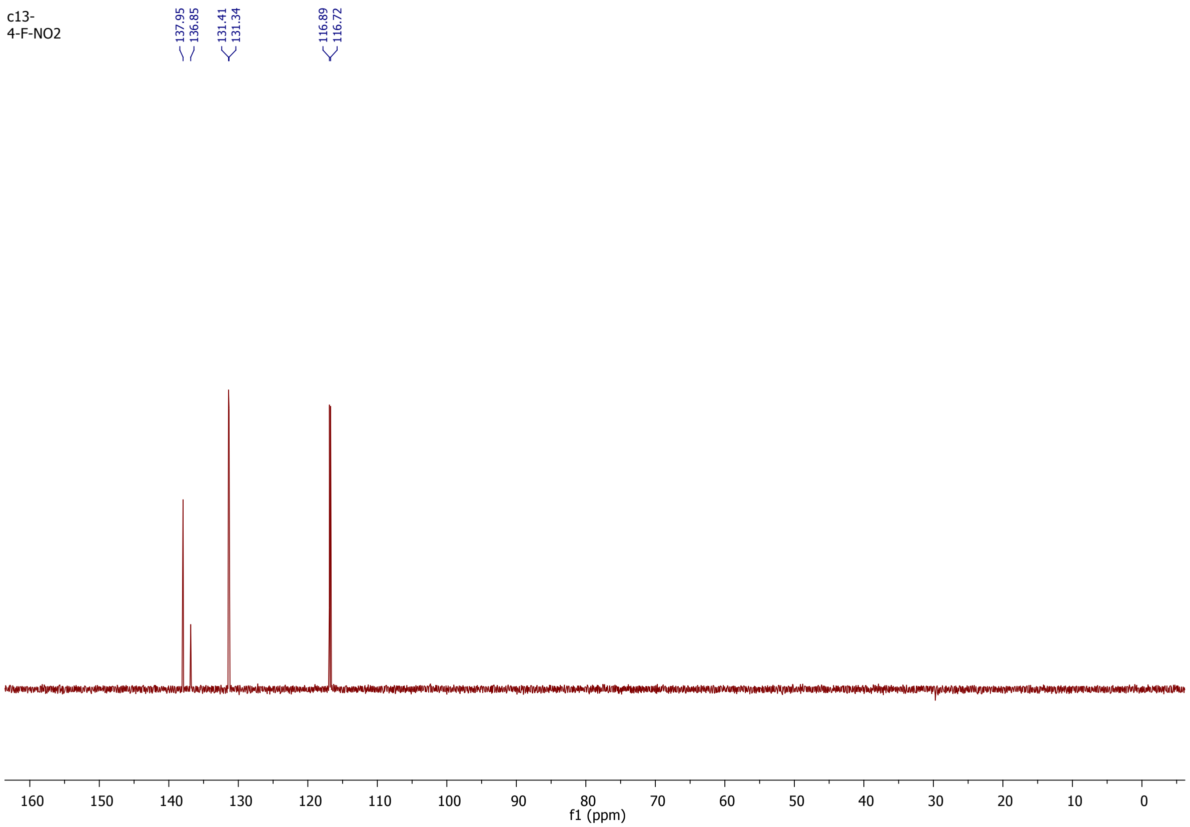

GC-MS of (E)-1-fluoro-4-(2-nitrovinyl)benzene (2m)

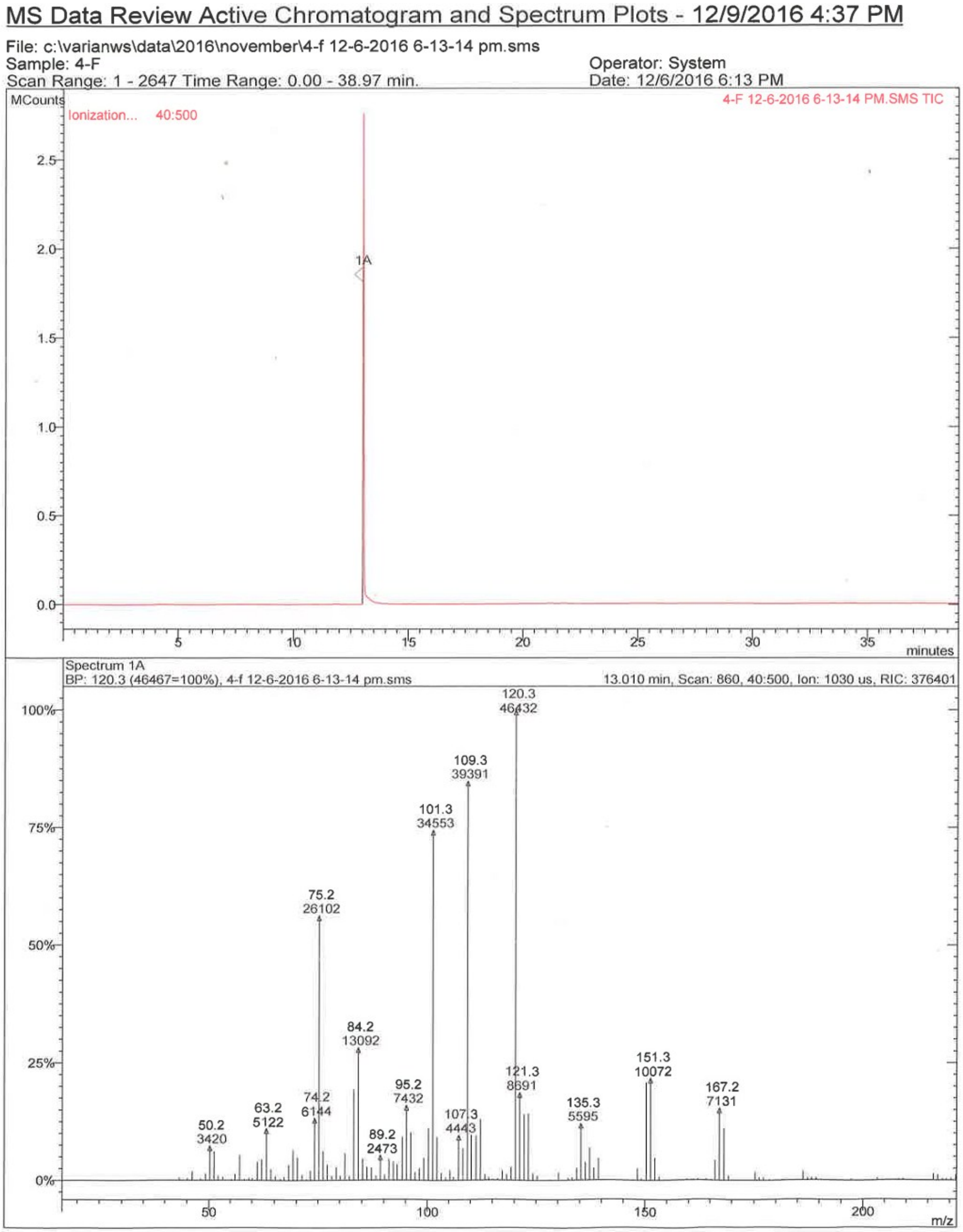

^{1}H NMR of (E)-1-chloro-4-(2-nitrovinyl)benzene (2n)[11]

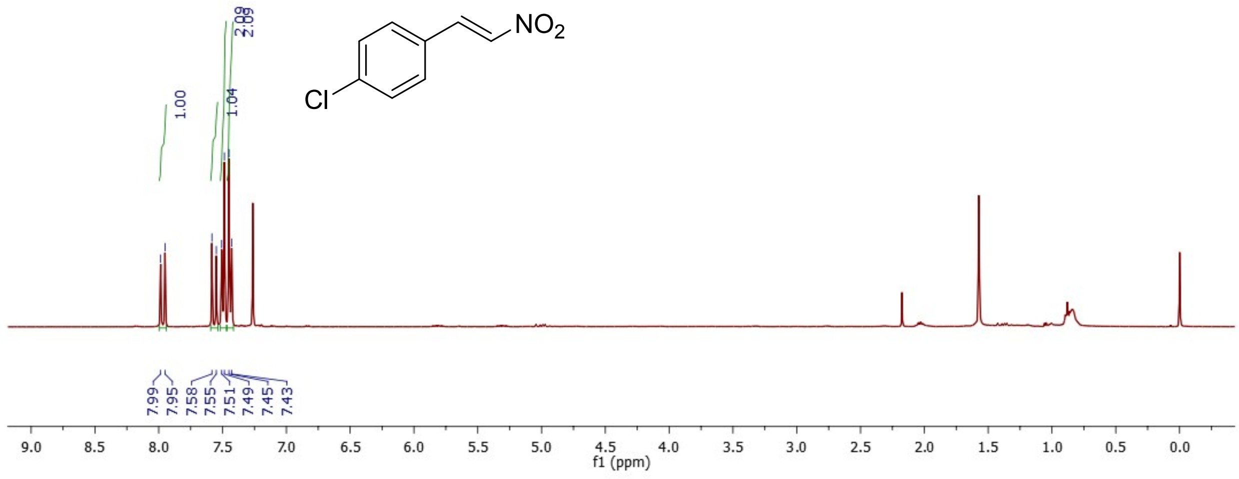

GC-MS of (E)-1-chloro-4-(2-nitrovinyl)benzene (2n)

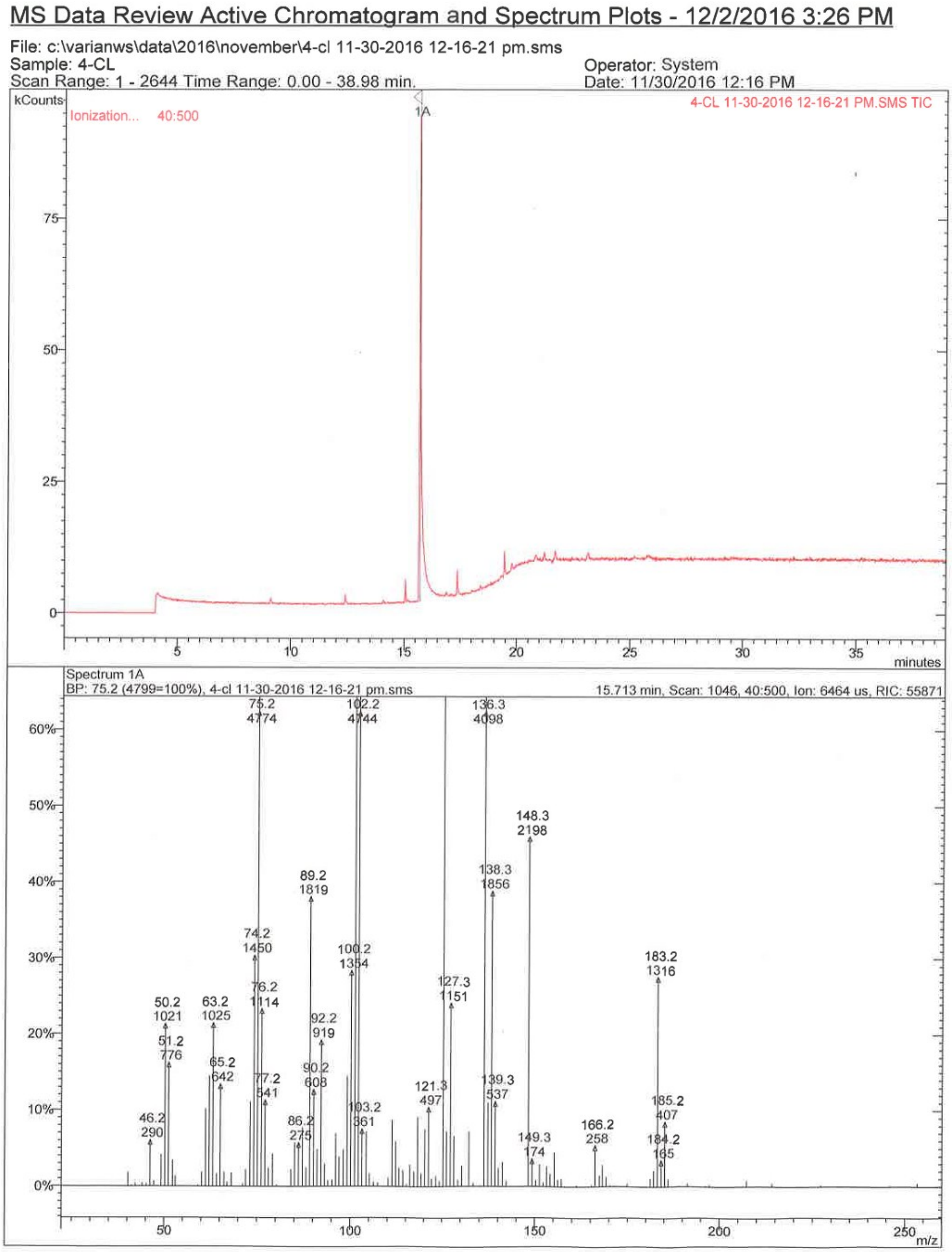

^1H NMR of (E)-1-bromo-4-(2-nitrovinyl)benzene (2o)[11]

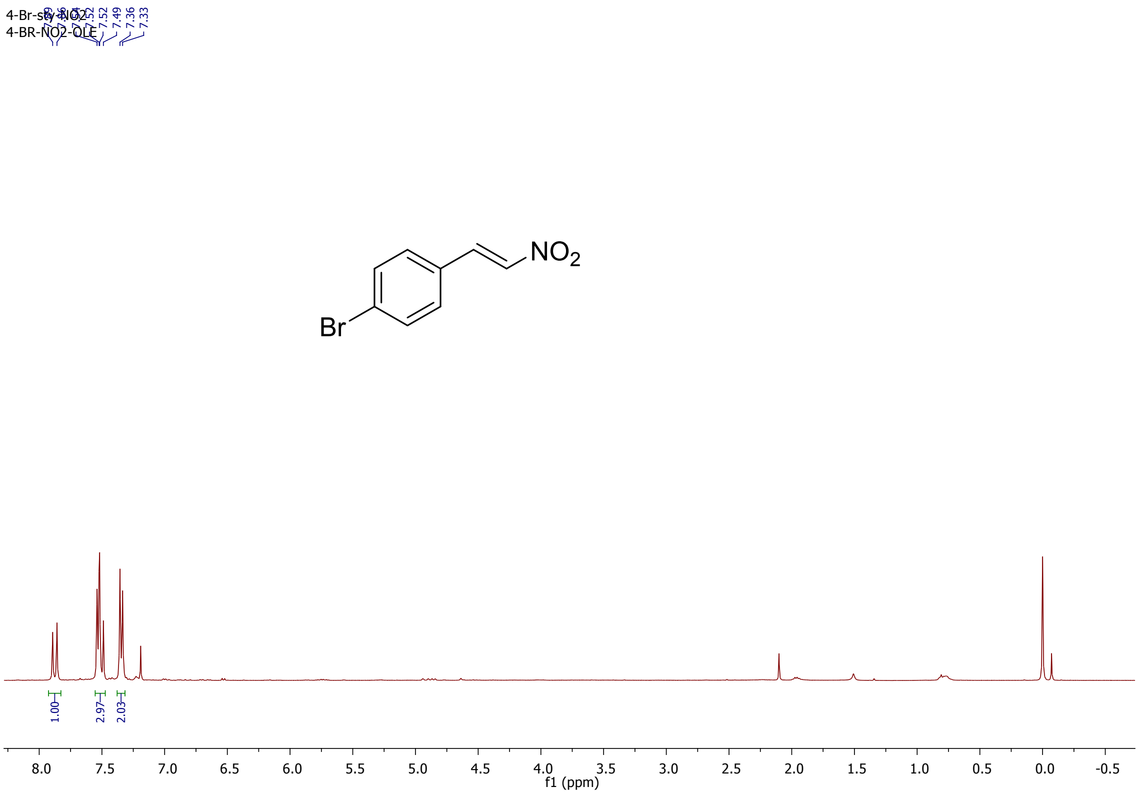

^{13}C NMR of (E)-1-bromo-4-(2-nitrovinyl)benzene (2o)

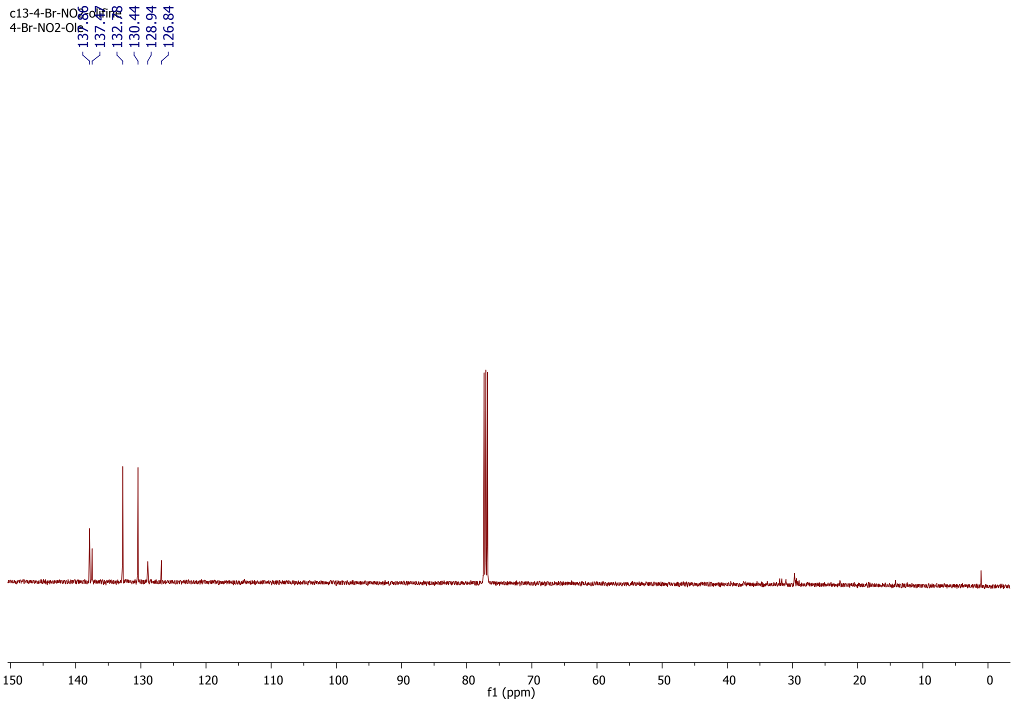

DEPT NMR of (E)-1-bromo-4-(2-nitrovinyl)benzene (2o)

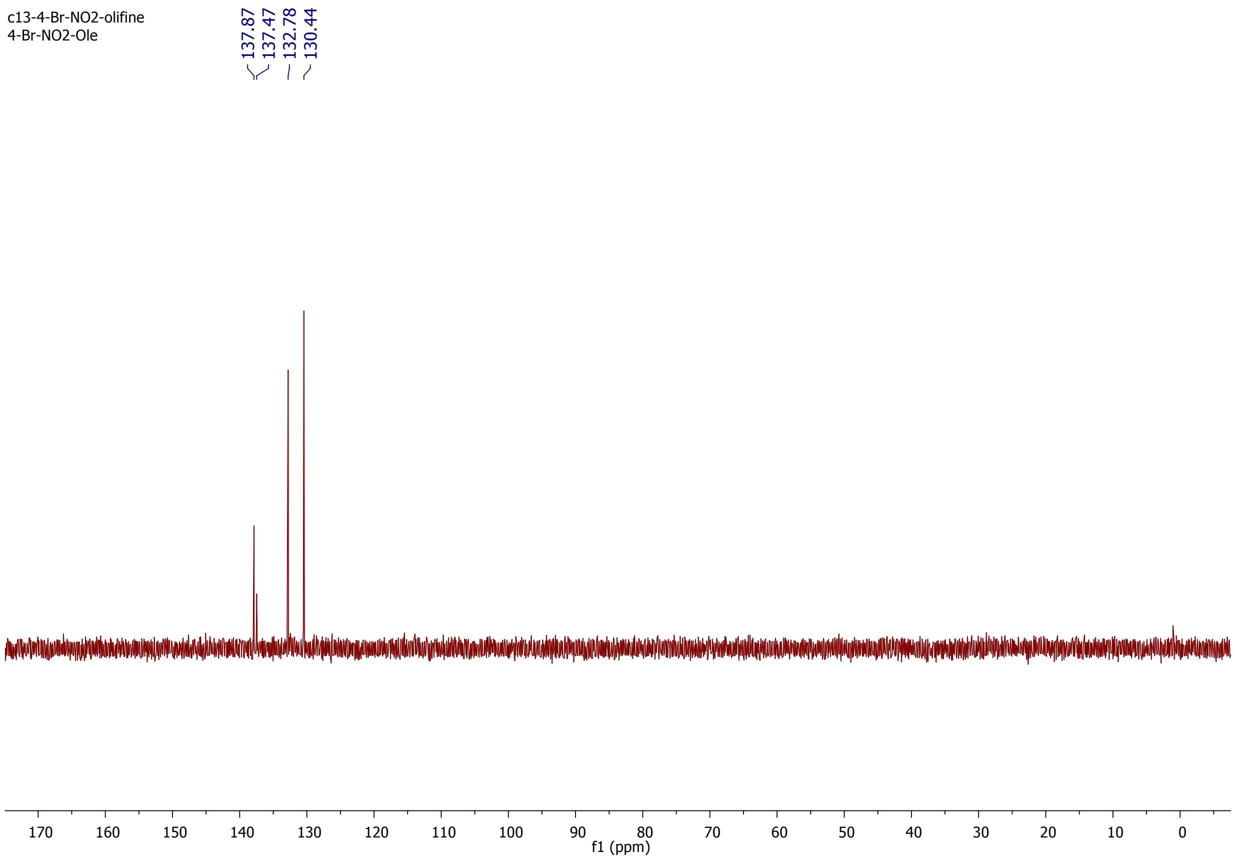

GC-MS of (E)-1-bromo-4-(2-nitrovinyl)benzene (2o)

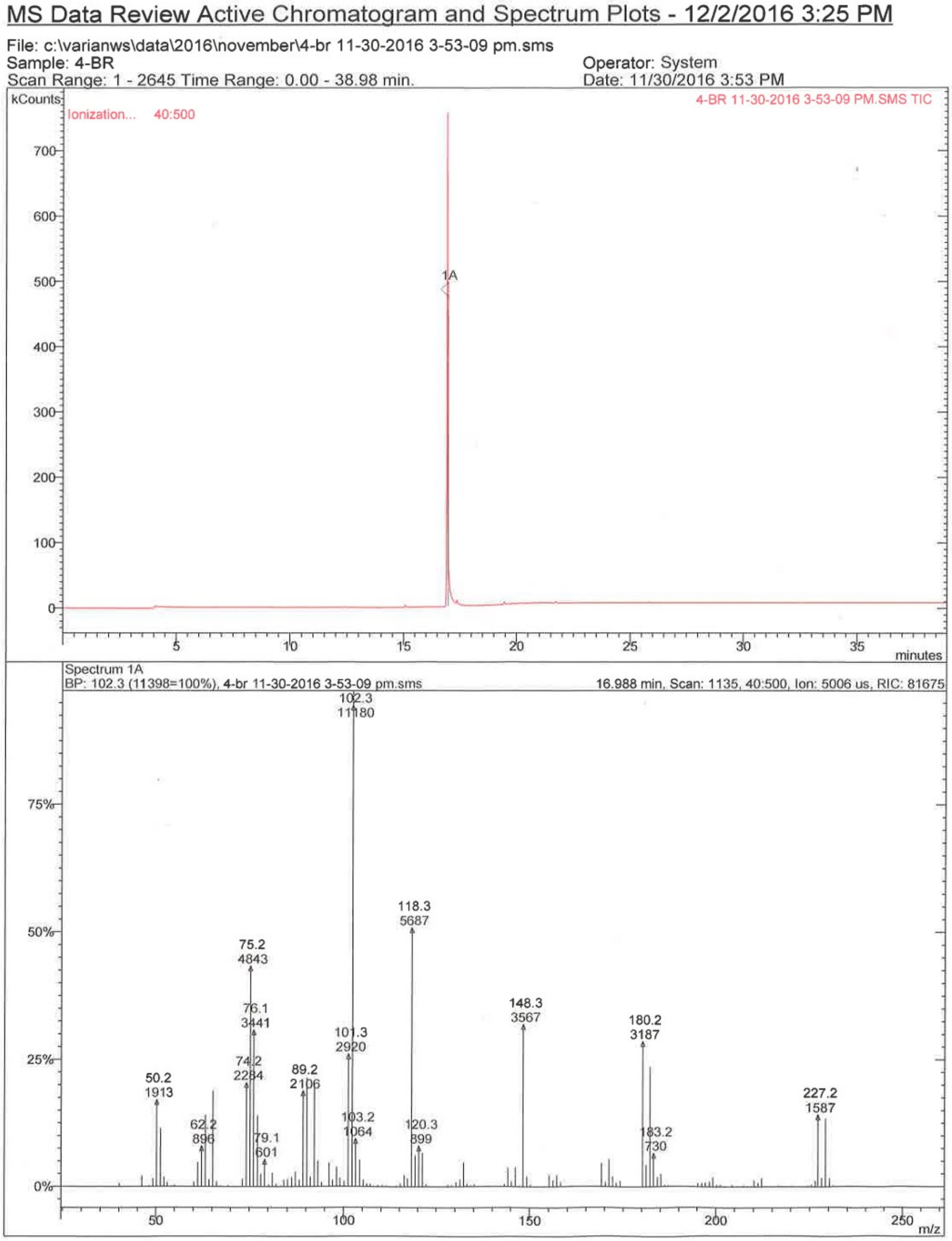

^{1}H NMR of (E)-1-bromo-3-(2-nitrovinyl)benzene (2p)[16]

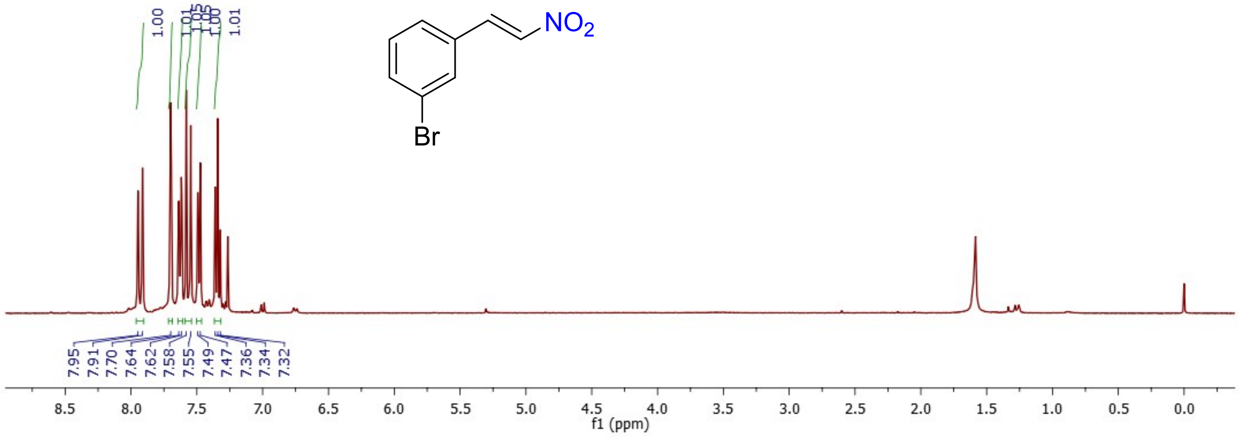

^{13}C NMR of (E)-1-bromo-3-(2-nitrovinyl)benzene (2p)

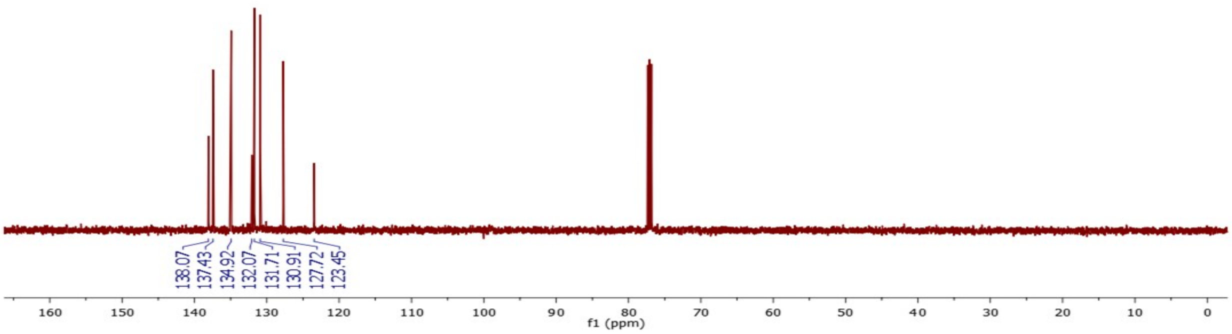

GC-MS of (E)-1-bromo-3-(2-nitrovinyl)benzene (2p)

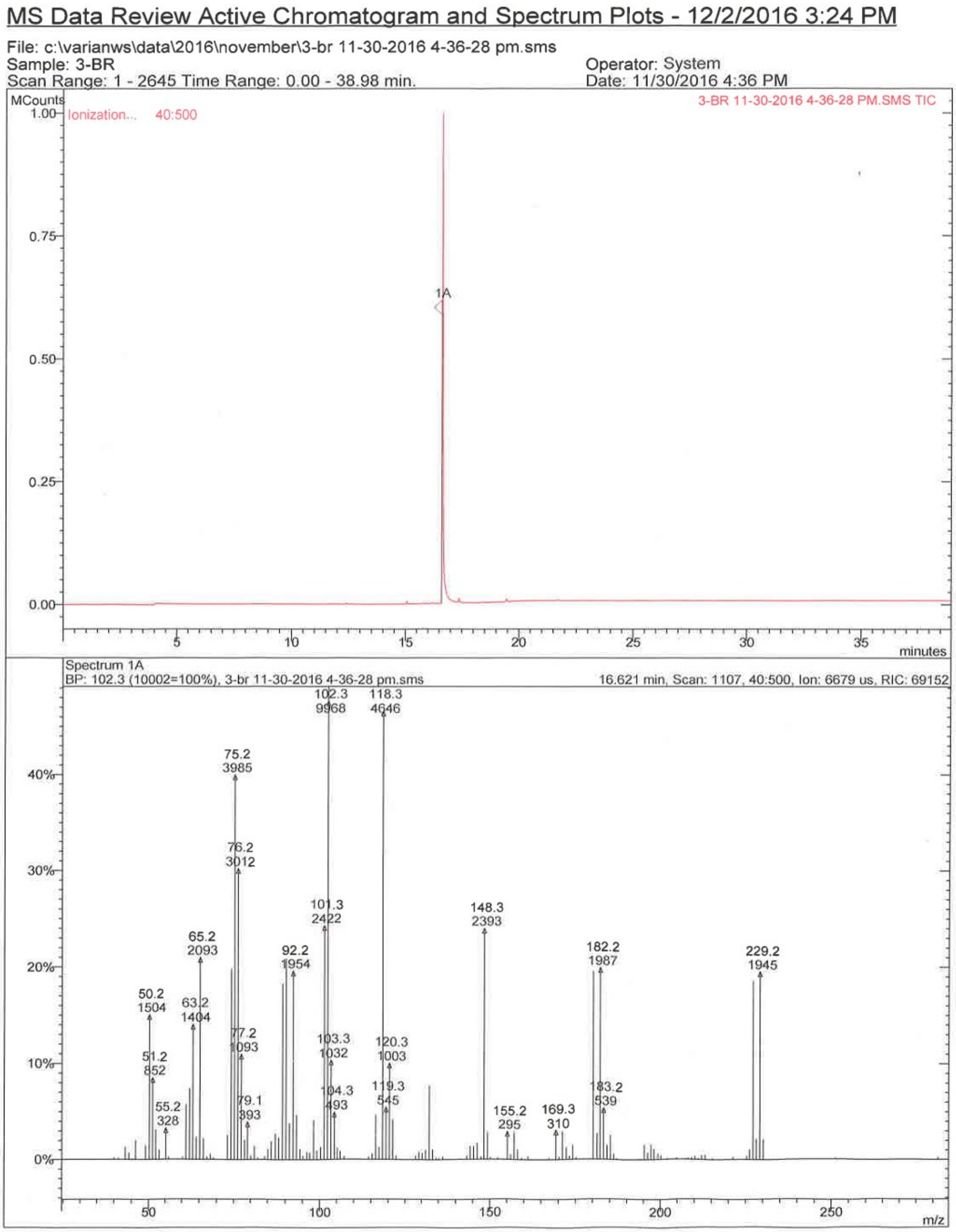

^{1}H NMR of (E)-1-(2-nitrovinyl)-4-(trifluoromethyl)benzene (2q)[12]

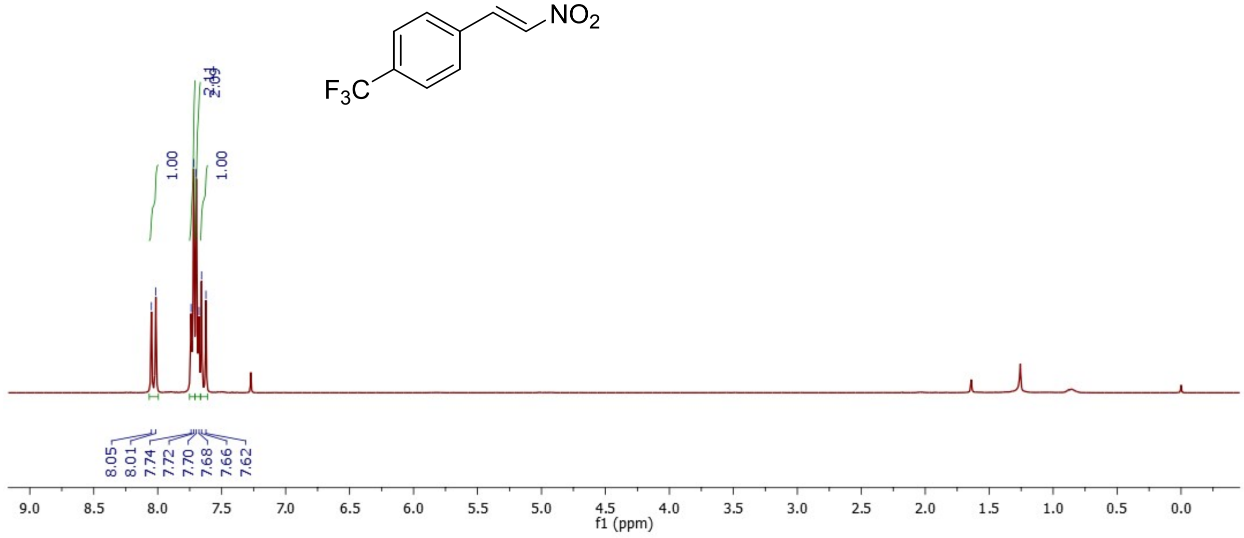

^{13}C NMR of (E)-1-(2-nitrovinyl)-4-(trifluoromethyl)benzene (2q)

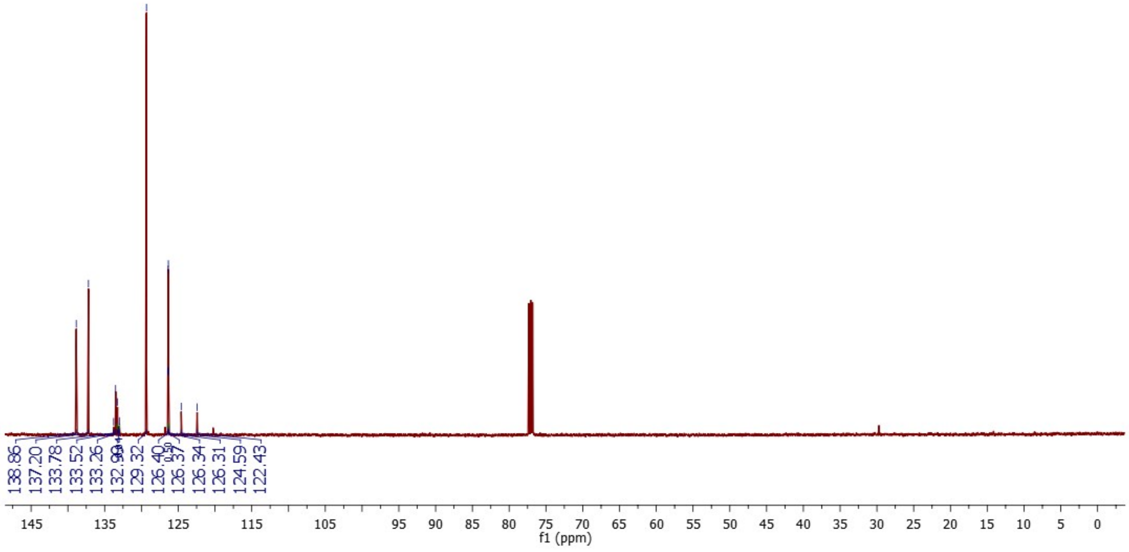

DEPT NMR of (E)-1-(2-nitrovinyl)-4-(trifluoromethyl)benzene (2q)

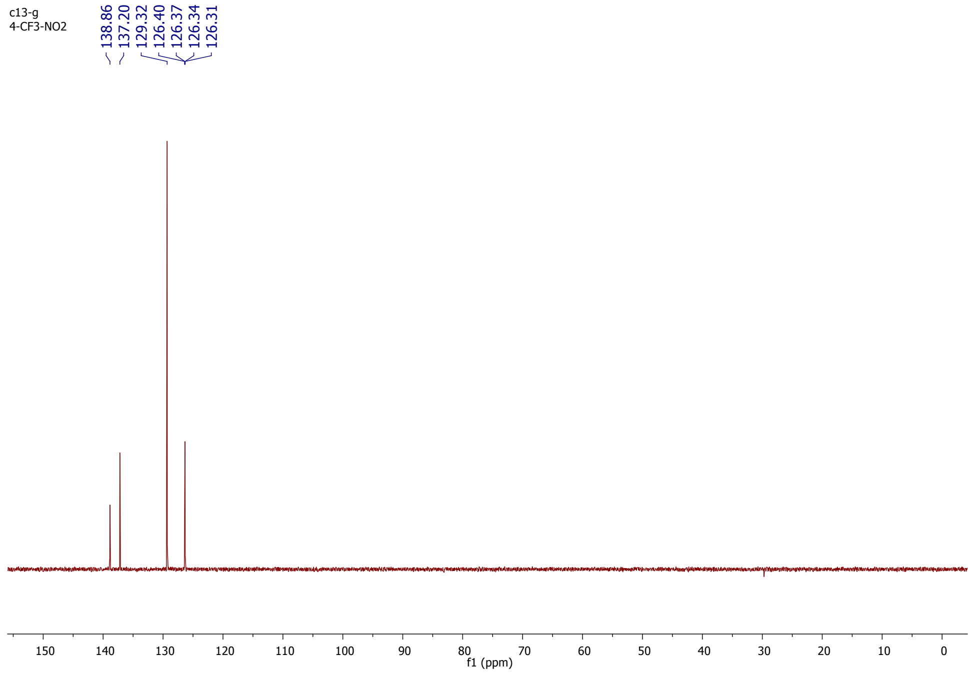

^{1}H NMR of (E)-1-(2-nitrovinyl)-4-(trifluoromethoxy)benzene (2r)[11]

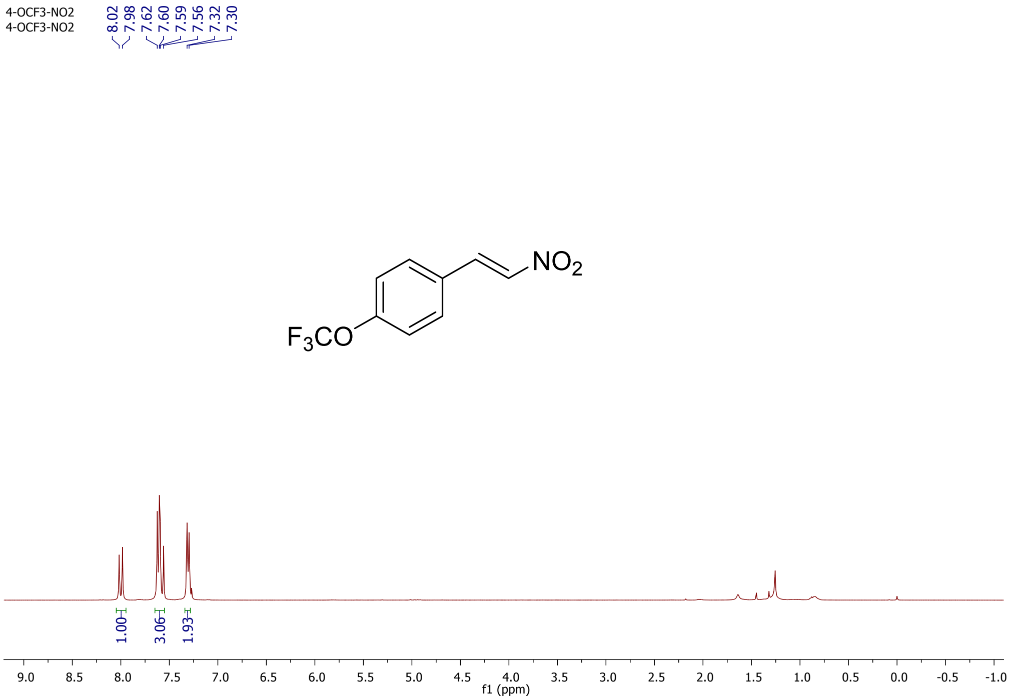

^{13}C NMR of (E)-1-(2-nitrovinyl)-4-(trifluoromethoxy)benzene (2r)

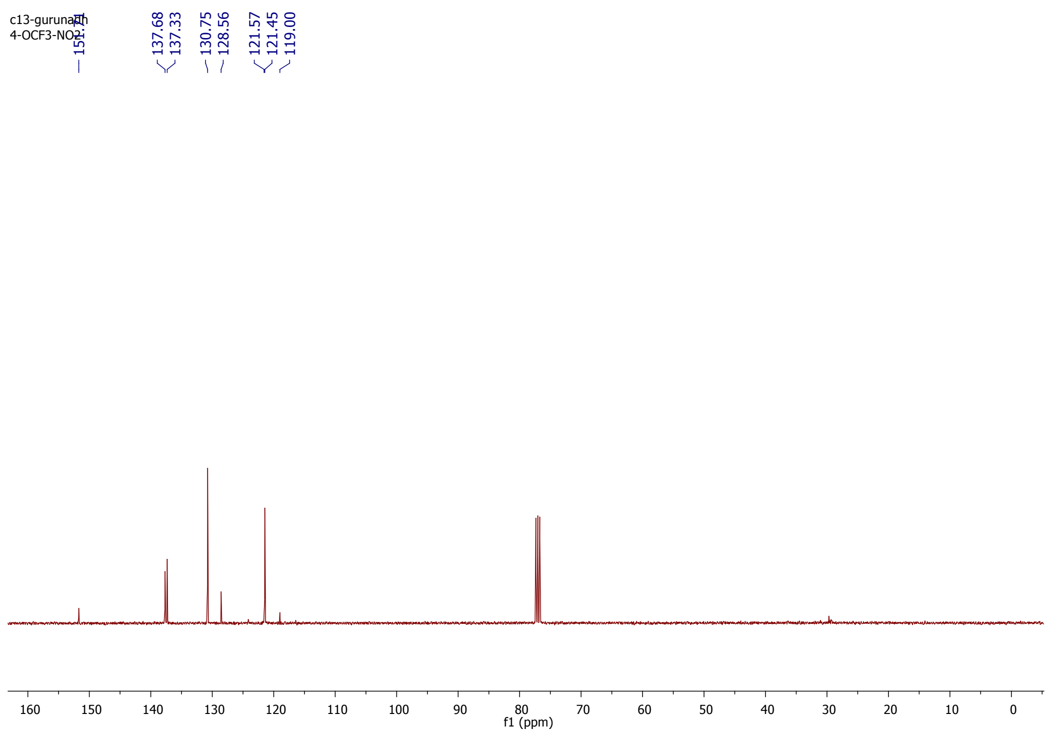

DEPT NMR of (E)-1-(2-nitrovinyl)-4-(trifluoromethoxy)benzene (2r)

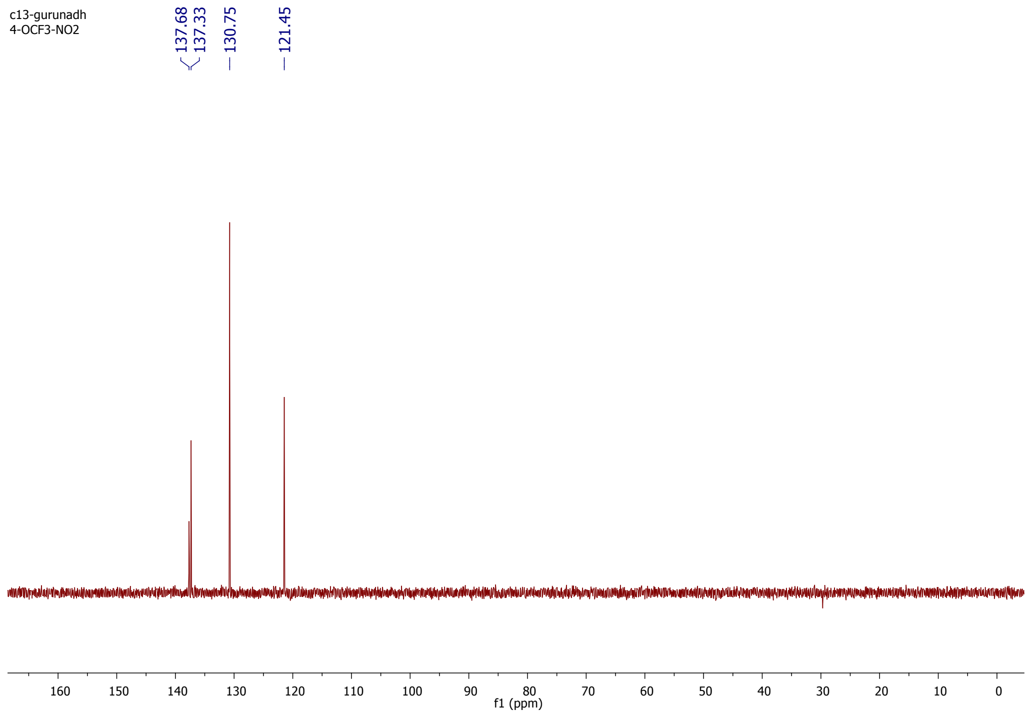

GC-MS of (E)-1-(2-nitrovinyl)-4-(trifluoromethoxy)benzene (2r)

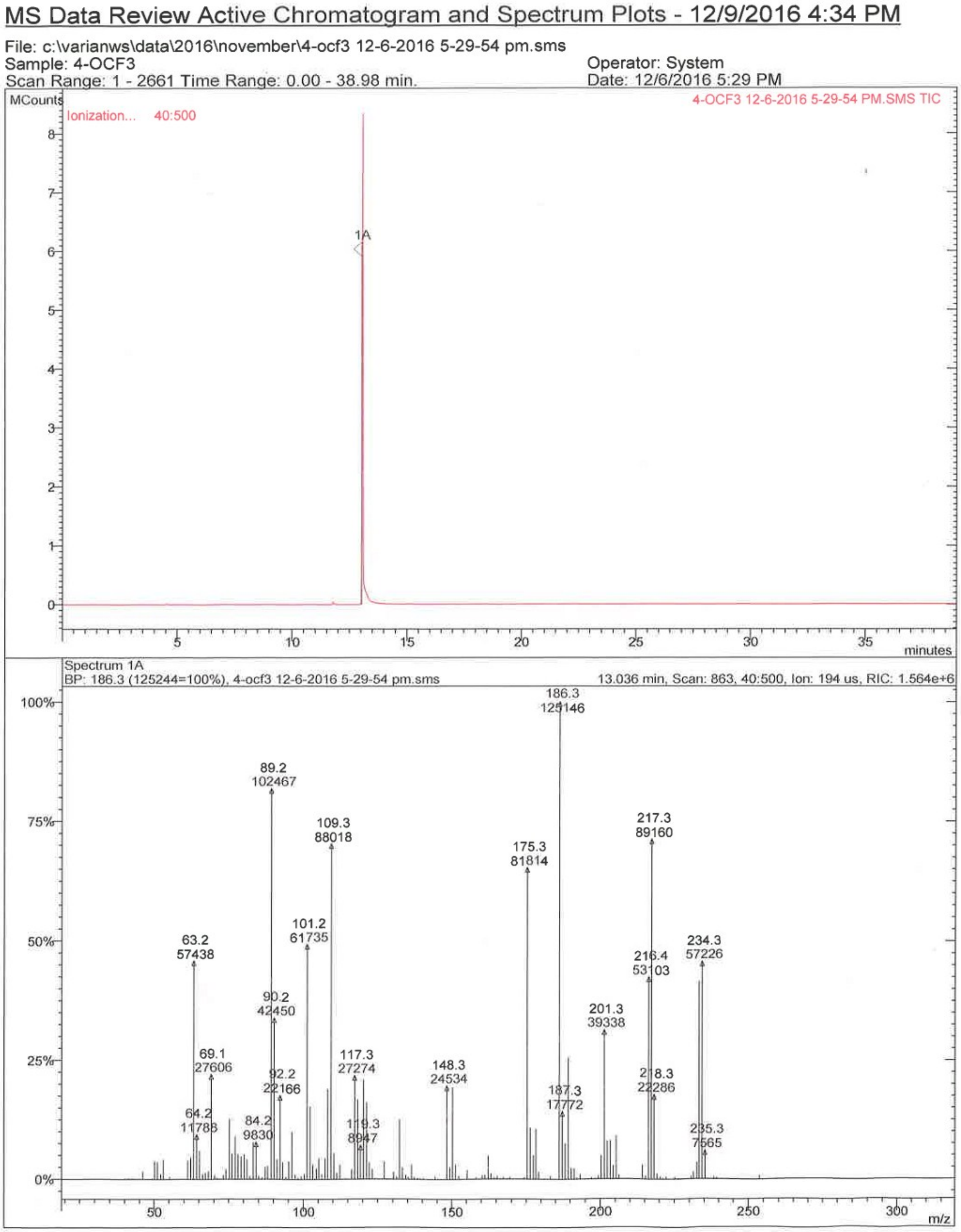

^{1}H NMR of (E)-1-(2-nitrovinyl)-3,5-bis(trifluoromethyl)benzene (2s)[12]

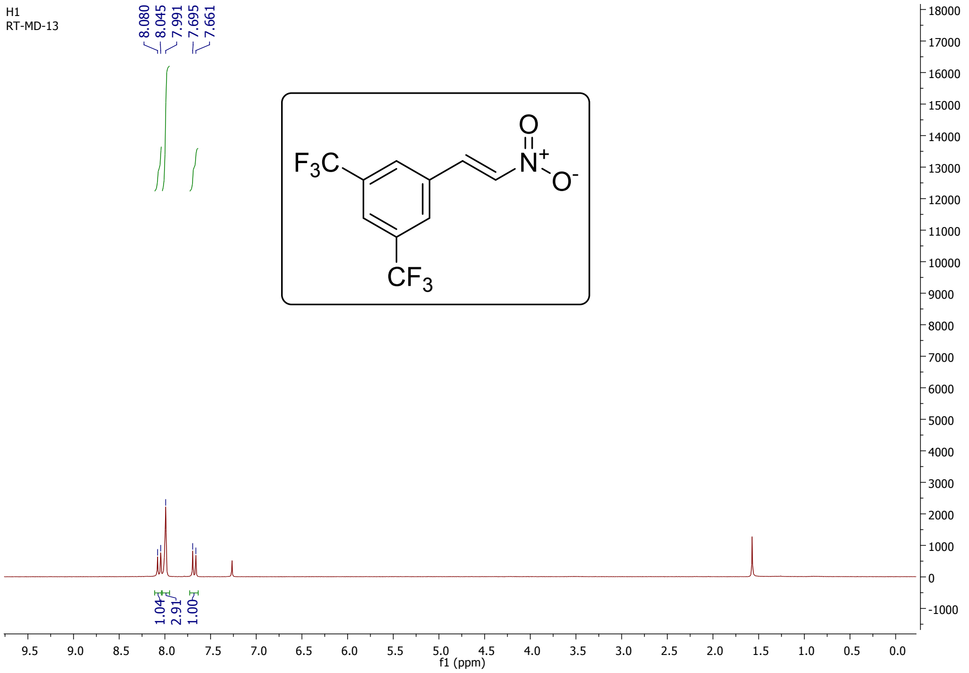

[13]C of (E)-1-(2-nitrovinyl)-3,5-bis(trifluoromethyl)benzene (2s)

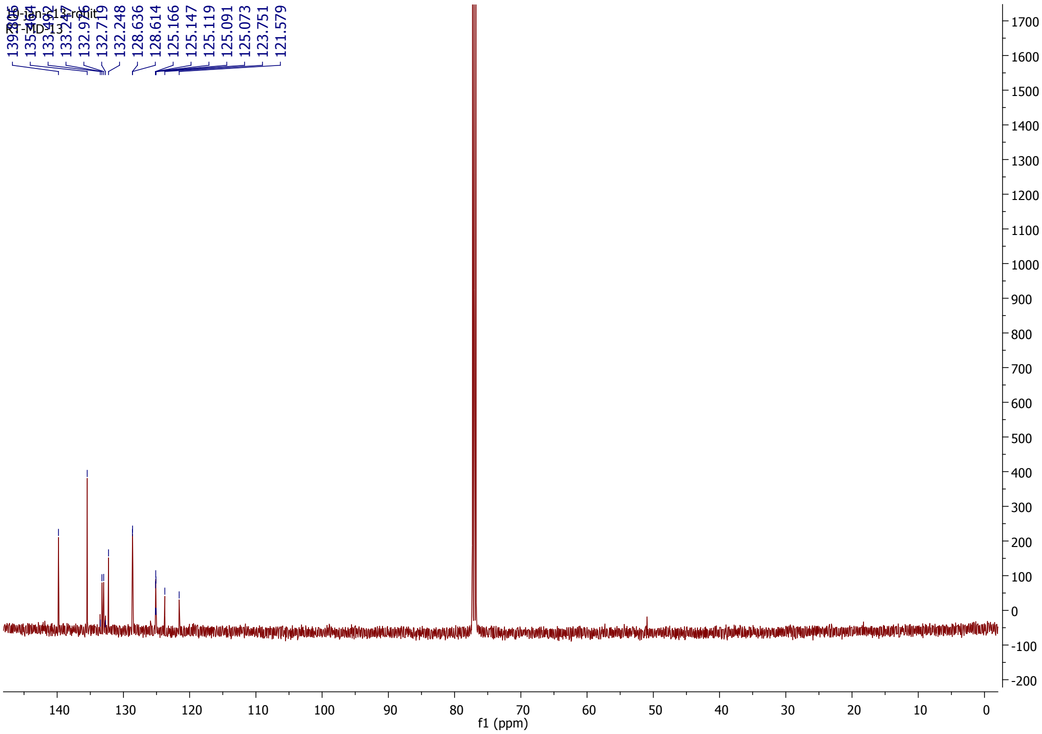

GC-MS Spectra of (E)-1-(2-nitrovinyl)-3,5-bis(trifluoromethyl)benzene (2s)

**Sample Name**	MD-13	**Position**	9	**Instrument Name**	GCMS	**User Name**	manager
**Inj Vol**	0	**InjPosition**		**SampleType**	Sample	**IRM Calibration Status**	Not Applicable
**Data Filename**	04-06-18AMIT09.D	**ACQ Method**	AMIT.M	**Comment**		**Acquired Time**	6/5/2018 7:45:46 PM

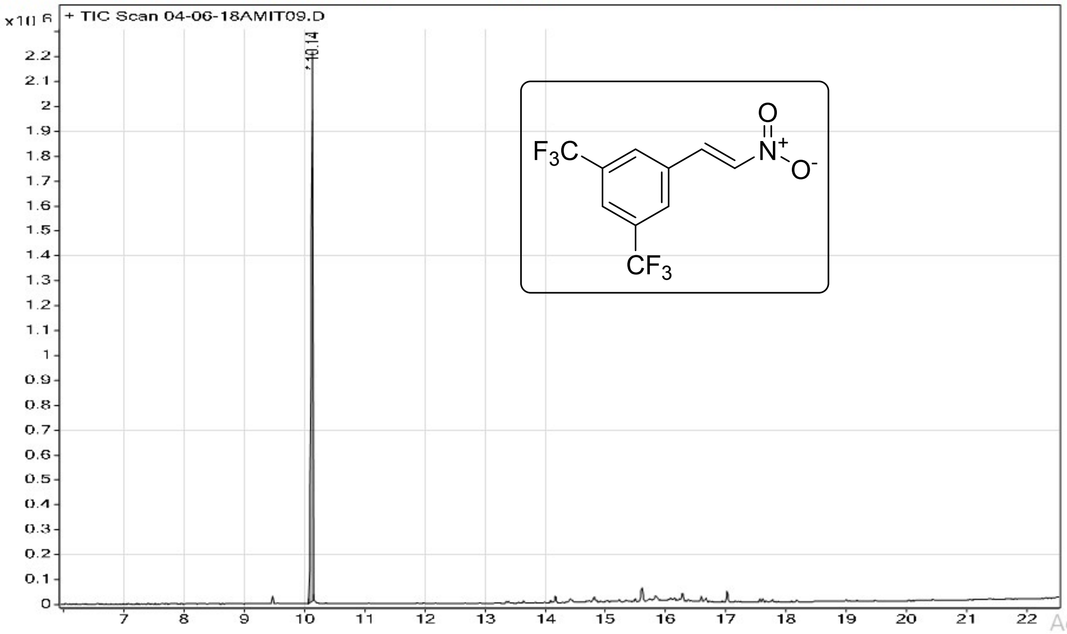

Activat

**Sample Name**	MD-13	**Position**	9	**Instrument Name**	GCMS	**User Name**	manager
**Inj Vol**	0	**InjPosition**		**SampleType**	Sample	**IRM Calibration Status**	Not Applicable
**Data Filename**	04-06-18AMIT09.D	**ACQ Method**	AMIT.M	**Comment**		**Acquired Time**	6/5/2018 7:45:46 PM

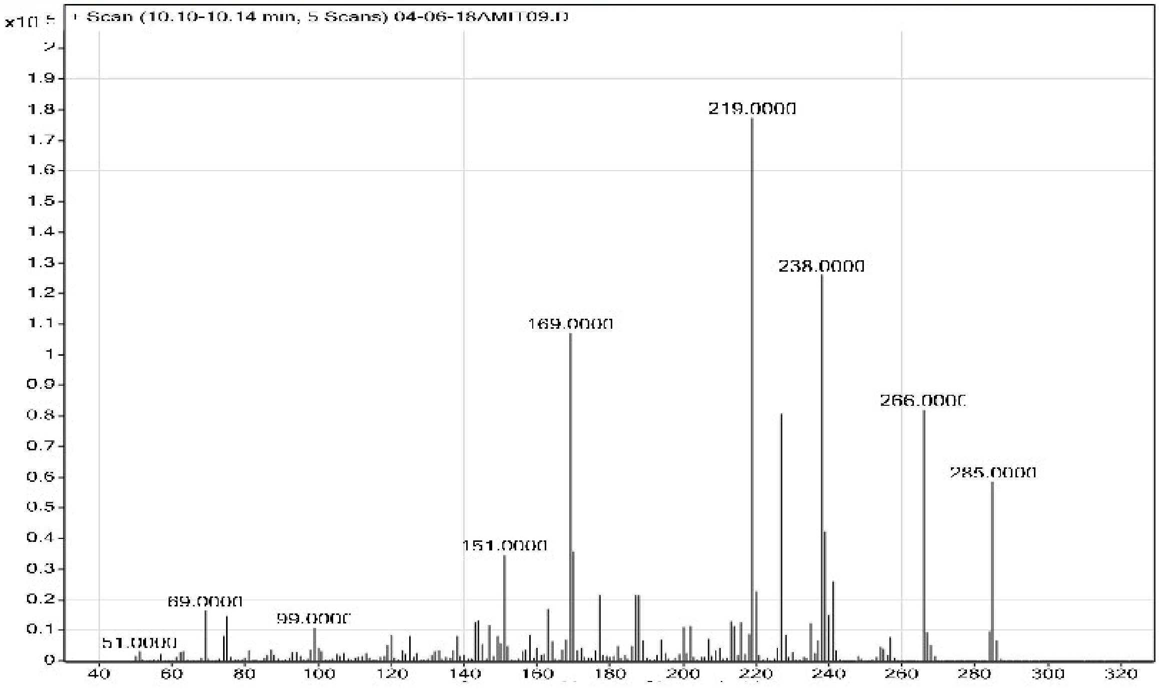

A

¹H NMR of (E)-4-(2-nitrovinyl)benzonitrile (2t)[13]

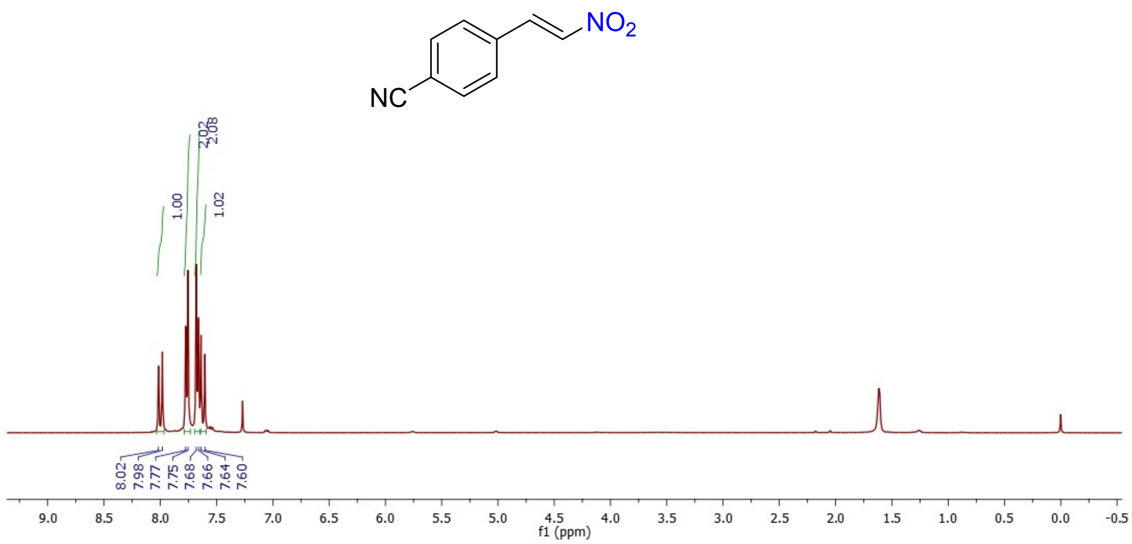

¹³C NMR of (E)-4-(2-nitrovinyl)benzonitrile (2t)

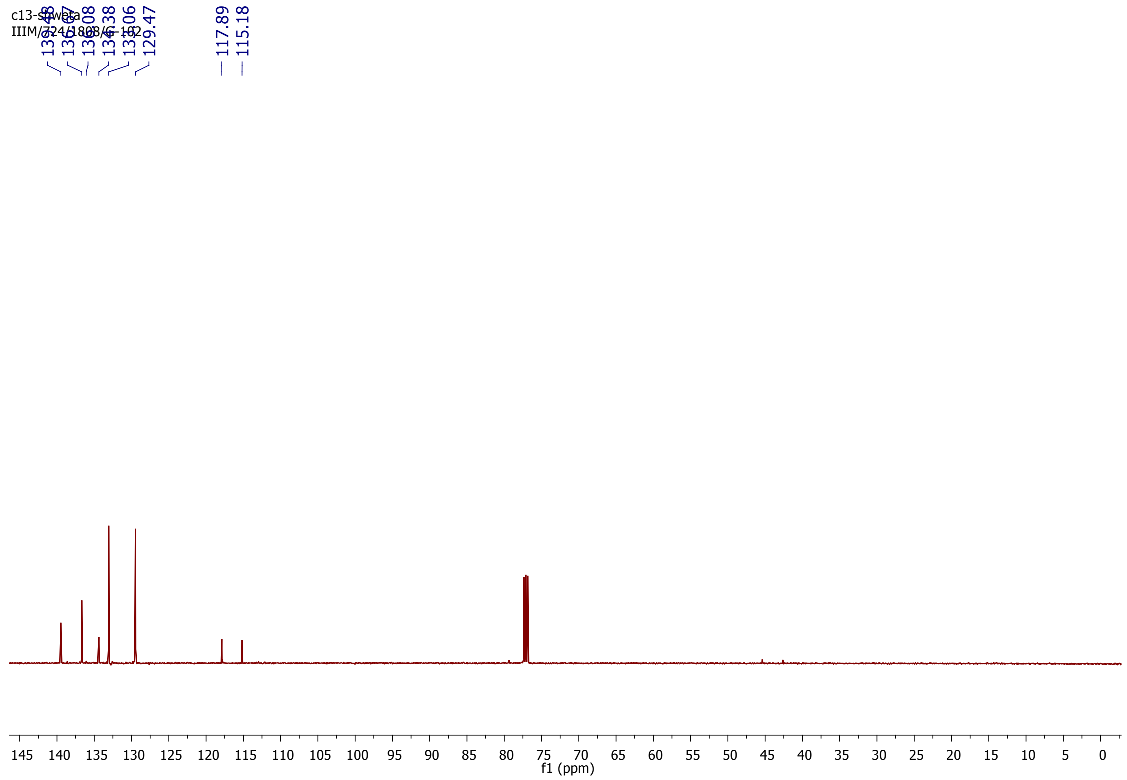

DEPT NMR of (E)-4-(2-nitrovinyl)benzonitrile (2t)

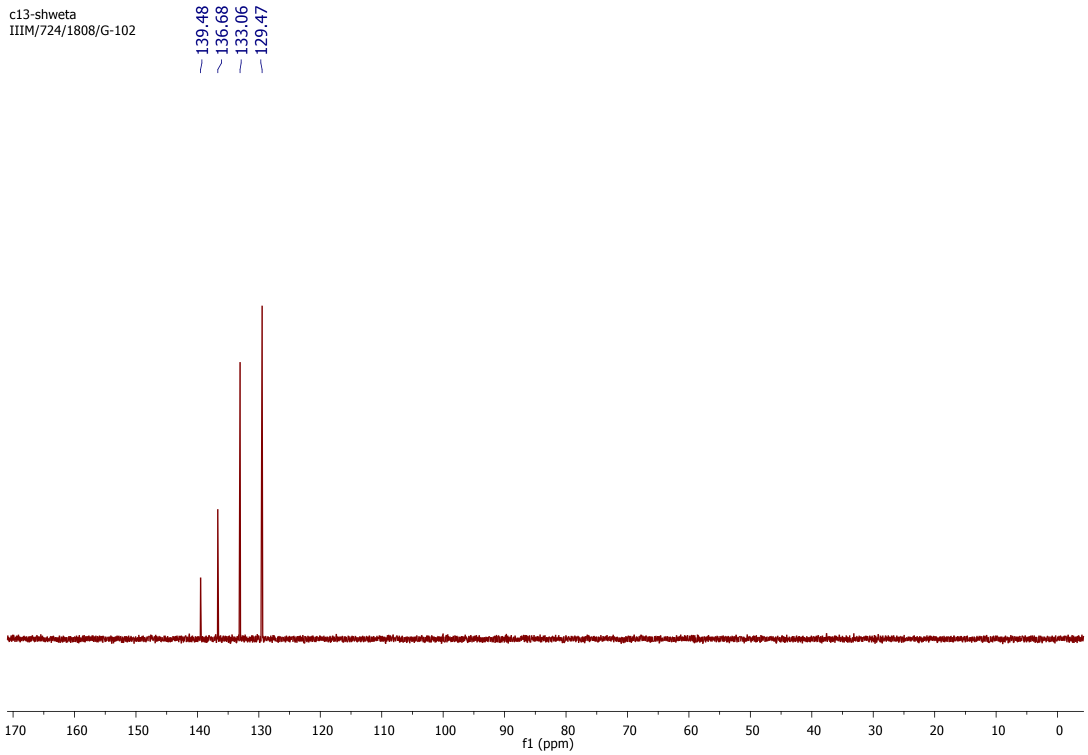

GC-MS of (E)-4-(2-nitrovinyl)benzonitrile (2t)

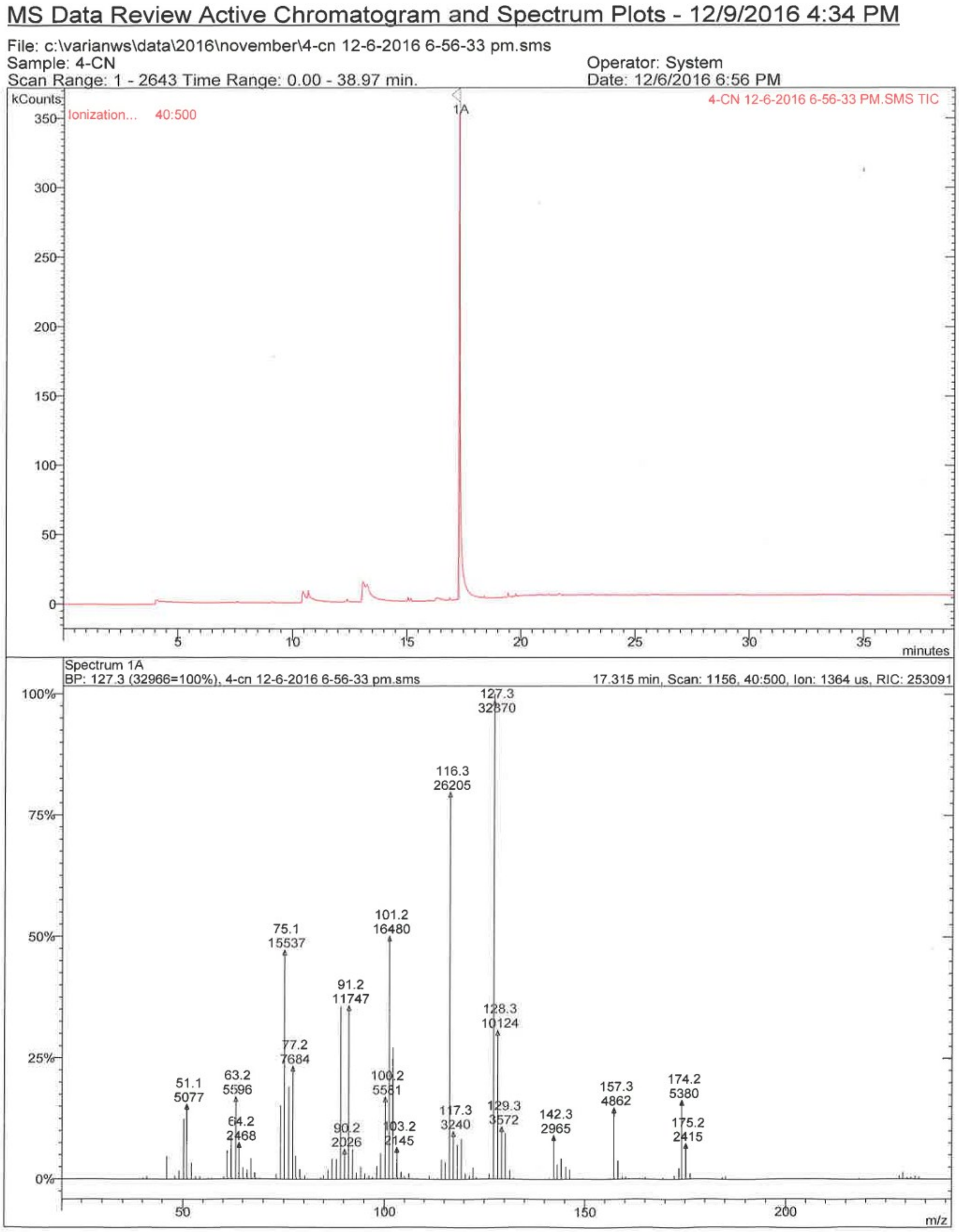

^{1}H NMR OF (E)-4-(2-nitrovinyl)phenyl acetate(2u)[14]

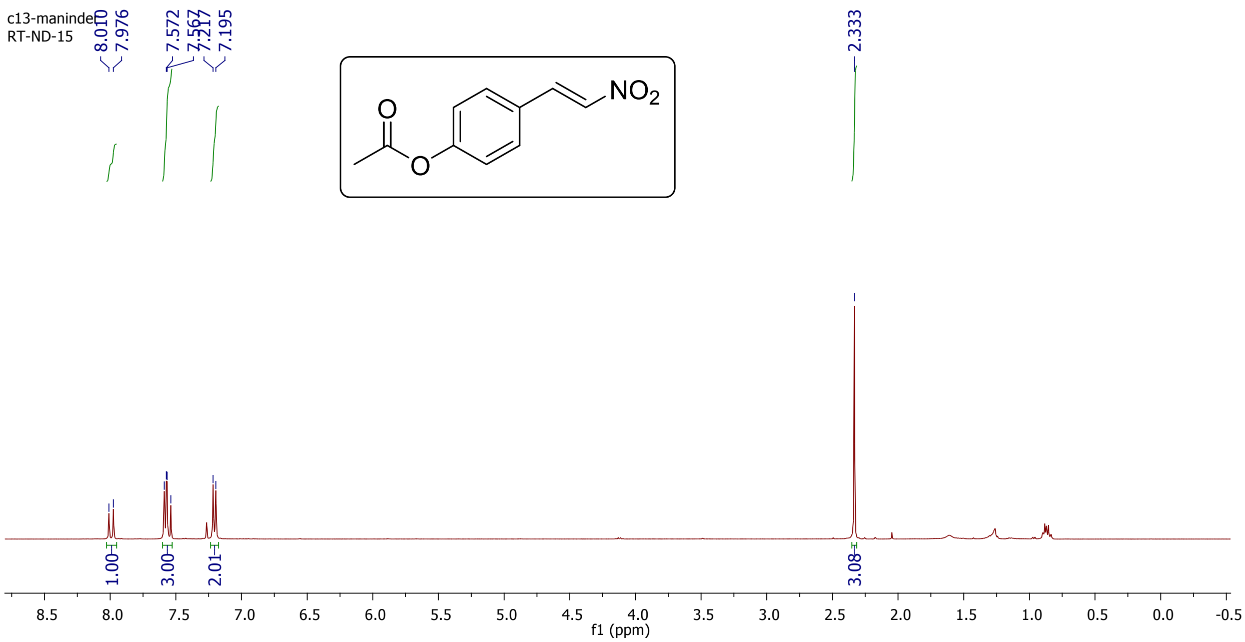

^{13}C NMR of (E)-4-(2-nitrovinyl)phenyl acetate(2u)

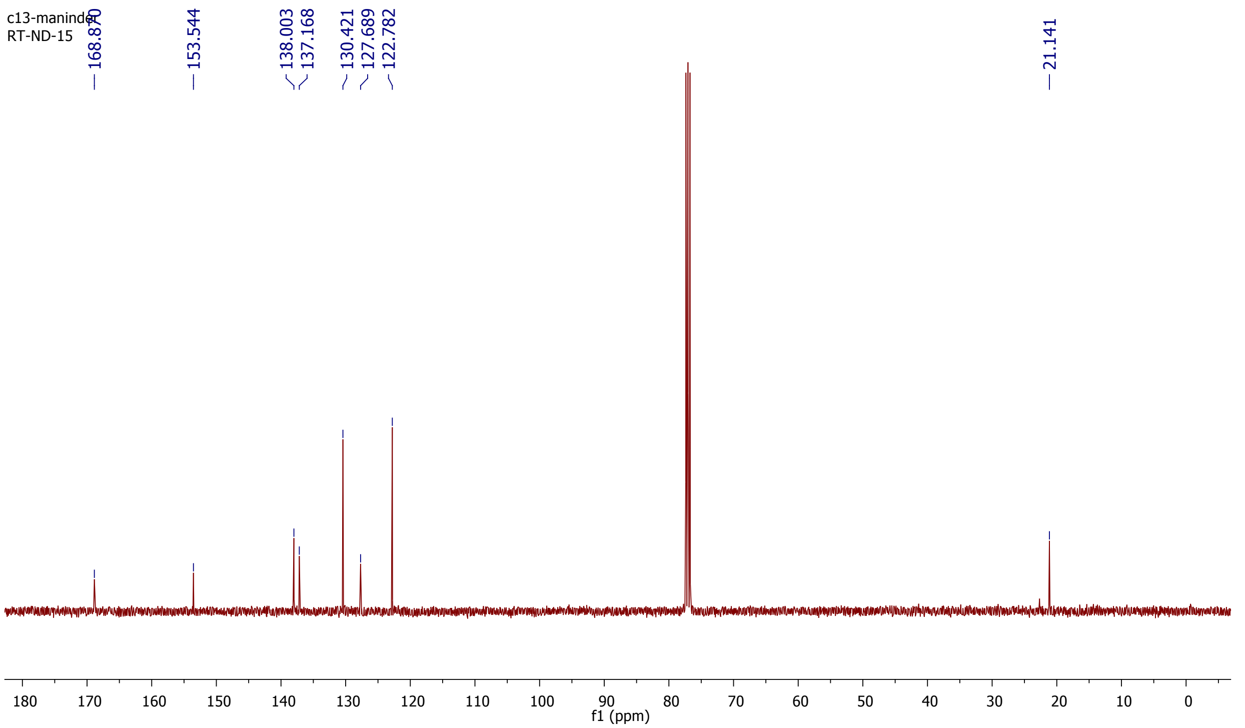

DEPT NMR of (E)-4-(2-nitrovinyl)phenyl acetate(2u)

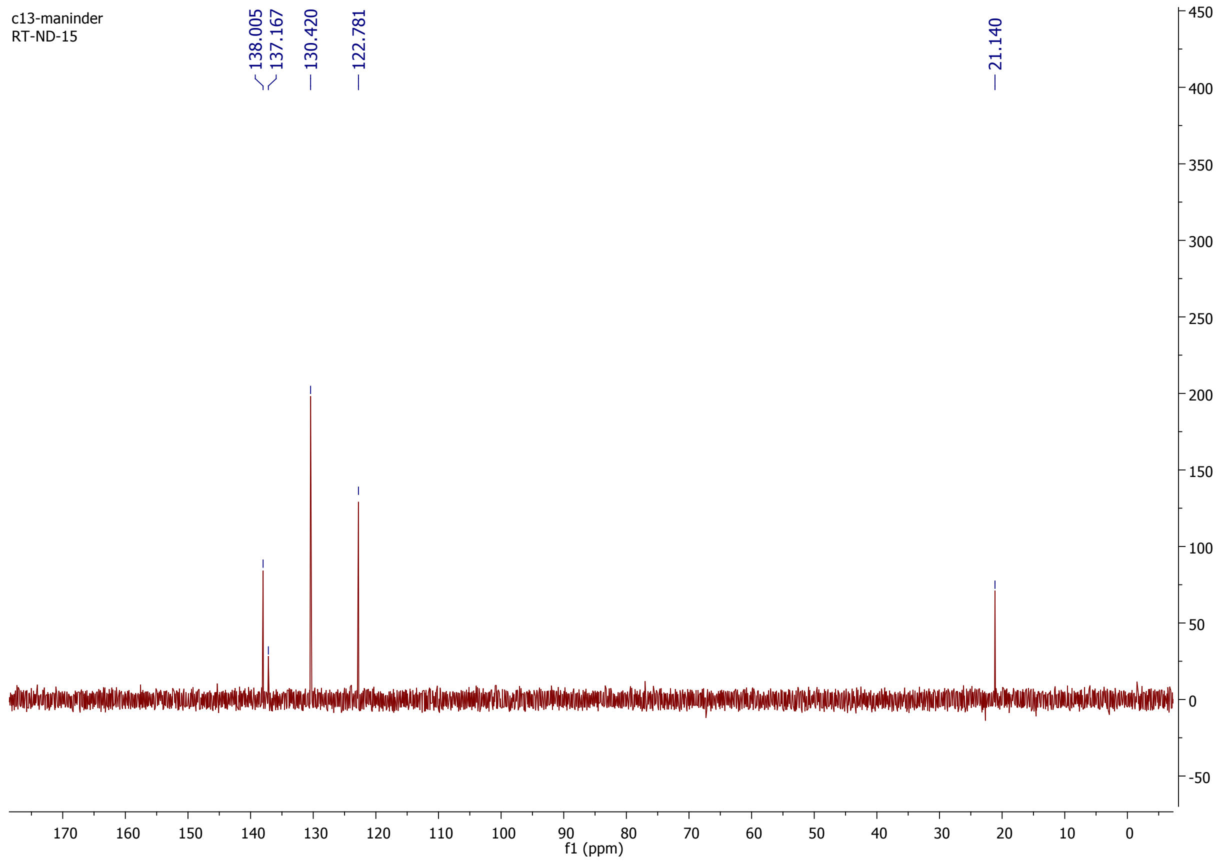

GC-MS spectra of (E)-4-(2-nitrovinyl)phenyl acetate(2u)

**Sample Name**	MD-15	**Position**	11	**Instrument Name**	GCMS	**User Name**	manager
**Inj Vol**	0	**InjPosition**		**SampleType**	Sample	**IRM Calibration Status**	Not Applicable
**Data Filename**	04-06-18AMIT11.D	**ACQ Method**	AMIT.M	**Comment**		**Acquired Time**	6/5/2018 7:46:02 PM

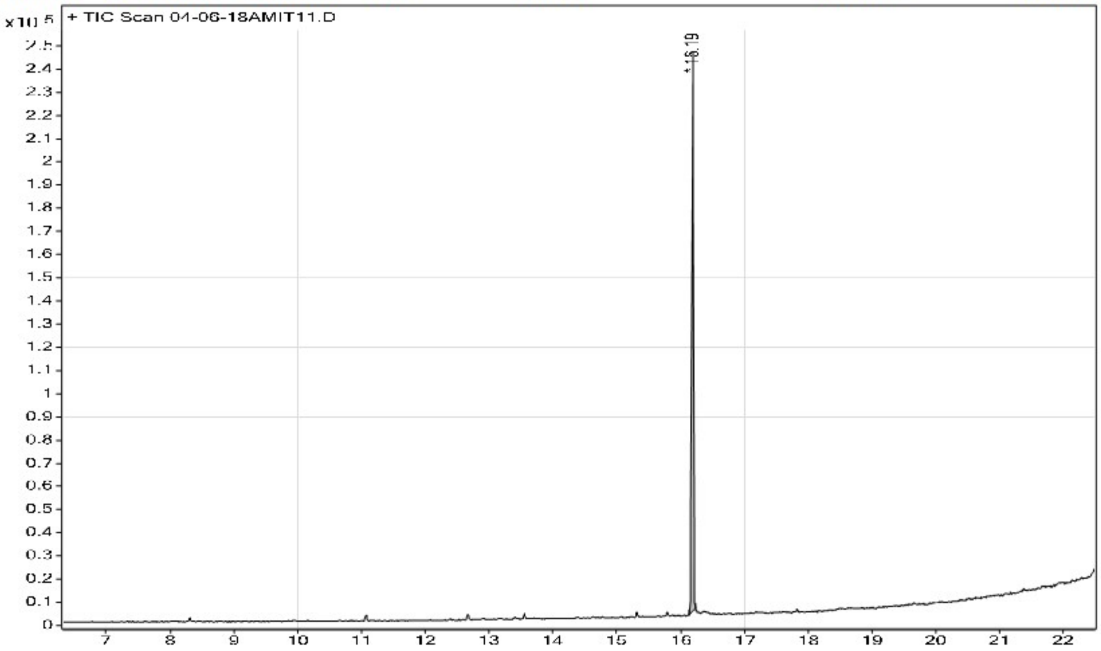

**Sample Name**	MD-15	**Position**	11	**Instrument Name**	GCMS	**User Name**	manager
**Inj Vol**	0	**InjPosition**		**SampleType**	Sample	**IRM Calibration Status**	Not Applicable
**Data Filename**	04-06-18AMIT11.D	**ACQ Method**	AMIT.M	**Comment**		**Acquired Time**	6/5/2018 7:46:02 PM

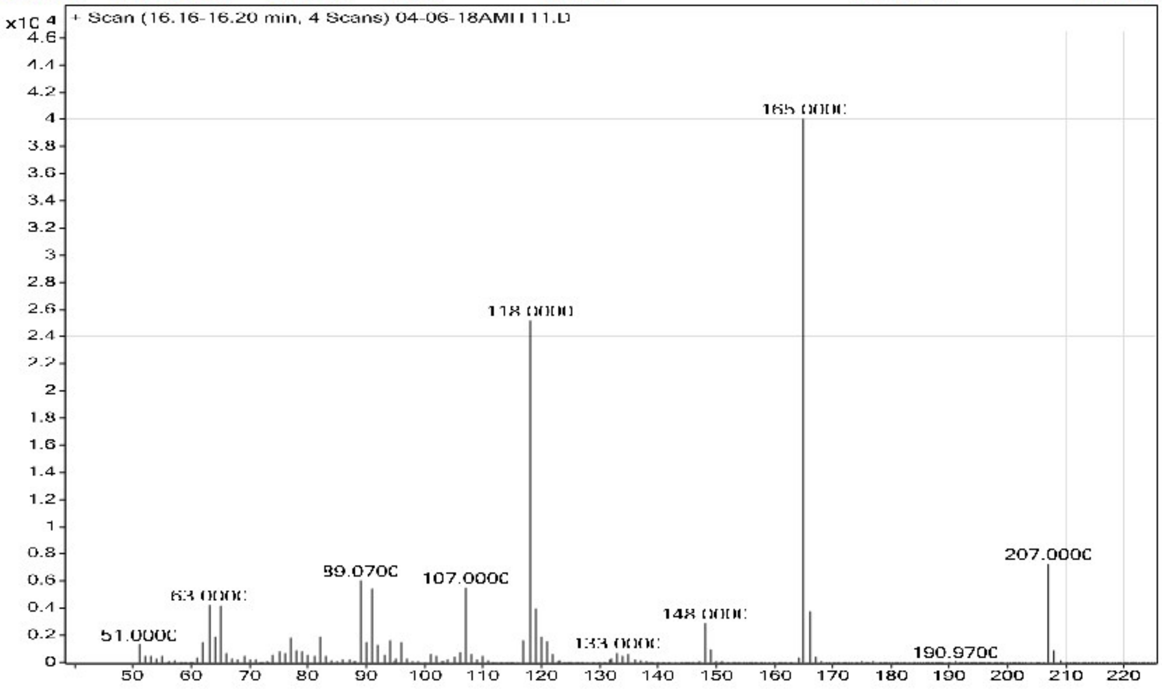

^{1}H NMR of (E)-2-(2-nitrovinyl)naphthalene (2v)[15]

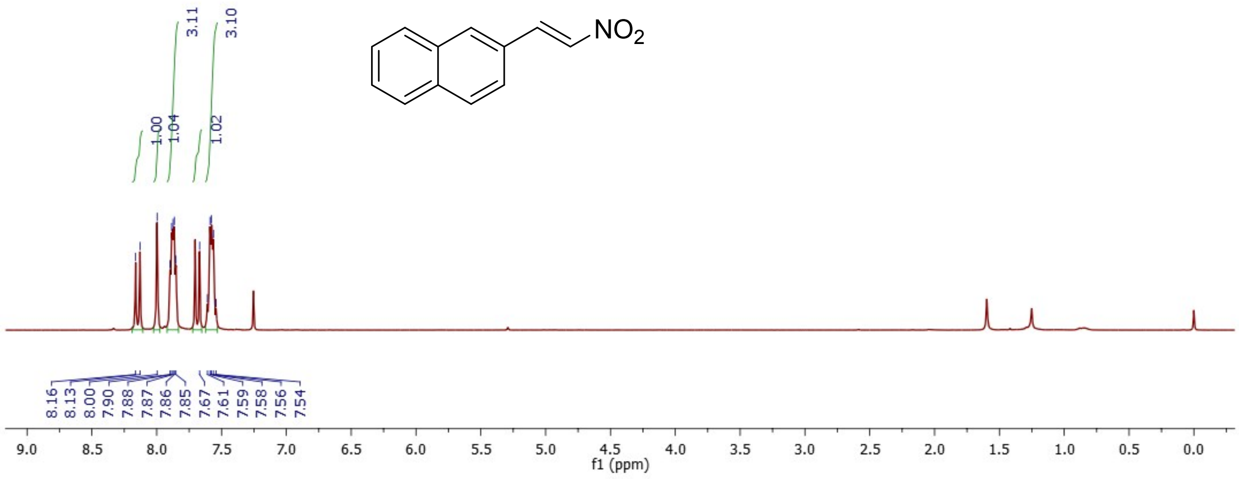

^{13}C NMR of (E)-2-(2-nitrovinyl)naphthalene (2v)

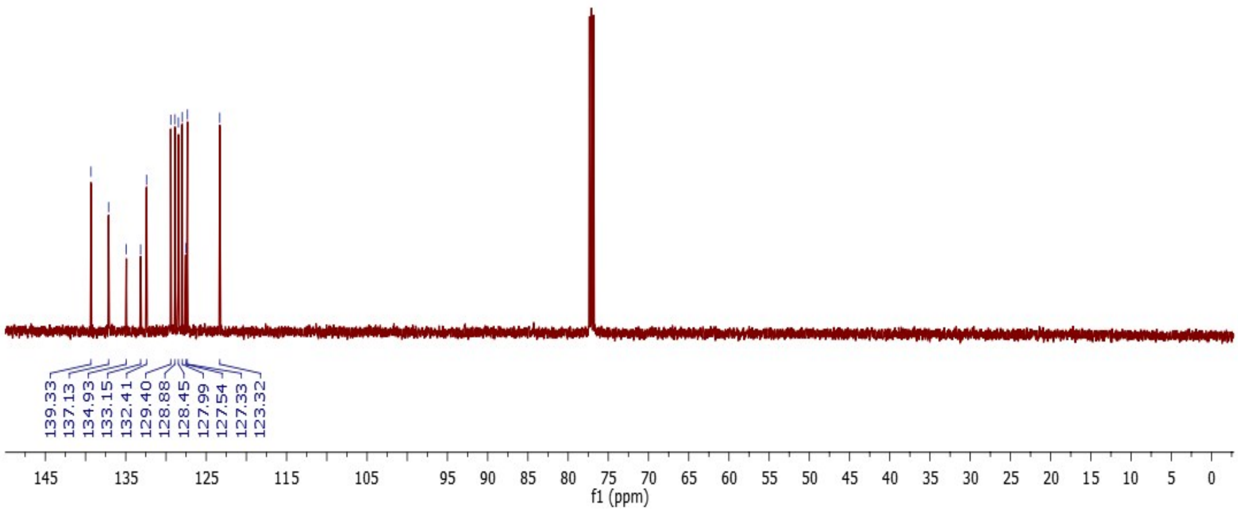

GC-MS of (E)-2-(2-nitrovinyl)naphthalene (2v)

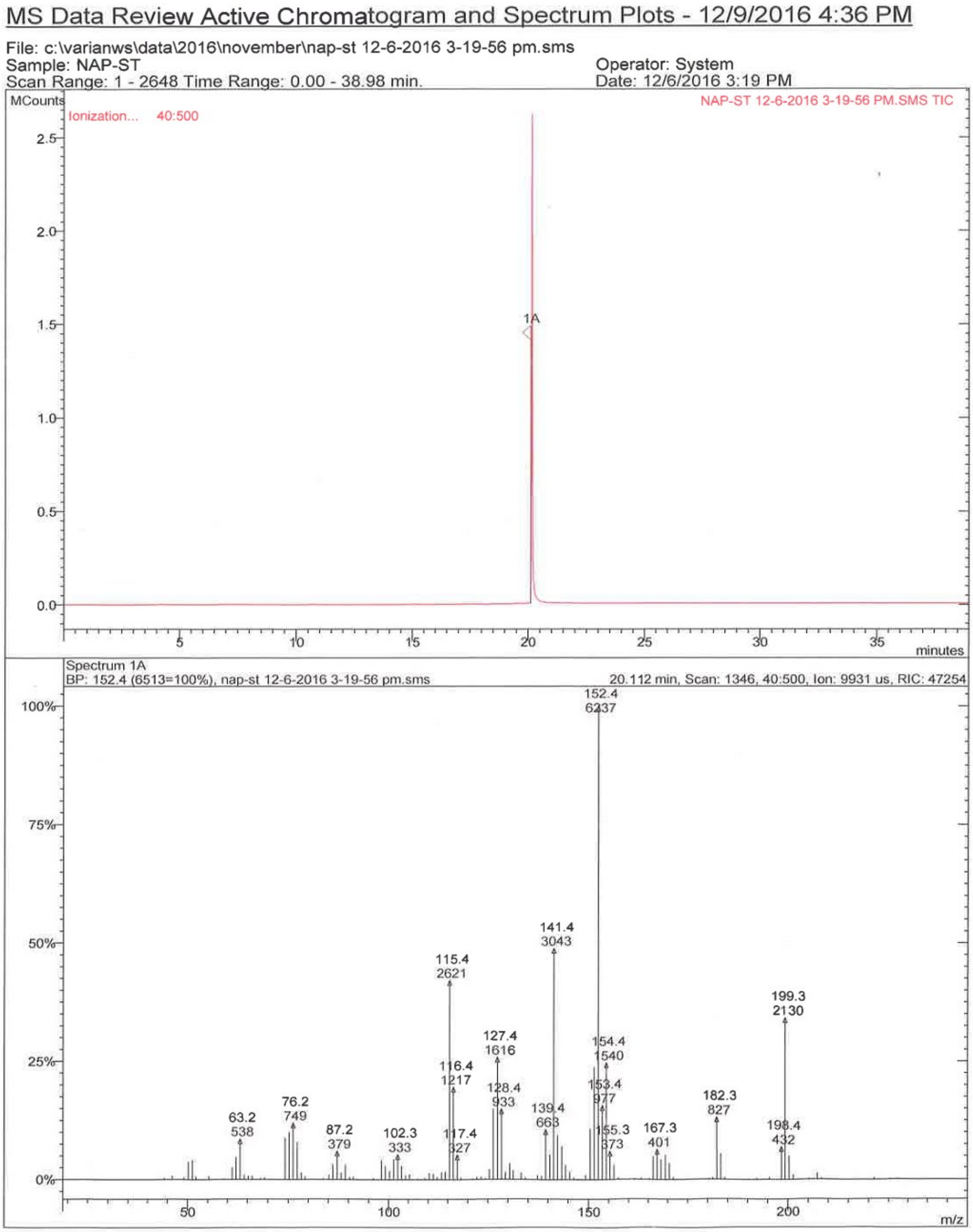

^{1}H NMR of (E)-4-(2-nitrovinyl)-1,1'-biphenyl (2w)[16]

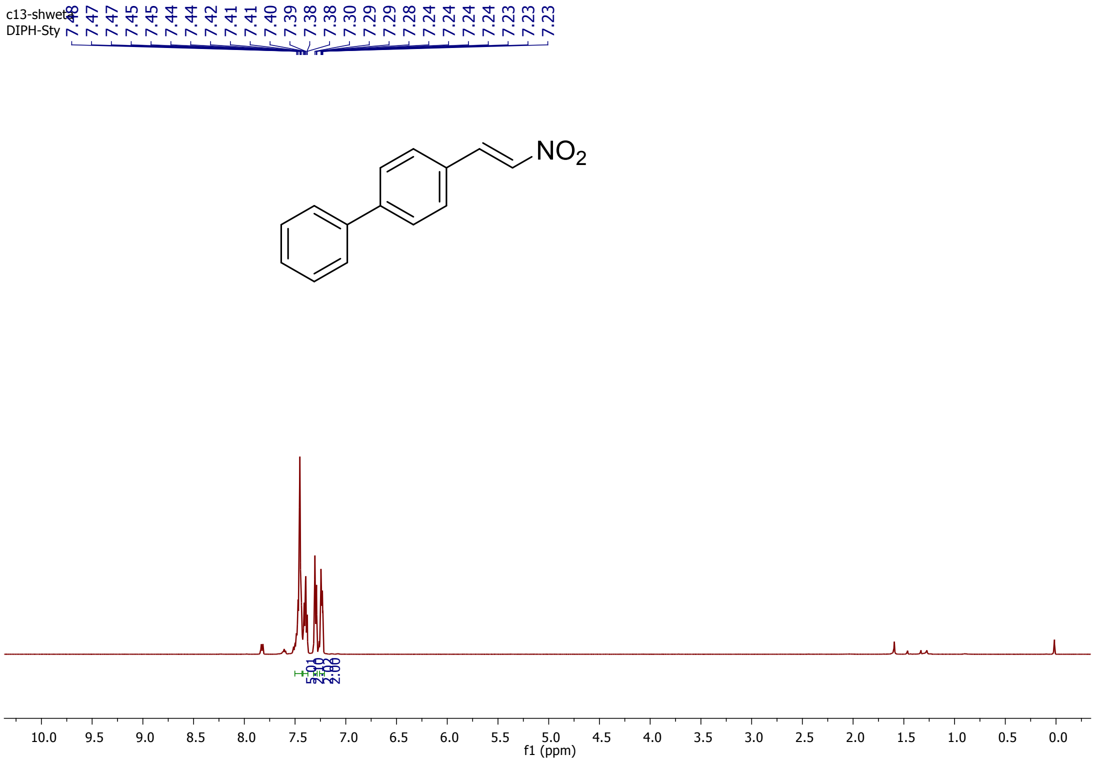

^{13}C NMR of (E)-4-(2-nitrovinyl)-1,1'-biphenyl (2w)

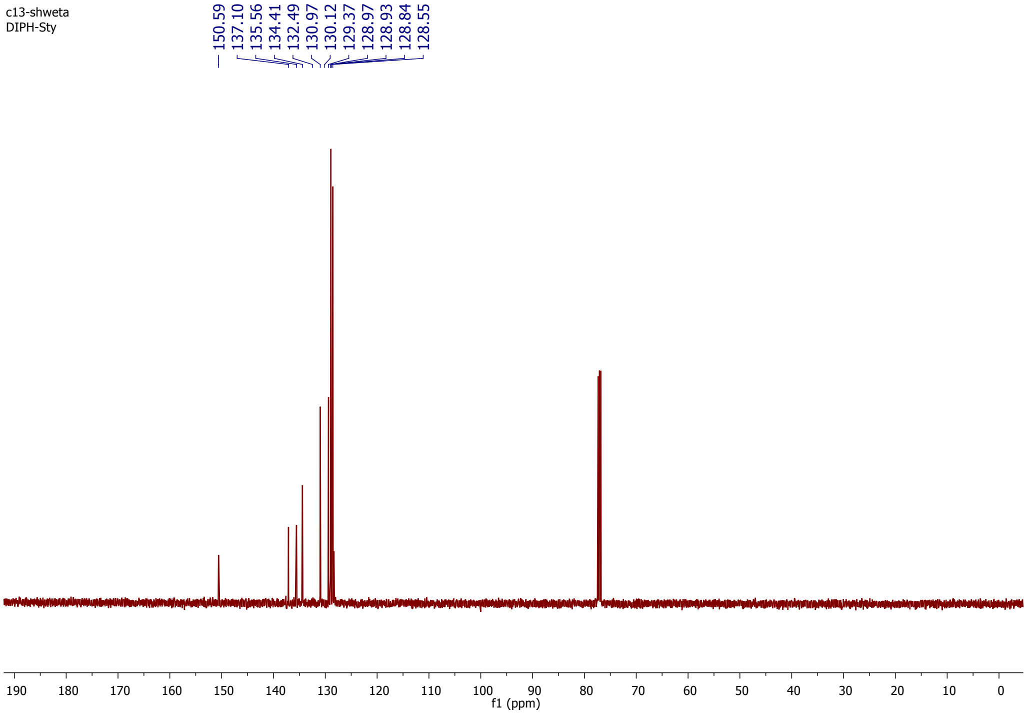

DEPT NMR of (E)-4-(2-nitrovinyl)-1,1'-biphenyl (2w)

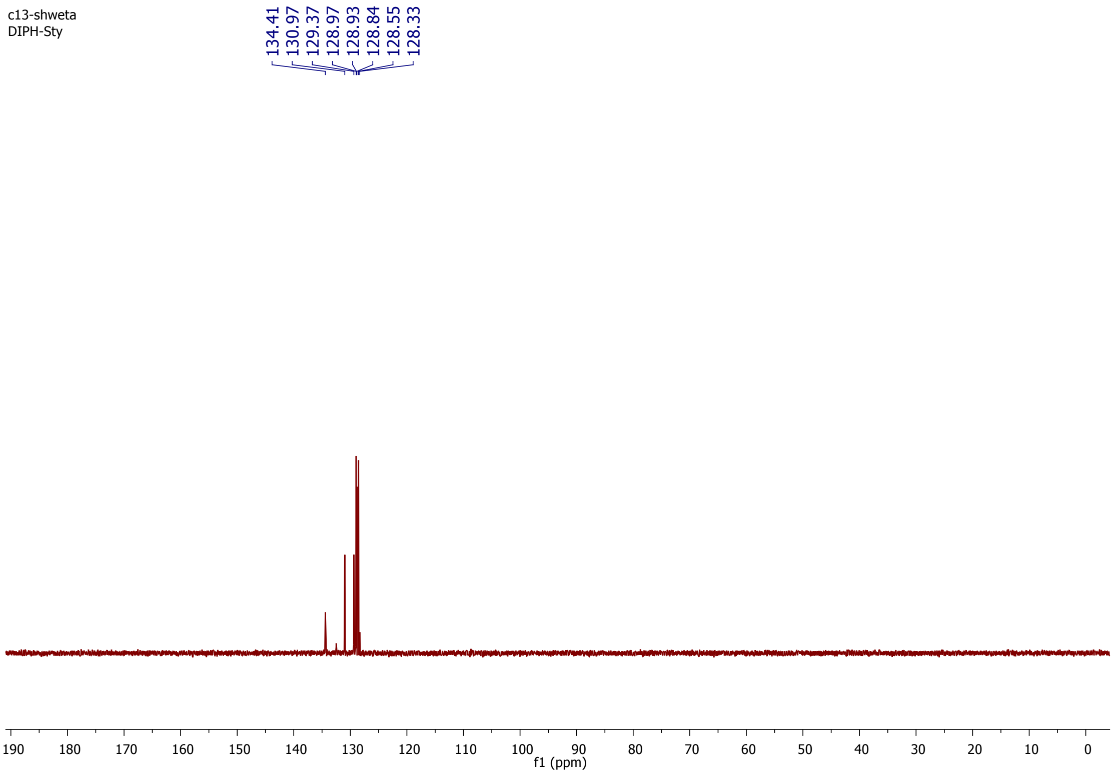

^{1}H NMR of (E/Z)-1-fluoro-4-(1-nitroprop-1-en-2-yl)benzene (2x) [17]

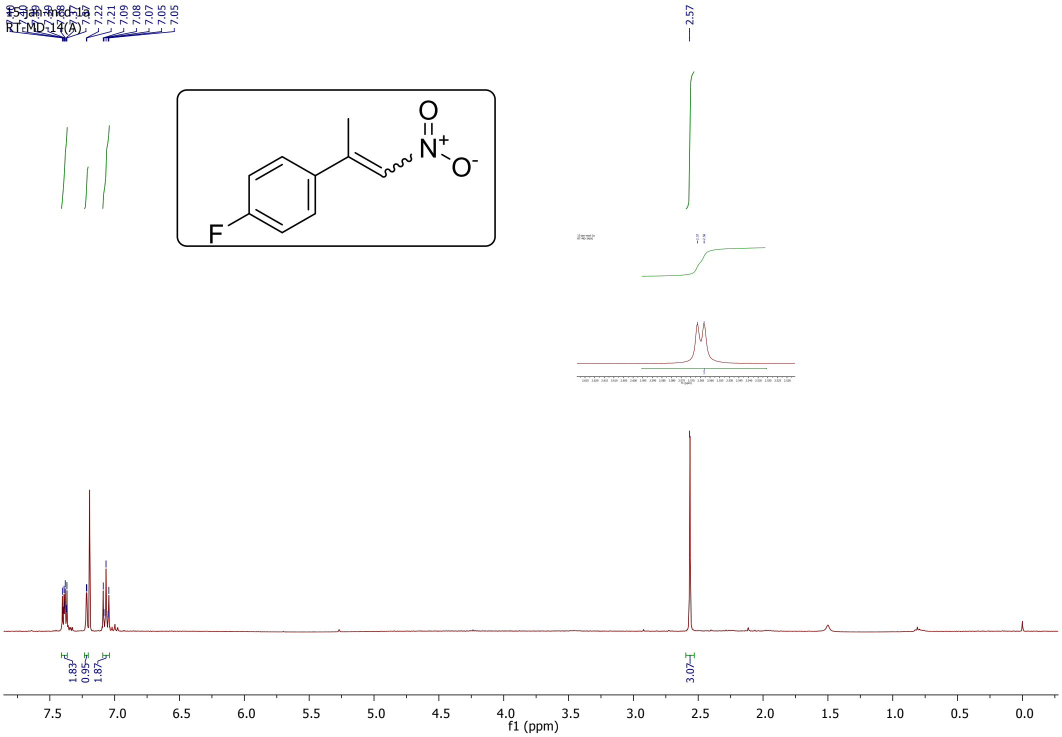

^{13}C NMR of (E/Z)-1-fluoro-4-(1-nitroprop-1-en-2-yl)benzene (2x) [17]

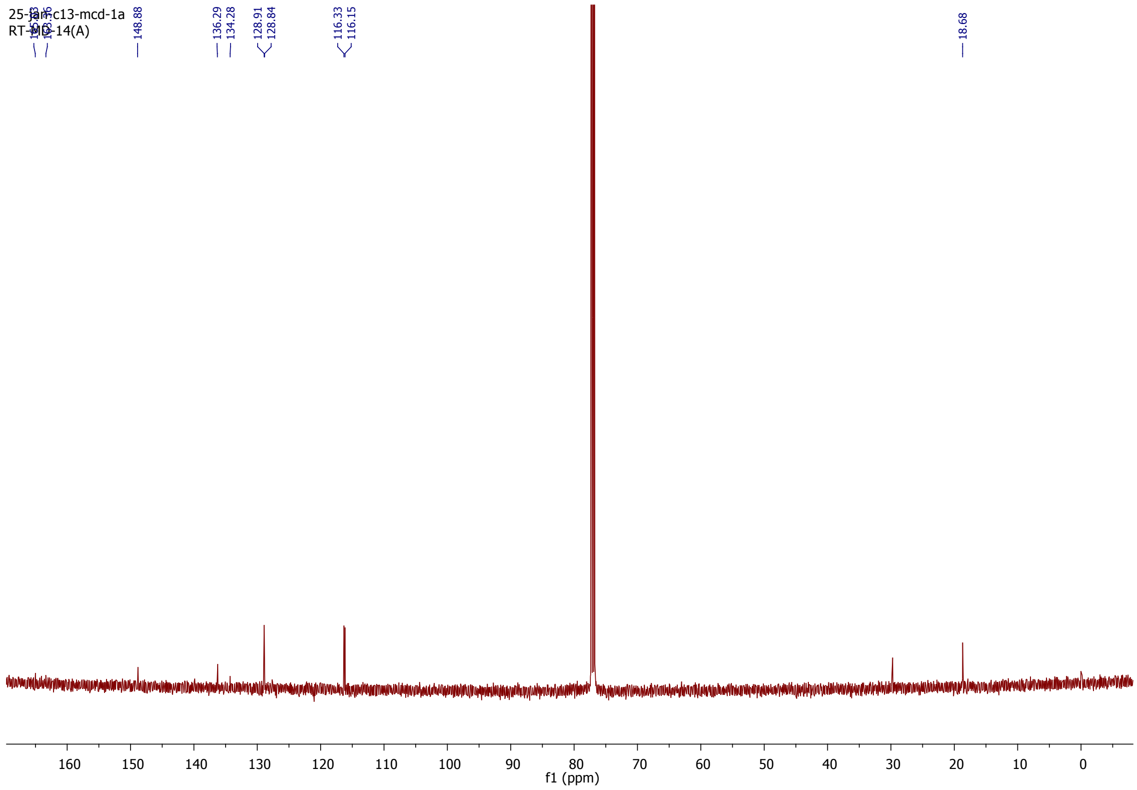

^{1}H NMR of (E/Z)-2-(1-nitroprop-1-en-2-yl)cyclohexa-1,3-diene (2y) [18]

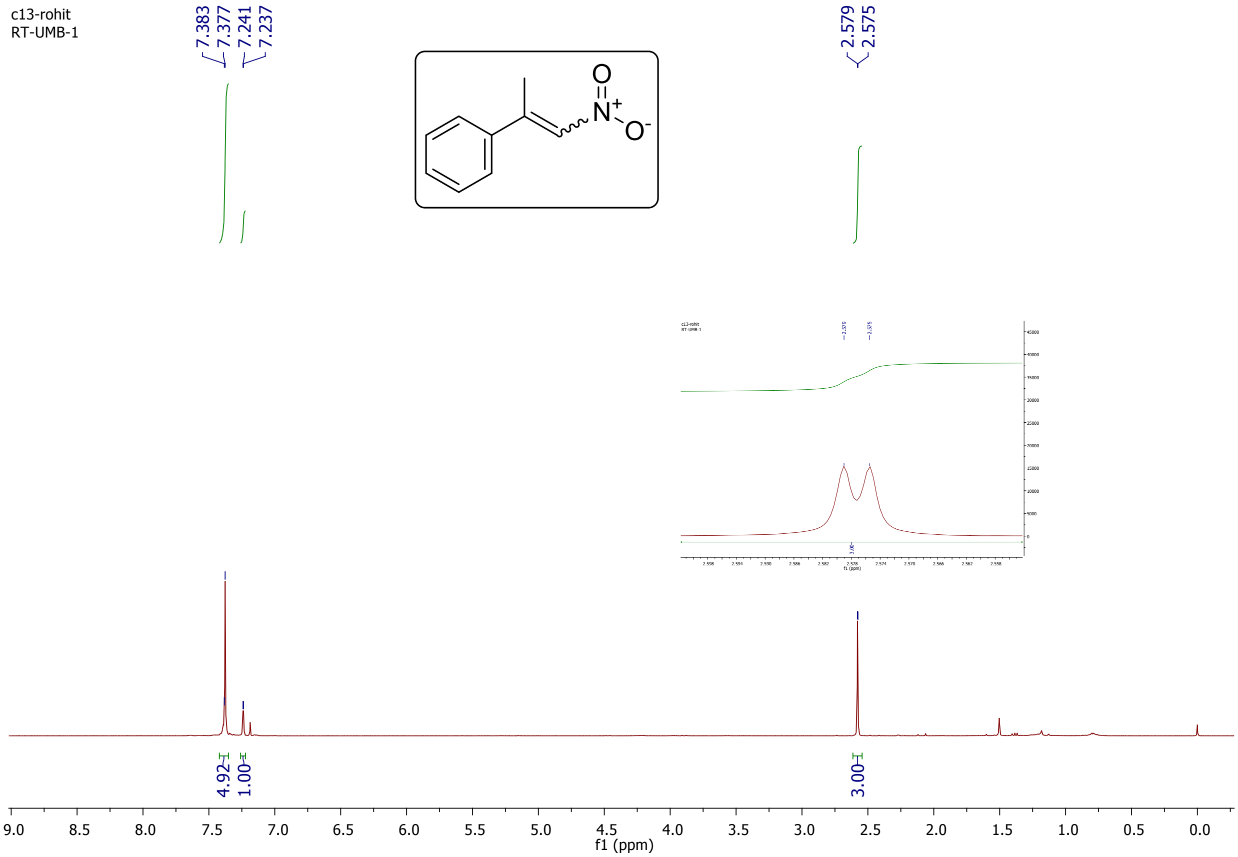

^{13}C NMR of (E/Z)-2-(1-nitroprop-1-en-2-yl)cyclohexa-1,3-diene (2y)

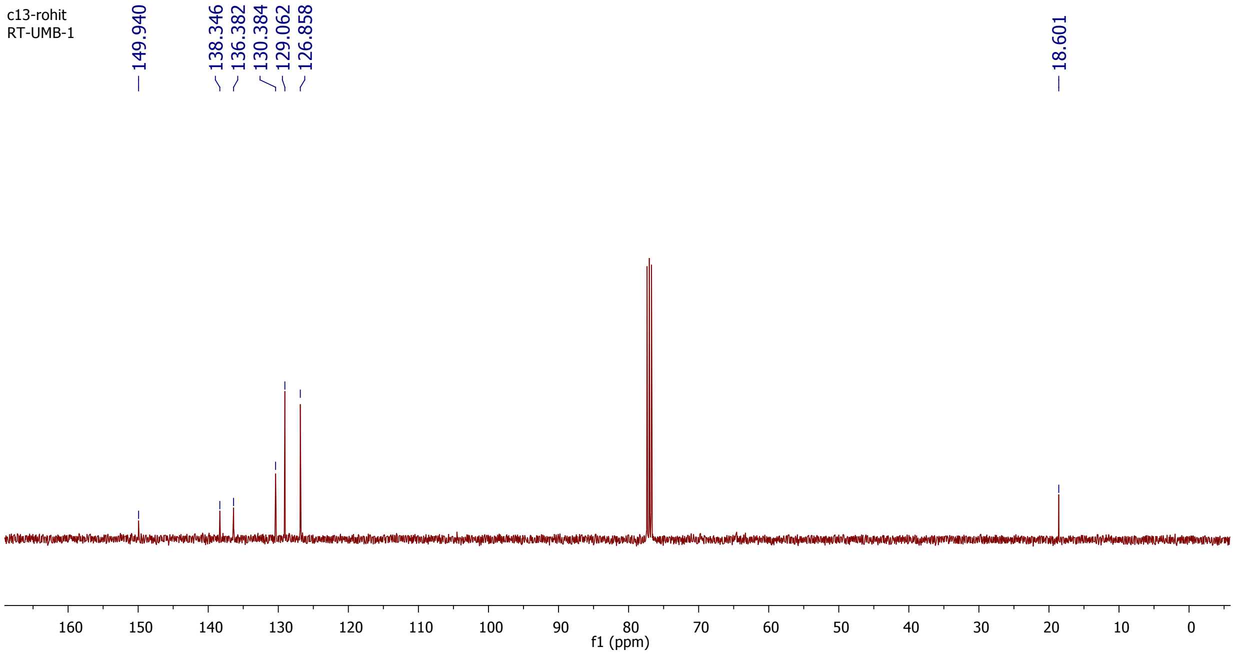

DEPT NMR of (E/Z)-2-(1-nitroprop-1-en-2-yl)cyclohexa-1,3-diene (2y)

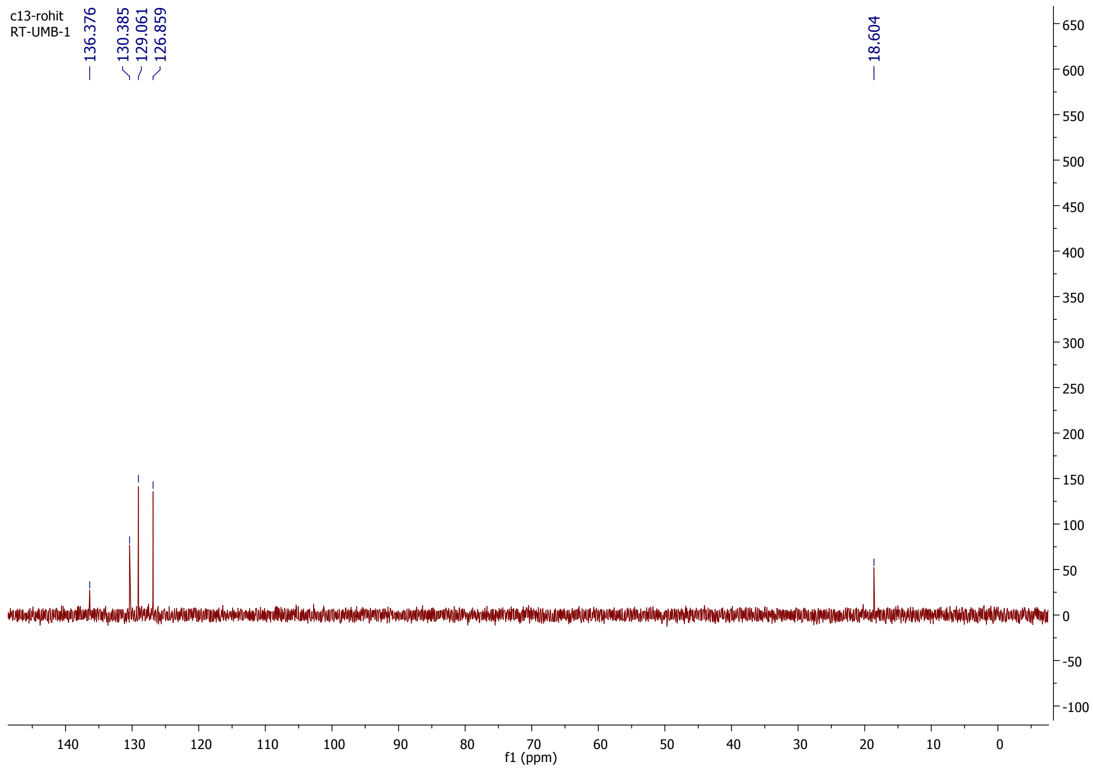

^{1}H NMR of (*E*)-2-(2-Nitrovinyl)furan (4a) [19]

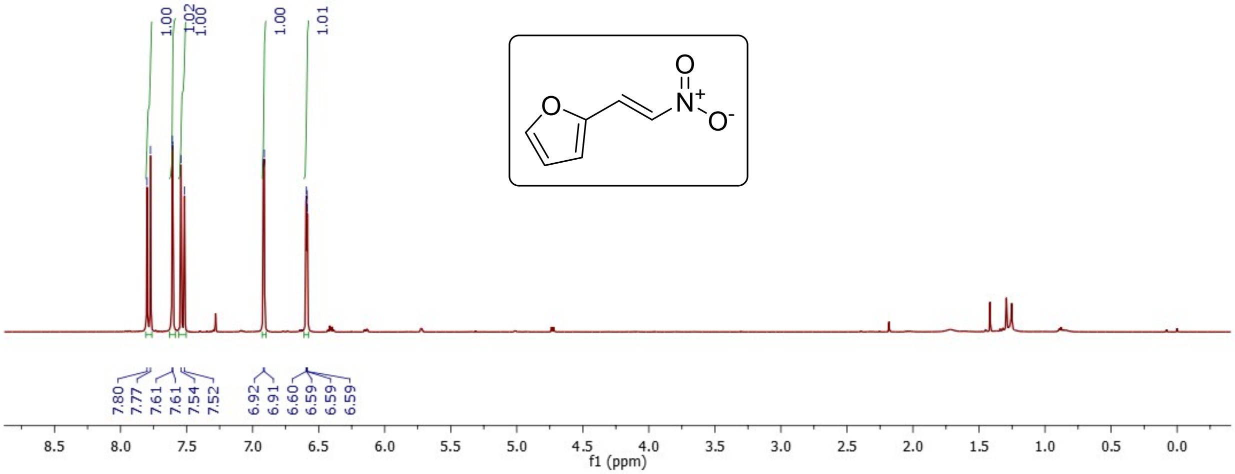

^{13}C NMR of (*E*)-2-(2-Nitrovinyl)furan (4a)

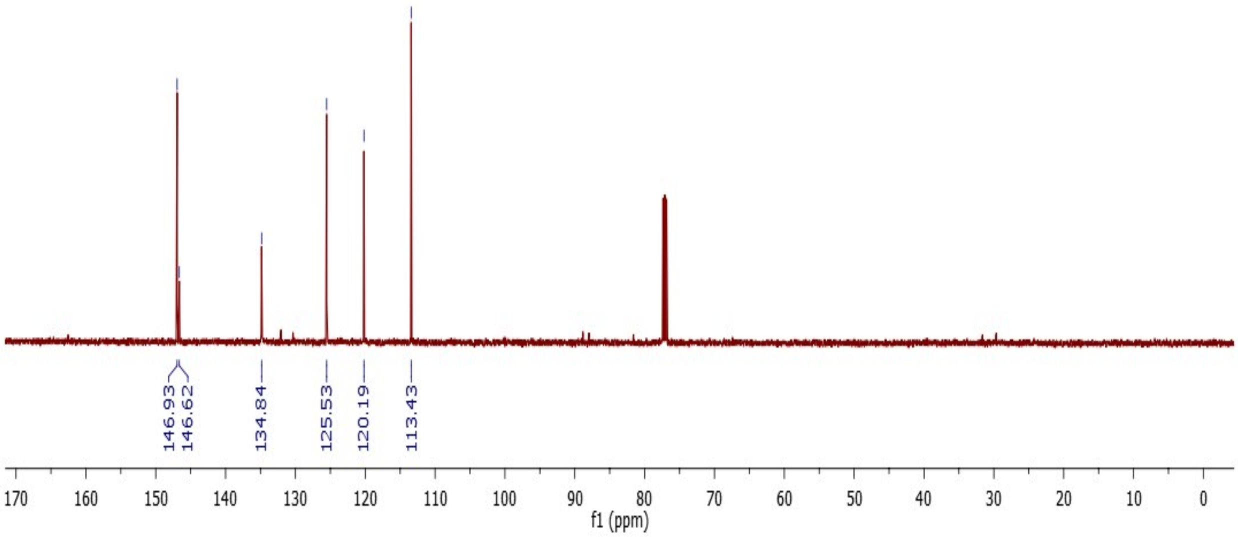

DEPT NMR of (*E*)-2-(2-Nitrovinyl)furan (4a)

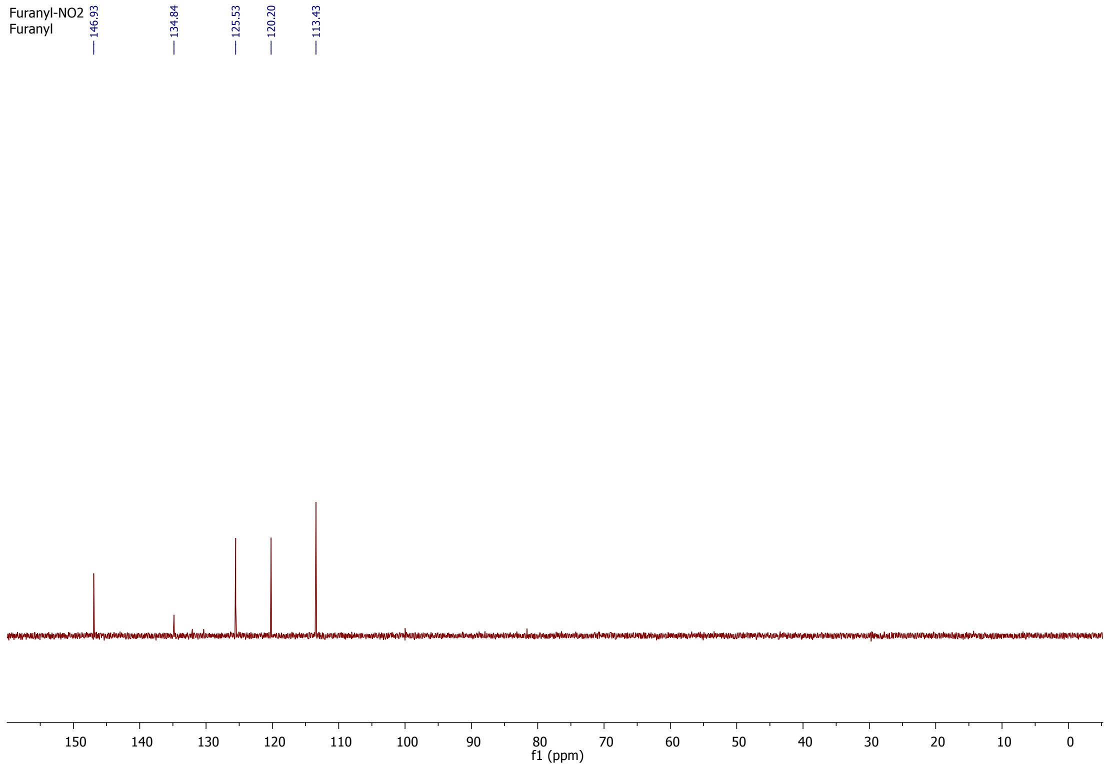

GC-MS of (*E*)-2-(2-Nitrovinyl)furan (4a)

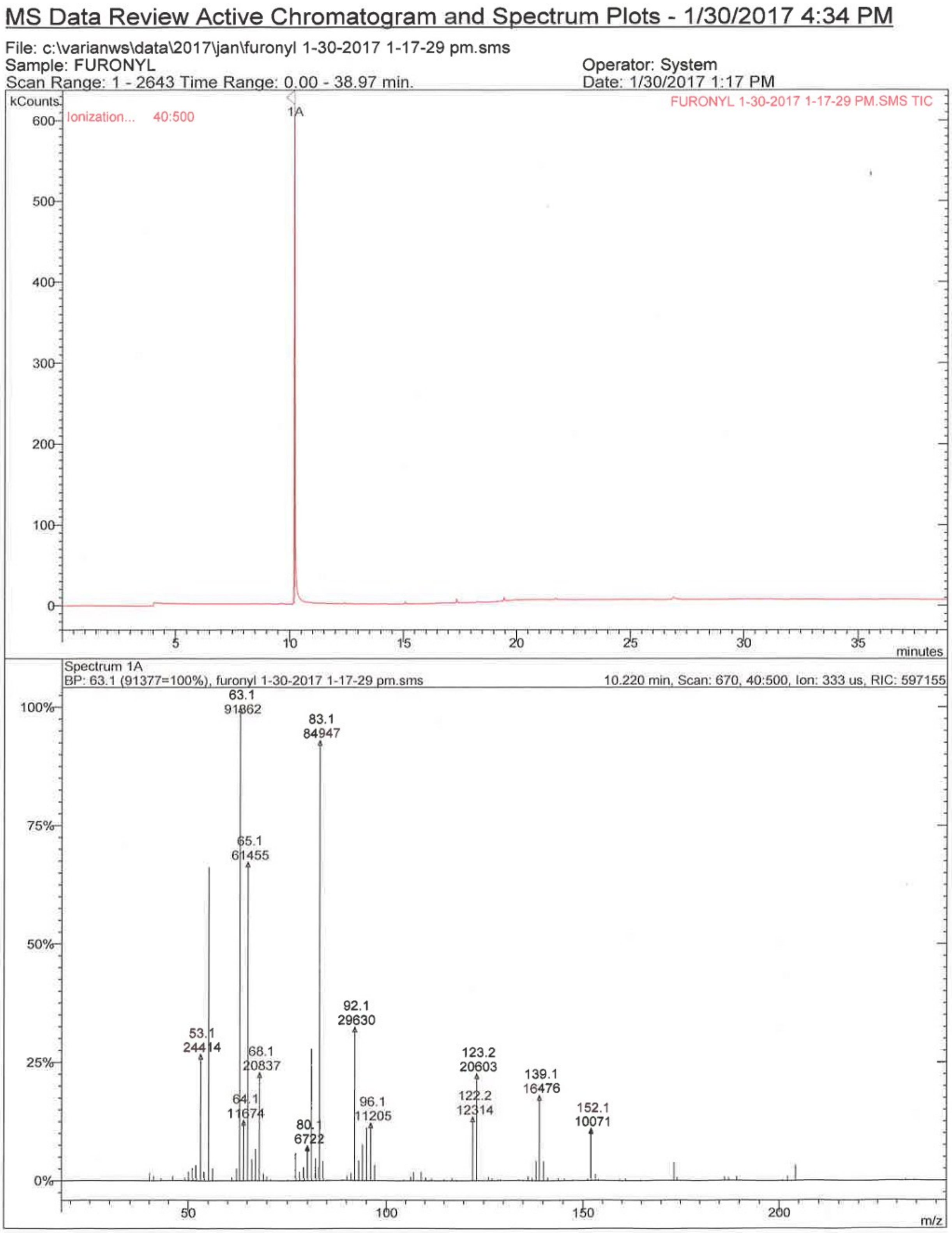

^{1}H NMR of (E)-1-nitronon-1-ene (4b)[20]

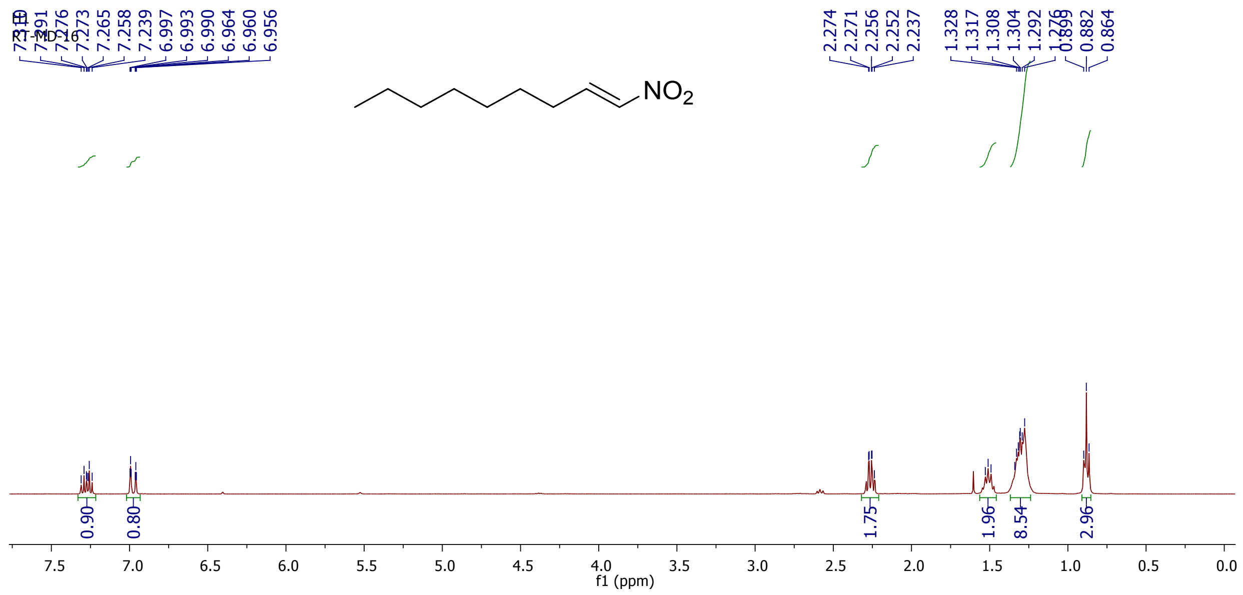

^{13}C NMR of (E)-1-nitronon-1-ene (4b)

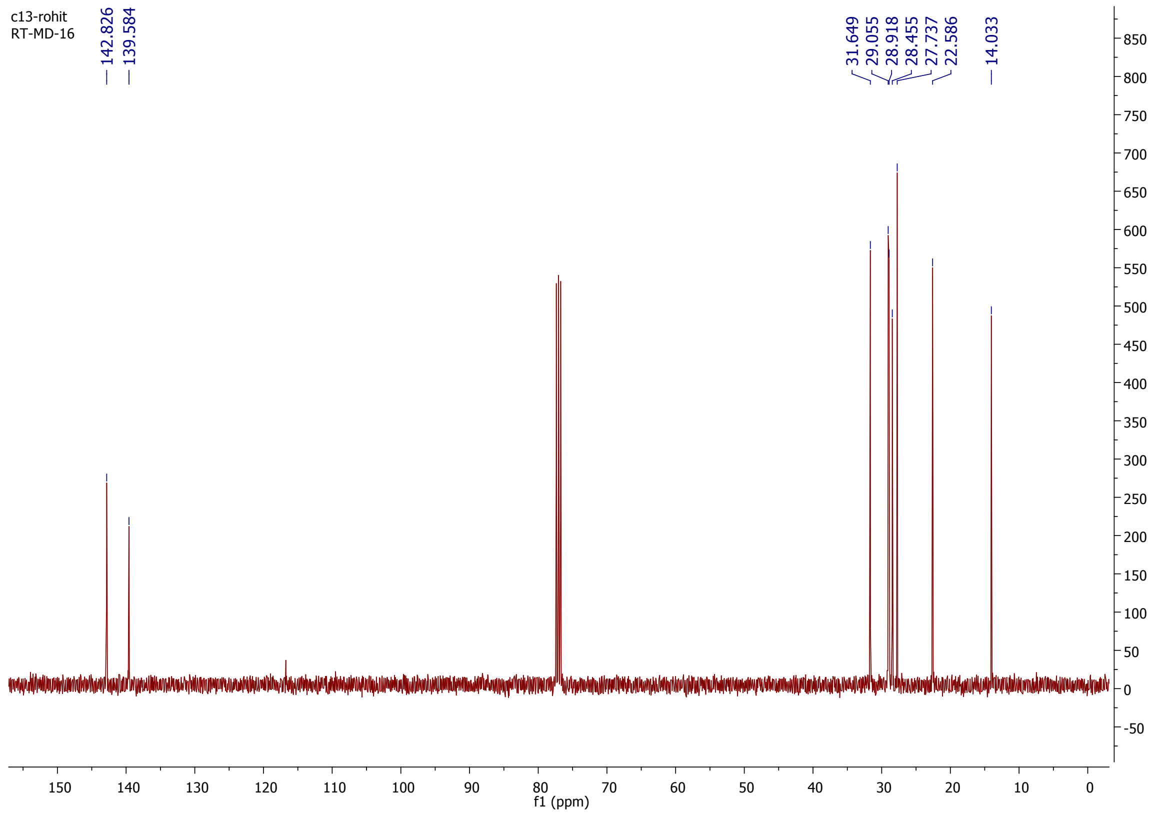

^{1}H NMR of (E)-1-nitrohex-1-ene (4c)[21]

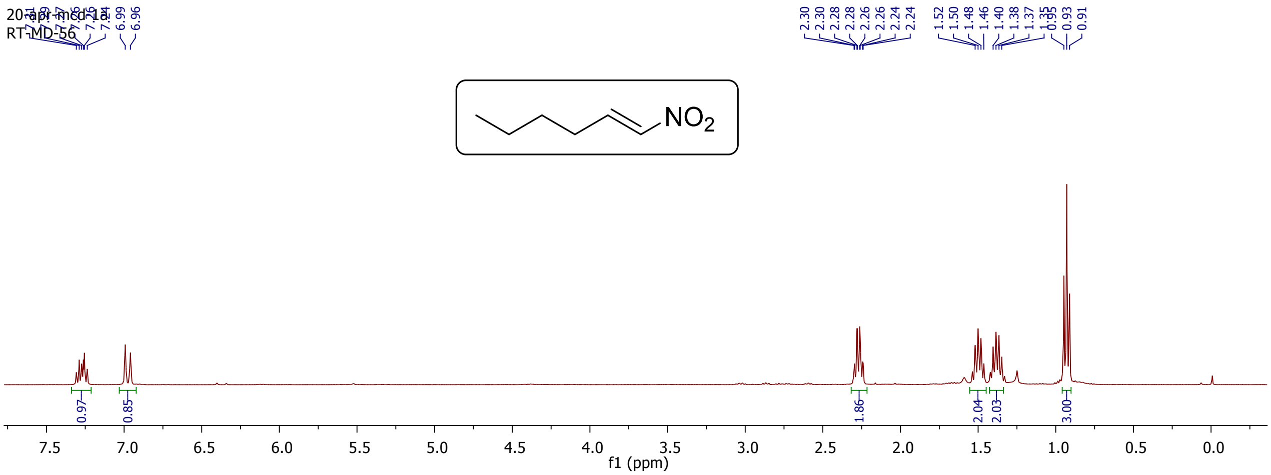

^{13}C NMR of (E)-1-nitrohex-1-ene (4c)

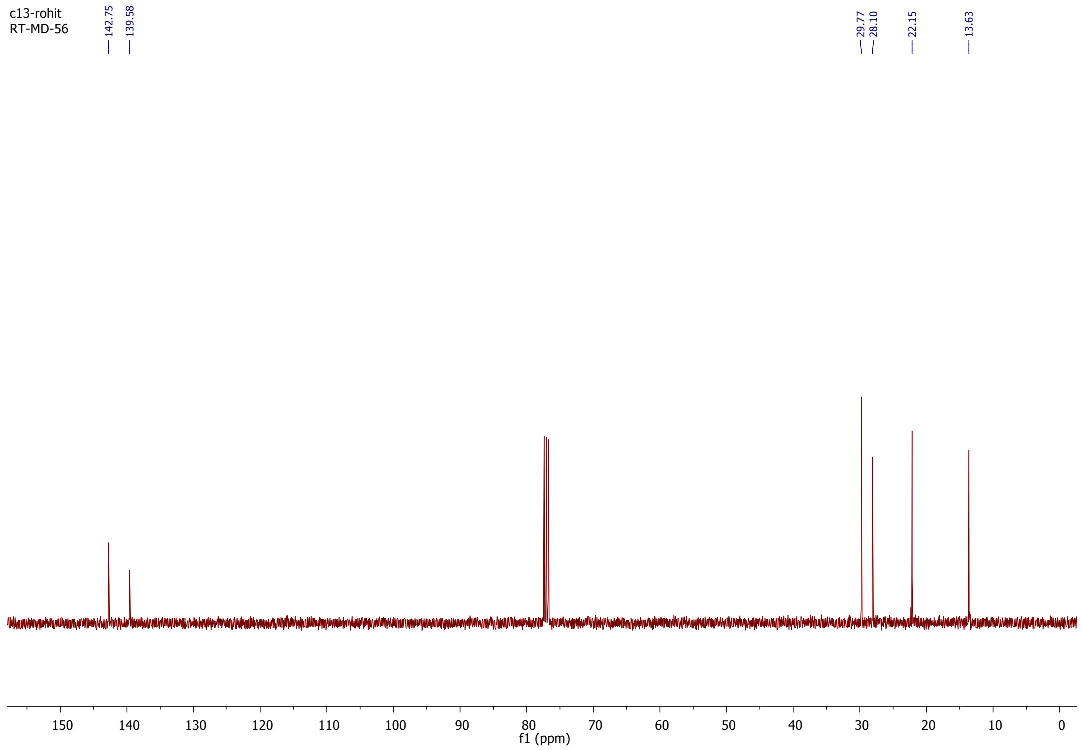

DEPT of (E)-1-nitrohex-1-ene (4c)

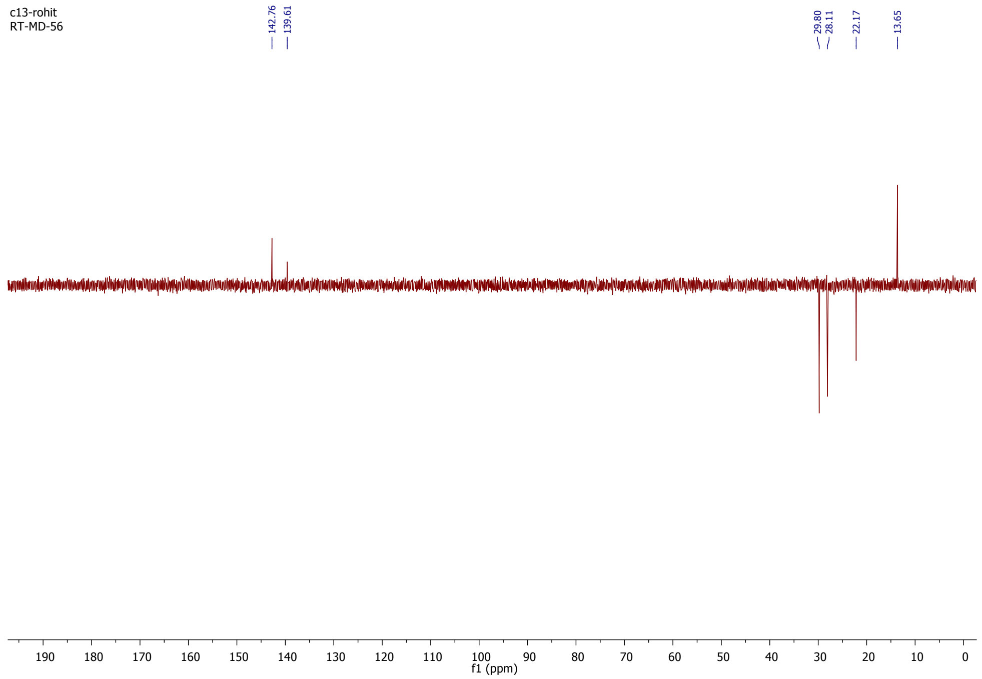

Mass spectra of (E)-1-nitrohex-1-ene 4(c)

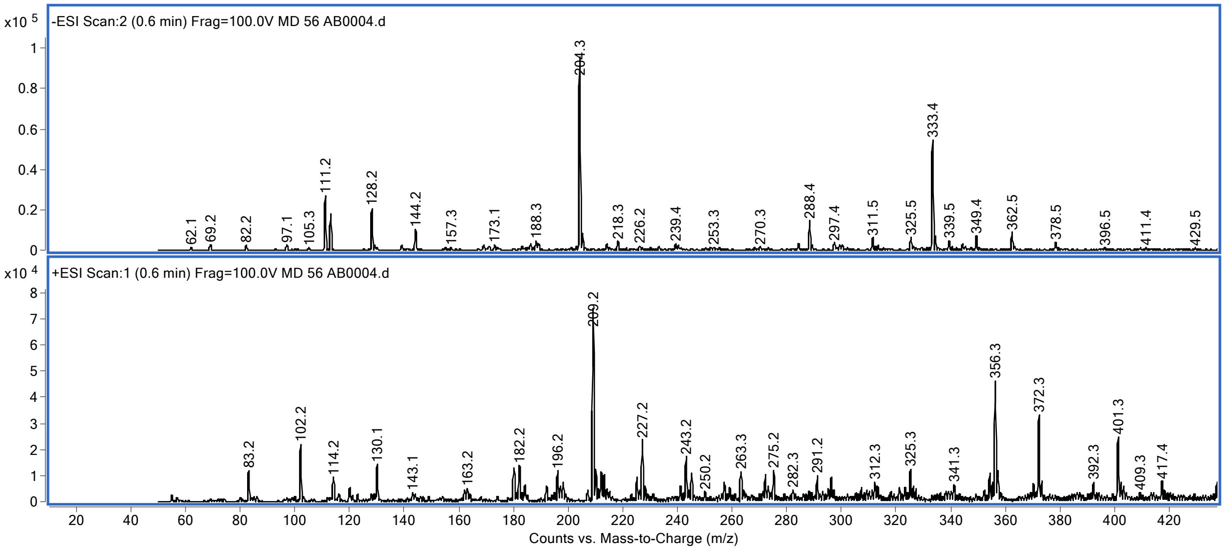

^{1}H NMR of (E/Z)-2-methyl-1-nitronon-1-ene (4d)[22]

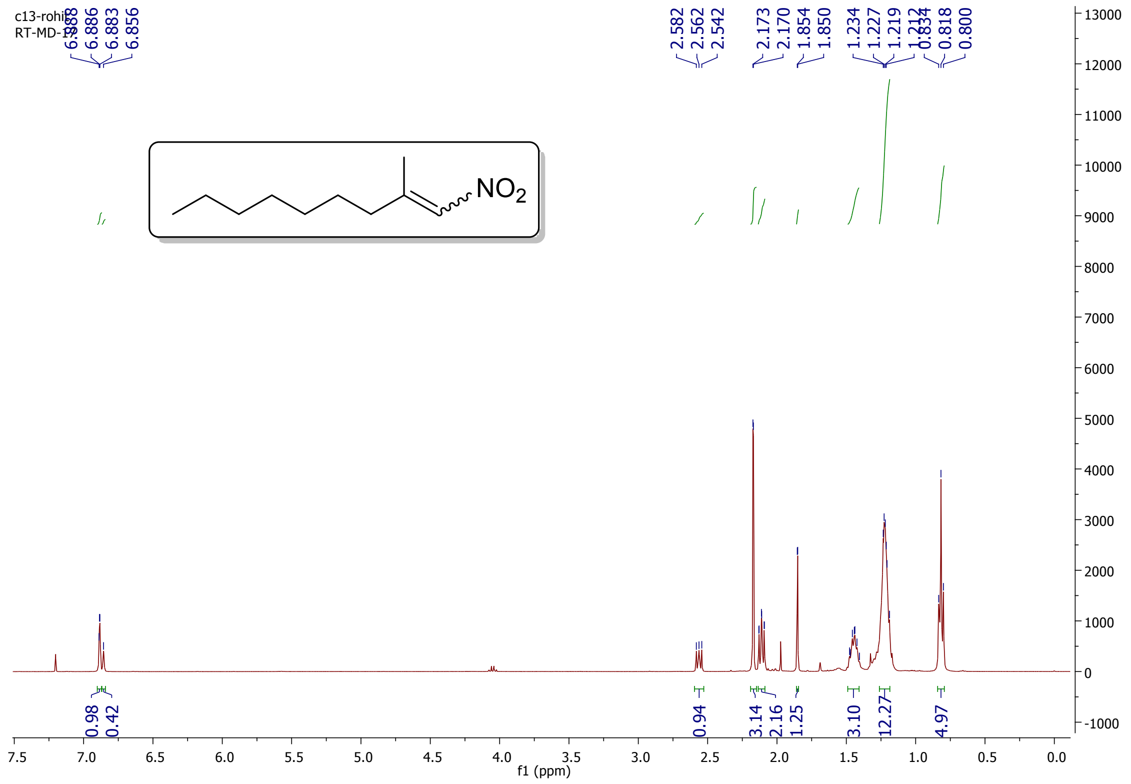

^{13}C NMR of (E/Z)-2-methyl-1-nitronon-1-ene (4d)

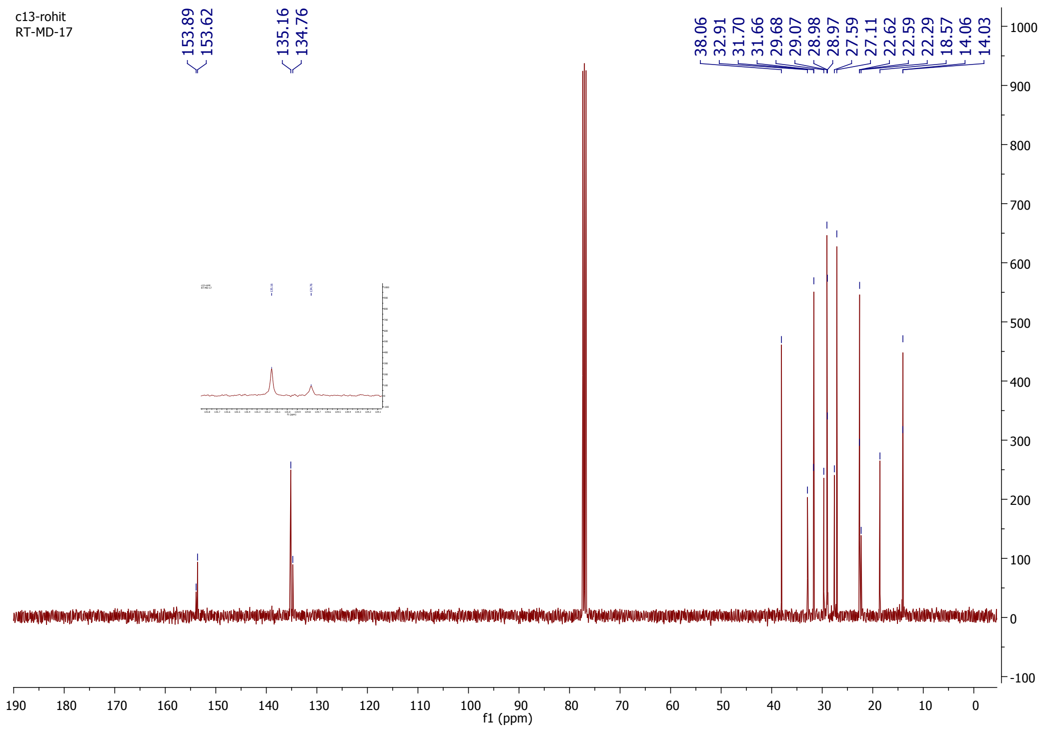

DEPT NMR of (*E/Z*)-2-methyl-1-nitronon-1-ene (4d)

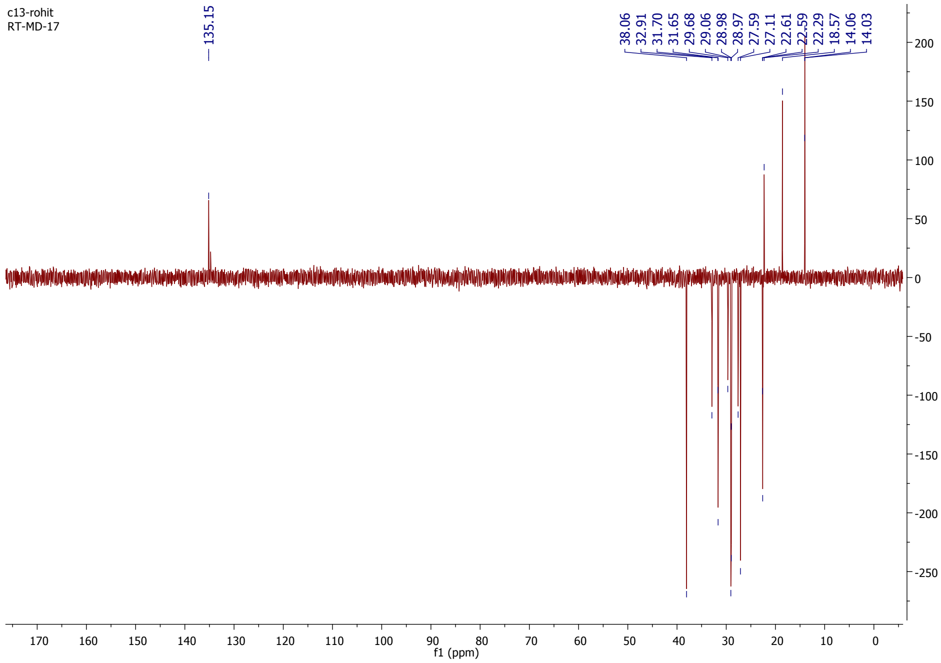

HRMS of (*E/Z*)-2-methyl-1-nitronon-1-ene (4d)

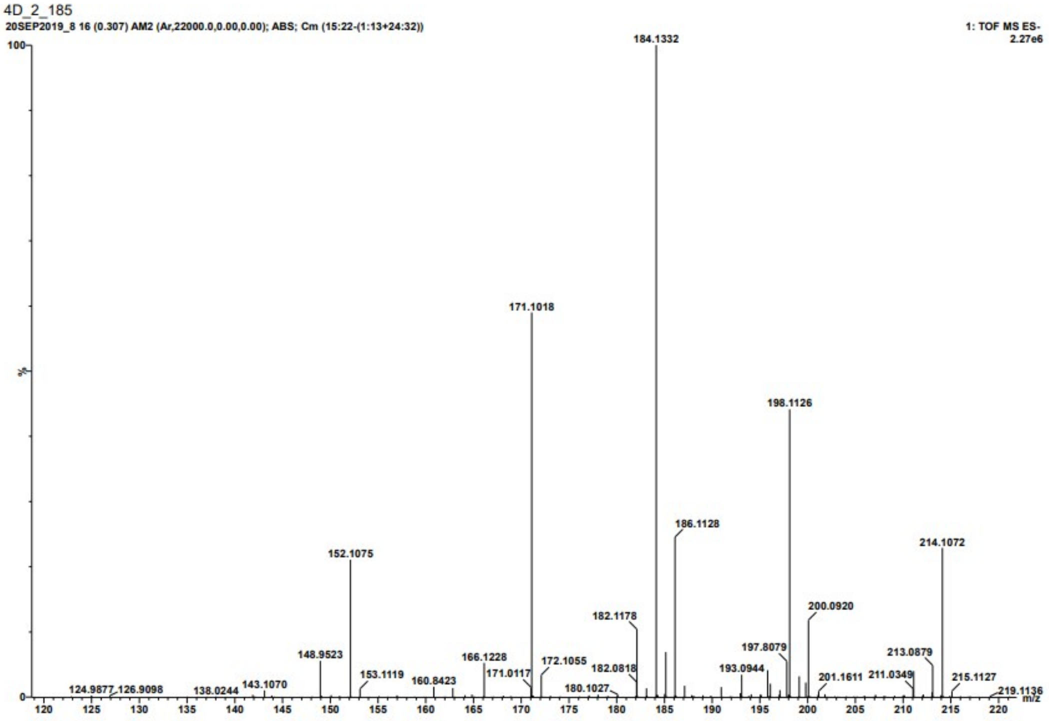

REFERENCES :

1) Maity, S.; Naveen, T.; Sharma, U.; Maiti, D., *Org. Lett.,* **2013**, *15*, 3384.
2) Maity, T.; Naveen, S.; Sharma, U.; Maiti, D., J. Org. Chem. **2013**, 78, 5949.
3) Pettita R.,Pettit G., Hamel E., Hogan F., Moser B., Wolf S., Pon S., Chapuis J., Schmidt J., *Bio-org. & Med. Chem.*, **2009**, 18, 6606.
4) Zhang M., Jun Zhou J., Kan J.,Wang M., Su W., Hong M., *Chem. Comm.*, **2010**, 30, 5455.
5) Dauzonne D., Royer R., *Chem. & Pharm. Bulletin.*, **1986**, 4, 1628
6) Zhao, A.; Jiang, Q.; Jia, J.; Xu, B.; Liu, Y.; Zhang, M.; Liu, Q.; Luo, W.; Guo, C., *Tetrahedron Lett.,* **2016**, *57,* 80.
7) Okino T., Hoashi Y., Furukawa T., Xu X., Takemoto Y., *J. Am. Chem. Soc.*, **2005**, 1, 119.
8) Alizadeh A., Khodaei M., Eshghi A., *J. Org. Chem.*, **2010**, 23, 8295.
9) Popp B., Thorman J., Morales C., Landis C., Stahl S., *J. Am. Chem. Soc.*, **2004**, 45, 14832.
10) Alizadeh A., Khodaei M., Eshghi A., *J. Org. Chem.*, **2010**, 23, 8295.
11) Maity, S.; Manna, S.; Rana, S.; Naveen, T.; Mallick, A.; Maiti, D., *J. Am. Chem. Soc.* **2013**, 135, 3355.
12) J. Am.Chem. Soc., **2004**, 45, 14832.
13) Maity, S.; Manna, S.; Rana, S.; Naveen, T.; Mallick, A.; Maiti, D., *J. Am. Chem. Soc.* **2013**, 135, 3355.
14) Concellón J., Bernad P., Solla H., Concellón C., *J. Org. Chem.*, **2007**, 72, 5421.
15) Maity, S.; Manna, S.; Rana, S.; Naveen, T.; Mallick, A.; Maiti, D., *J. Am. Chem. Soc.* **2013**, 135, 3355.
16) Midya S., Rana J., Abraham T., Bhaskaran A., Aswin B., Balaraman E., *Chem. Comm.,* **2017**, 53, 6760.
17) Fryszkowska A., Fisher K.,Gardiner J., Tephens G., J. Org. Chem., **2008**, 73, 4295.
18) Maity, S., Naveen, T., Sharma, U., Maiti, D., *J. Org. Chem*., **2013**, 78, 5949.
19) Mahmooda S., Lallemanda M., Bakaounia L., Chartona O., Verite P., Dufata H., Tillequin F., *Tetrahedron*, **2004**, 60, 5105.
20) Gorczynski M., Huang J., Lee H., King S., Bio & Med. Chem. Let., **2007**, 17, 2013.
21) Knochel, P.; Seebach, D., *Synthesis*, **1982**, 12, 1017.
22) Concellón J., Bernad P., Solla H., Concellón C., *J. Org. Chem.*, **2007**, 72, 5421.
